# “KAIZEN” method realizing implementation of deep-learning models for COVID-19 CT diagnosis in real world hospitals

**DOI:** 10.1038/s41598-024-52135-y

**Published:** 2024-01-19

**Authors:** Naoki Okada, Yutaka Umemura, Shoi Shi, Shusuke Inoue, Shun Honda, Yohsuke Matsuzawa, Yuichiro Hirano, Ayano Kikuyama, Miho Yamakawa, Tomoko Gyobu, Naohiro Hosomi, Kensuke Minami, Natsushiro Morita, Atsushi Watanabe, Hiroyuki Yamasaki, Kiyomitsu Fukaguchi, Hiroki Maeyama, Kaori Ito, Ken Okamoto, Kouhei Harano, Naohito Meguro, Ryo Unita, Shinichi Koshiba, Takuro Endo, Tomonori Yamamoto, Tomoya Yamashita, Toshikazu Shinba, Satoshi Fujimi

**Affiliations:** 1 Osaka General Medical Center, Osaka, Japan; 2 University of Tsukuba, Tsukuba, Japan; 3 fcuro Inc., Osaka, Japan; 4 Osaka Metropolitan University, Osaka, Japan; 5 Shizuoka Saiseikai General Hospital, Shizuoka, Japan; 6 Shonan Kamakura General Hospital, Kamakura, Japan; 7 Tsuyama Chuo Hospital, Tsuyama, Japan; 8 Teikyo University, Tokyo, Japan; 9 Juntendo University Urayasu Hospital, Urayasu, Japan; 10 Showa University Hospital, Tokyo, Japan; 11 Tokyo Women’s Medical University Hospital, Tokyo, Japan; 12 National Hospital Organization Kyoto Medical Center, Kyoto, Japan; 13 International University of Health and Welfare, School of Medicine, Narita Hospital, Narita, Japan; 14 Nara Prefecture General Medical Center, Nara, Japan; 15 Osaka City General Hospital, Osaka, Japan

**Keywords:** Computational biology and bioinformatics, Diseases, Medical research

## Abstract

Numerous COVID-19 diagnostic imaging Artificial Intelligence (AI) studies exist. However, none of their models were of potential clinical use, primarily owing to methodological defects and the lack of implementation considerations for inference. In this study, all development processes of the deep-learning models are performed based on strict criteria of the “KAIZEN checklist”, which is proposed based on previous AI development guidelines to overcome the deficiencies mentioned above. We develop and evaluate two binary-classification deep-learning models to triage COVID-19: a slice model examining a Computed Tomography (CT) slice to find COVID-19 lesions; a series model examining a series of CT images to find an infected patient. We collected 2,400,200 CT slices from twelve emergency centers in Japan. Area Under Curve (AUC) and accuracy were calculated for classification performance. The inference time of the system that includes these two models were measured. For validation data, the slice and series models recognized COVID-19 with AUCs and accuracies of 0.989 and 0.982, 95.9% and 93.0% respectively. For test data, the models’ AUCs and accuracies were 0.958 and 0.953, 90.0% and 91.4% respectively. The average inference time per case was 2.83 s. Our deep-learning system realizes accuracy and inference speed high enough for practical use. The systems have already been implemented in four hospitals and eight are under progression. We released an application software and implementation code for free in a highly usable state to allow its use in Japan and globally.

## Introduction

Since the first case of severe coronavirus disease 2019 (COVID-19) in Wuhan, China, in December 2019, approximately 766 million people have been infected and 6.93 million deaths have been reported worldwide as of May 31th, 2023 (https://covid19.who.int/). Early detection of infected patients is essential for controlling the spread of severe acute respiratory syndrome coronavirus 2 (SARS-CoV-2)^1^. Although the RT-PCR test is the gold standard for confirming SARS-CoV-2^2,3^, chest CT has been considered a helpful complement^4–7^. Indeed, it has been reported that false negatives with PCR tests are far more common than expected^8^; in some studies, chest CT showed higher sensitivities than PCR tests^6,9–11^. In addition, it is unrealistic to conduct PCR tests for all patients with fever and respiratory failure in the post-pandemic era considering the burden on clinical practice. As in the precedent case of tuberculosis screening^12^, chest CT is expected to become a significant alternative to PCR testing for COVID-19 screening in patients with fever or respiratory failure in near future.

Although CT is useful in diagnosing COVID-19, there are still some problems. For example, radiologists are burdened with interpreting CT when there is a large volume of images^13^. Further, it is difficult for physicians to diagnose based on CT images without sufficient experience with this disease^14^. Researchers have attempted to develop machine learning-based models for diagnosing COVID-19 using CT images to support physicians. More than 2000 AI models for COVID-19 have been developed to decrease the burden on physicians and improve their diagnoses^15^. The model designs vary; for example, distinguishing COVID-19 from normal^16^, COVID-19 from viral pneumonia including influenza^17^, and COVID-19 from other infectious respiratory diseases such as bacterial/viral pneumonia^18^. In addition, the structures of the models vary: some use deep learning^17–20^, others use machine learning methods^16,21,22^, and others use manually designed algorithms^23–25^. However, none of these numerous models have reached a clinically applicable level^15,26^.

The development and application of diagnostic imaging AI models must be conducted based on the steps below, which fully anticipate the context in which the models will be used^15,27–35^:

1. Create an overall picture of the study design based on the appropriate clinical hypothesis.
2. Collect data necessary for the study.
3. Determine an appropriate annotation method to give them the correct answers.
4. Design the AI model properly.
5. Train the model based on the annotated data.
6. Evaluate the accuracy of the trained model.
7. Build an inference environment using this model.

Existing AI models for COVID-19 diagnosis based on CT images have yet to be implemented in hospitals effectively because of the lack of design considerations in these steps. For example, in steps one, two, and six, most previous studies still need to present that their test datasets comprehensively include diseases that should be differentiated from COVID-19^15^. Their models might have been designed to be more accurate in their appearance by excluding diseases that are challenging to differentiate from COVID-19, such as interstitial pneumonia. Indeed, it is revealed that some models are significantly less accurate in real-world hospital data^36^.

Guidelines for developing diagnostic imaging AI models have been created to accomplish these steps and to implement diagnostic imaging AI models optimized for the application place. Several checklists have been proposed for strict criteria that such AI models should meet^27–30^. A representative example is a checklist for artificial intelligence in medical imaging (CLAIM)^31^ presented by Mongan et al. The CLAIM proposed concrete criteria that must be met in Steps 1 through 6. These guidelines focus on the model development process, i.e., the pre-implementation process, and no concrete criteria for Step 7 have been proposed thus far in the medical field. However, it is necessary to create these criteria because there are limitations to the computing environment used in hospitals (either local or cloud) and a requirement for outputting results in a sufficiently short time for not to delay clinical practice. Based on the engineering research^32–35^, we organized the criteria that must be fulfilled in Step 7. The following three items were included in Step 7:

Item 1: Data loading, data formatting, batch size setting, and description of the detailed inference process, including model execution.
Item 2: Hardware, software libraries, and execution environment, including packages.
Item 3: Inference speed or time, and inference performance indicators, including memory consumption during inference.

Items were added to CLAIM to create the “KAIZEN checklist”. AI models were developed for COVID-19 diagnosis from CT images optimized for Japanese clinical situations based on the “KAIZEN checklist”.

Two binary-classification deep-learning models were developed and evaluated. One determines whether a single CT image contains COVID-19 lesions (slice model) and the other determines whether a patient is infected by COVID-19 from a series of chest CT images (series model). The collaboration of these two models makes our AI system explainable, which enables physicians to understand where the AI focuses and to what degree it suspects. Models were implemented in hospitals as software applications. The entire development process was evaluated based on the “KAIZEN checklist” to ensure validity, transparency, and reproducibility.

**Figure 1:**
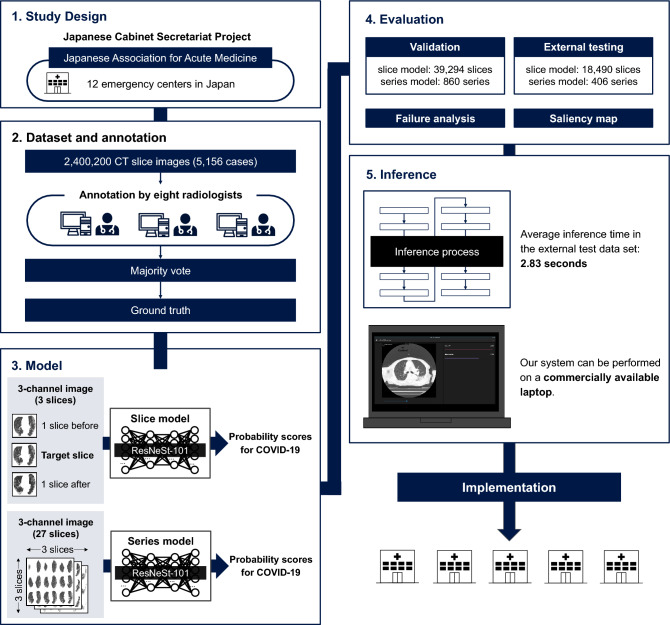
Visual abstract. The overview of our work is described in this figure. A large number of CT images were collected and labeled by eight radiologists. Two binary classification models were trained and evaluated by these image datasets. An inference program to execute these models was constructed and implemented to real-world hospitals. All of the process was conducted based on the “KAIZEN checklist”.

We published the detailed methods of preparing appropriate data, annotation, training, and evaluating models based on the “KAIZEN checklist” (Fig. 1). Further, we developed a public software program to execute these models. We strongly believe that our work will help researchers and developers build AI systems not necessarily in Japan but in areas with different patient backgrounds, types of CT equipment, and other conditions.

## Results

### KAIZEN checklist-based evaluation

The “KAIZEN checklist” was developed based on previous studies^31–35^. In response to this checklist, all the research processes were evaluated on each item (Table 1). The corresponding parts of this paper and appendices are cited for every item.

**Table 1:** Summary of the evaluation of our research based on each item of the “KAIZEN checklist.”

Section/topic	No.	Item	Status	Described at
Title/abstract				
	1	Identification as a study of AI methodology, which specifies the category of technology used (e.g., deep learning)	✓	Abstract
	2	Structured summary of study design, methods, results, and conclusions	✓	Abstract
Introduction				
	3	Scientific and clinical background, which includes the intended use and clinical role of the AI approach	✓	“Introduction”
	4	Study objectives and hypotheses	✓	“Introduction”
Method				
Study design	5	Prospective or retrospective study	✓	“Methods”
	6	Study goal such as model creation, exploratory study, feasibility study, and non-inferiority trial	✓	“Introduction” “Discussion”
Data	7	Data sources	✓	“Methods” Supplementary Sect. 1
	8	Eligibility criteria: how, where, and when potentially eligible participants or studies were identified (e.g., symptoms, results from previous tests, inclusion in registry, patient-care setting, location, dates)	✓	“Methods” Supplementary Sect. 1
	9	Data pre-processing steps	✓	“Methods” Supplementary Sect. 7
	10	Selection of data subsets, if applicable	✓	“Methods” Supplementary Sect. 5
	11	Definitions of data elements with references to Common Data Elements	✓	“Methods” Supplementary Sect. 6 Supplementary Sect. 8
	12	De-identification methods	✓	Supplementary Sect. 4
	13	How missing data are handled	✓	Supplementary Sect. 5
Ground truth	14	Definition of ground truth reference standard in sufficient detail to allow replication	✓	“Methods” Supplementary Sect. 6
	15	Rationale for selecting the reference standard (if alternatives exist)	✓	“Methods” Supplementary Sect. 6
	16	Source of ground-truth annotations; qualifications and preparation of annotators	✓	Supplementary Sect. 6
	17	Annotation tools	✓	Supplementary Sect. 6
	18	Measurement of inter- and intrarater variability; methods to mitigate variability and/or resolve discrepancies	✓	“Methods” Supplementary Sect. 6 Supplementary Sect. 11
Data partitions	19	Intended sample size and how it was determined	✓	Supplementary Sect. 1
	20	How data are assigned to partitions; specify proportions	✓	“Methods” Supplementary Sect. 5
	21	Level at which partitions are disjoint (e.g., image, study, patient, institution)	✓	Supplementary Sect. 5
Model	22	Detailed description of the model, including inputs, outputs, all intermediate layers, and connections	✓	“Methods”
	23	Software libraries, frameworks, and packages	✓	Supplementary Sect. 7 Supplementary Sect. 8
	24	Initialization of model parameters (e.g., randomization, transfer learning)	✓	Supplementary Sect. 8
Training	25	Details of training approach, including data augmentation, hyperparameters, and number of models trained	✓	Supplementary Sect. 8
	26	Method of selecting the final model	✓	Supplementary Sect. 8
	27	Ensembling techniques, if applicable	–	N/A
Evaluation	28	Metrics of model performance	✓	“Methods”
	29	Statistical measures of significance and uncertainty (e.g., confidence intervals)	✓	Supplementary Sect. 11
	30	Robustness or sensitivity analysis	✓	“Results” Supplementary Sect. 14
	31	Methods for explainability or interpretability (e.g., saliency maps), and how they were validated	✓	“Results” Supplementary Sect. 9
	32	Validation or testing on external data	✓	“Methods” Supplementary Sect. 1 Supplementary Sect. 5
Results				
Data	33	Flow of participants or cases using a diagram to indicate inclusion and exclusion	✓	“Results” Supplementary Sect. 5
	34	Demographic and clinical characteristics of cases in each partition	✓	Supplementary Sect. 12
Model performance	35	Performance metrics for optimal model(s) on all data partitions	✓	“Results” Supplementary Sect. 13
	36	Estimates of diagnostic accuracy and their precision (such as 95% confidence intervals)	✓	“Results” Supplementary Sect. 11 Supplementary Sect. 13
	37	Failure analysis of incorrectly classified cases	✓	“Results” Supplementary Sect. 14
Discussion				
	38	Study limitations including potential bias, statistical uncertainty, and generalizability	✓	“Discussion”
	39	Implications for practice, which include the intended use and/or clinical role	✓	“Discussion”
Other information				
	40	Registration number and name of registry	✓	“Methods”
	41	Where the full study protocol can be accessed	✓	“Methods”
	42	Sources of funding and other support; role of funders	✓	“Methods”
Inference				
	43	Detailed description of the inference process; data loading, data formatting, batch size setting, model execution	✓	Supplementary Sect. 7 Supplementary Sect. 10
	44	Hardware specification, software libraries, frameworks, and packages	✓	Supplementary Sect. 10
	45	Evaluation of inference performance including inference speed or time and memory consumption	✓	“Results” Supplementary Sect. 15

For each item, we added information about which part of this paper or supplement describes the details.

### Patients characteristics and image datasets

Data acquisition was limited inside Japan because the priority was implementing a system optimized for the application place: Japanese clinical settings. We comprehensively collected CT images of COVID-19 pneumonia, all other lung diseases (bacterial/viral pneumonia, atypical pneumonia, pulmonary edema, COPD, interstitial lung diseases, tumor, hemorrhage, and trauma), and normal cases on a large scale from 12 emergency centers through the Japanese Association for Acute Medicine between April 1, 2017 and January 31, 2021 (Supplementary Sect. 1).

**Figure 2:**
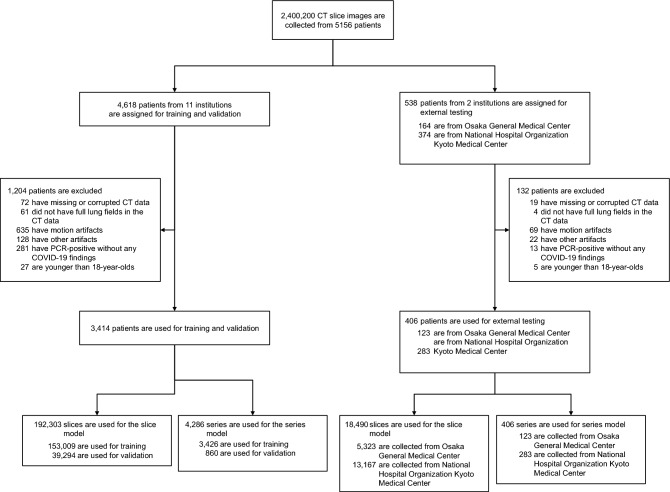
Flowchart of the process for inclusion and exclusion of the collected patients’ data. After exclusion, only CT images at the initial imaging of each patient are included in the slice model. All CT series, including follow-up, are included in the series model.

2,400,200 CT images were retrospectively collected from 5156 patients, 1644 with COVID-19, 2607 with other lung diseases, and 905 normal, with a mean age of 64.3 (range: 7–104, median: 69), and 60.2% males. For training and validation, we used 3414 patient images randomly split into a training dataset (80%) and a validation dataset (20%) using the Hold-out method^37^: 153,009 and 39,294 slices for the slice model and 3426 and 860 series including follow-up for the series model, respectively. For external testing, we used images of 406 patients consecutively collected from Osaka General Medical Center and Kyoto Medical Center: 18,490 slices for the slice model and 406 series (only the initial imaging of each patient) for the series model (Fig. 2, Supplementary Sect. 5). All test cases were presented to emergency centers with fever or respiratory failure, and they were COVID-19 suspects at the time. There were no leaks concerning cases between training, validation, and testing (Supplementary Sect. 1).

The patients’ demographics in each partition are summarized in Table 2 (Supplementary Sect. 12 for details on the demographics per institution and patients’ disease demographics used in the test data).

**Table 2:** Summary of the demographics of patients in each partition.

Slice model							
	Patients	Slices	Males	Females	Age (minimum)	Age (maximum)	Age (average)
Training data							
COVID-19	798 (29.2%)	23,873 (15.6%)	510 (63.9%)	288 (36.1%)	19	95	62.0
OLD	1424 (52.1%)	84,102 (55.0%)	871 (61.2%)	553 (38.8%)	18	101	65.7
Normal	510 (18.7%)	45,034 (29.4%)	257 (50.4%)	253 (49.6%)	18	97	60.3
Total	2732	153,009	1638 (60.0%)	1094 (40.0%)	18	101	63.6
Validation data							
COVID-19	187 (27.4%)	5472 (13.9%)	126 (67.4%)	61 (32.6%)	23	93	61.1
OLD	352 (51.6%)	21,402 (54.5%)	215 (61.1%)	137 (38.9%)	18	99	66.3
Normal	143 (21.0%)	12,420 (31.6%)	76 (53.1%)	67 (46.9%)	18	93	62.1
Total	682	39,294	417 (61.1%)	265 (38.9%)	18	99	64.0
External test data							
COVID-19	120 (29.6%)	5294 (28.6%)	85 (70.8%)	35 (29.2%)	34	94	70.2
OLD	156 (38.4%)	6843 (37.0%)	98 (62.8%)	58 (37.2%)	24	98	73.3
Normal	130 (32.0%)	6353 (34.4%)	70 (53.8%)	60 (46.2%)	18	102	59.5
Total	406	18,490	253 (62.3%)	153 (37.7%)	18	102	68.0

Series model							
	Patients	Series	Males	Females	Age (minimum)	Age (maximum)	Age (average)
Training data							
COVID-19	787 (28.8%)	1400 (40.9%)	506 (64.3%)	281 (35.7%)	19	95	61.6
OLD	1422 (52.0%)	1498 (43.7%)	871 (61.3%)	551 (38.7%)	18	101	65.5
Normal	524 (19.2%)	528 (15.4%)	271 (51.7%)	253 (48.3%)	18	95	60.9
Total	2733	3426	1648 (60.3%)	1085 (39.7%)	18	101	63.5
Validation data							
COVID-19	198 (29.1%)	347 (40.3%)	130 (65.7%)	68 (34.3%)	22	94	62.4
OLD	354 (52.0%)	384 (44.7%)	215 (60.7%)	139 (39.3%)	18	94	67.3
Normal	129 (18.9%)	129 (15.0%)	62 (48.1%)	67 (51.9%)	18	97	60.1
Total	681	860	407 (59.8%)	274 (40.2%)	18	97	64.5
External test data							
COVID-19	120 (29.6%)	120 (29.6%)	85 (70.8%)	35 (29.2%)	34	94	70.2
OLD	156 (38.4%)	156 (38.4%)	98 (62.8%)	58 (37.2%)	24	98	73.3
Normal	130 (32.0%)	130 (32.0%)	70 (53.8%)	60 (46.2%)	18	102	59.5
Total	406	406	253 (62.3%)	153 (37.7%)	18	102	68.0

“COVID-19,” “Other lung diseases (OLD),” and “Normal” represent COVID-19-positive patients, patients with other lung diseases, and patients without any detected respiratory diseases, respectively.

“Patients,” “Slices,” and “Series” represent the number of unique patients, the number of images used for development and evaluation of the slice model, and the number of samples used for the development and evaluation of the series model, respectively.

### Reliability of ground truth

The CT images were labeled as COVID-19 negative if their case was lastly confirmed as COVID-19 negative by the on-site physician through CT findings and other clinical data including PCR and follow-up examinations. The PCR-positive cases except those confirmed COVID-19 negative were grouped by the institutions (further subdivided internally for institutions with a large number of cases). Their CT slice images were labeled as COVID-19 positive or negative independently of each other according to the COVID-19 Reporting and Data System (CO-RADS)^38^. Each slice image was scored independently by three different radiologists to obtain a majority vote. The labeling agreement rates were evaluated for each subgroup. The overall agreement rate was 0.657 (95% confidence interval [CI] 0.642–0.673; interpretation [IP]: substantial), with a maximum agreement rate of 0.781 (95% CI 0.732–0.831; IP: substantial), and a minimum of 0.432 (95% CI 0.374–0.490; IP: moderate) (Supplementary Sect. 6).

### AI system architecture

**Figure 3:**
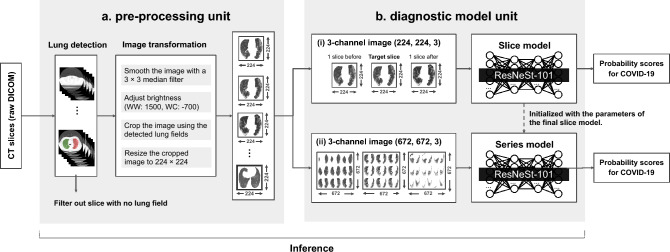
AI system architecture. Raw DICOM images are standardized and molded in the pre-processing unit. These images are input into each of the models in the diagnostic model unit to output probability scores for COVID-19.

Our AI system consists of two units: a pre-processing unit, a diagnostic model unit (Fig. 3). The characteristics of CT images differ based on the imaging equipment, institutions, and technicians. All CT images are subjected to pre-processing in a slice-by-slice manner before being input into the models to standardize such differences. Lung fields are detected from slice images and then cropping, smoothing, brightness adjustment, and resizing are applied. Lungmask^39^, an open-source software tool, is used to detect the lung fields; a median filter is used to smooth the images. The window values are adjusted to a window width of 1500 and a window center of –700 Hounsfield Unit (HU)^40,41^; the size is changed to 224 × 224 (Supplementary Sect. 7).

Two binary-classification deep-learning models, the slice model and the series model exist in the diagnostic model unit. The slice model determines whether a CT image contains COVID-19 lesions, and the series model determines whether a patient is infected by COVID-19 from a series of chest CT images. These two models were designed to output probability scores for COVID-19 in the range of 0–1. Input images for the slice model include three pre-processed slice images: the target slice and the slices before and after. This gives the slice model peripheral information about the target slice and enables it to deal with ambiguous lesions^42^. The input for the series model is comprised of 27 pre-processed slice images selected from entire chest CT images to have equal intervals in the axial section. These slices are then arranged in 3 × 3 × 3 three-dimensional grids from the front upper left to the back lower right corner to give the series model 3D information^43^. The basic structure for both models is ResNeSt-101^44^ (“Methods”).

### Model performance

**Figure 4:**
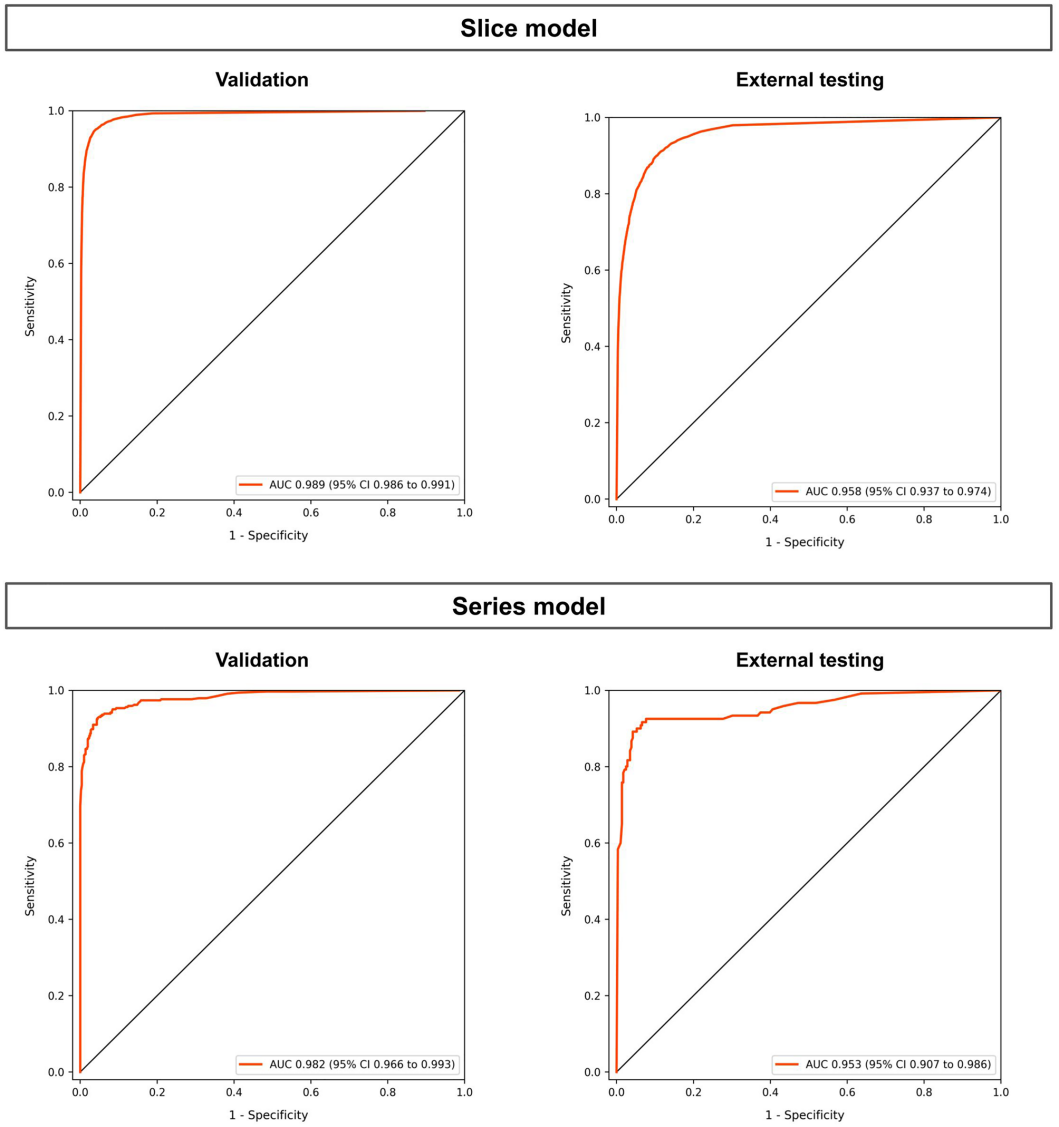
ROC curves of the slice and series models. The ROC curves of the slice and series models for the validation and test data are shown in Fig. 3. The AUC values and their 95% confidence intervals are also shown.

In the validation dataset, the slice model distinguished COVID-19 images from other lung diseases and normal images with an AUC of 0.989 (95% CI 0.986–0.991). With a threshold of 0.5, the sensitivity was 90.3% (95% CI 89.5–91.1), the specificity was 98.1% (95% CI 98.0–98.2), and the accuracy was 97.0% (95% CI 96.9–97.2). The series model classified COVID-19 with an AUC of 0.982 (95% CI 0.966–0.993). With a threshold of 0.5, the sensitivity was 91.6% (95% CI 88.5–94.5), the specificity was 95.7% (95% CI 94.0–97.5), and the accuracy was 94.0% (95% CI 92.4–95.7). The sensitivity, specificity, and accuracy results at the different threshold values for the series and slice models, sensitivity-oriented models and specificity-oriented models, are presented in Table 3 and Fig. 4.

**Table 3:** Classification performance measures for different thresholds.

Threshold	Accuracy [95% CI]	Sensitivity [95% CI]	Specificity [95% CI]
Validation dataset			
Slice model			
0.115	95.2 [95.0–95.4]	95.7 [95.2–96.2]	95.1 [94.8–95.3]
0.165	95.9 [95.7–96.0]	95.1 [94.5–95.6]	96.0 [95.8–95.3]
0.5	97.0 [96.9–97.2]	90.0 [89.5–91.1]	98.1 [98.0–98.2]
Series model			
0.255	93.0 [91.3–94.7]	95.1 [92.5–97.1]	91.6 [89.1–94.0]
0.43	94.3 [92.7–95.9]	93.0 [90.2–95.7]	95.1 [93.2–96.9]
0.5	94.0 [92.4–95.7]	91.6 [88.5–94.5]	95.7 [94.0–97.5]
Test dataset			
Slice model			
0.115	89.5 [87.9–91.0]	90.4 [87.3–93.1]	89.2 [87.2–91.0]
0.165	90.0 [88.5–91.6]	88.4 [85.0–91.3]	90.6 [88.8–92.3]
0.5	91.4 [90.0–92.7]	80.3 [75.7–84.2]	95.0 [93.6–96.3]
Series model			
0.255	91.4 [88.8–94.1]	92.5 [87.6–96.7]	90.9 [87.6–94.1]
0.43	92.9 [90.3–95.3]	91.7 [86.3–96.4]	93.4 [90.4–96.2]
0.5	92.9 [90.3–95.2]	90.0 [84.2–95.0]	94.1 [91.2–96.6]

“Threshold” represents the slice and series models’ threshold values for separating COVID-19 positive and negative. For each threshold, the sensitivity, specificity, and accuracy of the models for the validation and test dataset are shown in the table with their 95% confidence intervals.

For the external consecutively collected test dataset, the slice model detected COVID-19 with an AUC of 0.958 (95% CI 0.937–0.974). With a threshold of 0.5, the sensitivity was 80.3% (95% CI 75.7–84.2), the specificity was 95.0% (95% CI 93.6–96.3), and the accuracy was 91.4% (95% CI 90.0–92.7). The series model detected COVID-19 with an AUC of 0.953 (95% CI 0.907–0.986). With a threshold of 0.5, the sensitivity was 90.0% (95% CI 84.2–95.0), the specificity was 94.1% (95% CI 91.2–96.6), and the accuracy was 92.9% (95% CI 90.3–95.2). The sensitivity, specificity, and accuracy results at the different threshold values for the series and slice models, sensitivity-oriented models and specificity-oriented models, are presented in Table 3.

### Failure analysis of the models

The series model misclassified 28 patients (6.9%) in the test dataset with a threshold of 0.5. Among these patients, 12 had false-negative results, five had emphysema, four had pleural effusions, and one had a hiatal hernia. A total of 16 false-positive cases were observed: five bacterial pneumonia, two viral pneumonia, one atypical pneumonia, five interstitial lung disease, one lung tumor, and two normal cases. Among the false-positive cases, four had emphysema, two had pleural effusions, and two had inflammatory changes.

With the same threshold, the slice model was incorrect in 1620 slices (8.8%). Among these, 996 slices were false-negative and 654 were false-positive. There were 40 patients (9.8%) with a vast number of slices misidentified by the slice model: more than 20% slices of the entire chest of one case or more than 50% slices of all the COVID-19 positive slices of one case. Further, seven positive cases and eight negative cases misclassified by the series model had a high percentage of misidentification with the slice model (Supplementary Sect. 14).

### Saliency maps of the models

DeGrave et al. pointed out that validation using external data alone is insufficient for evaluating the model’s robustness and interpretability evaluation is necessary^45^. In this study, the model interpretability was verified by generating saliency maps using the method proposed by Simonyan et al.^46^.

Supplementary Fig. 9.1a–e show the saliency maps of the slice model. Supplementary Fig. 9.1a and b show the saliency maps for COVID-19. The slice model responded to ground-glass opacities and nodules in image (a). The slice model did not respond to dorsal consolidation or pleural effusion but to ground-glass opacities and nodules in image (b). Supplementary Fig. 9.1c and d show saliency maps for cases of pneumonia other than COVID-19. Similarly, the slice model responded to ground-glass opacities and nodules in these cases. Supplementary Fig. 9.1e shows the saliency maps for the normal case. In this case, the slice model responds to linear opacities.

Supplementary Fig. 9.2a–e show saliency maps of the series model. Supplementary Fig. 9.2a and show saliency maps for COVID-19. The series model did not respond to dorsal consolidation or pleural effusion but responded to ground-glass opacities and nodules. Supplementary Fig. 9.2c and d show saliency maps for cases of pneumonia other than COVID-19. Similarly, the series model responded to ground-glass opacities and nodules in these cases. Supplementary Fig. 9.2e shows the saliency maps for the normal case.

### Inference performance

**Figure 5:**
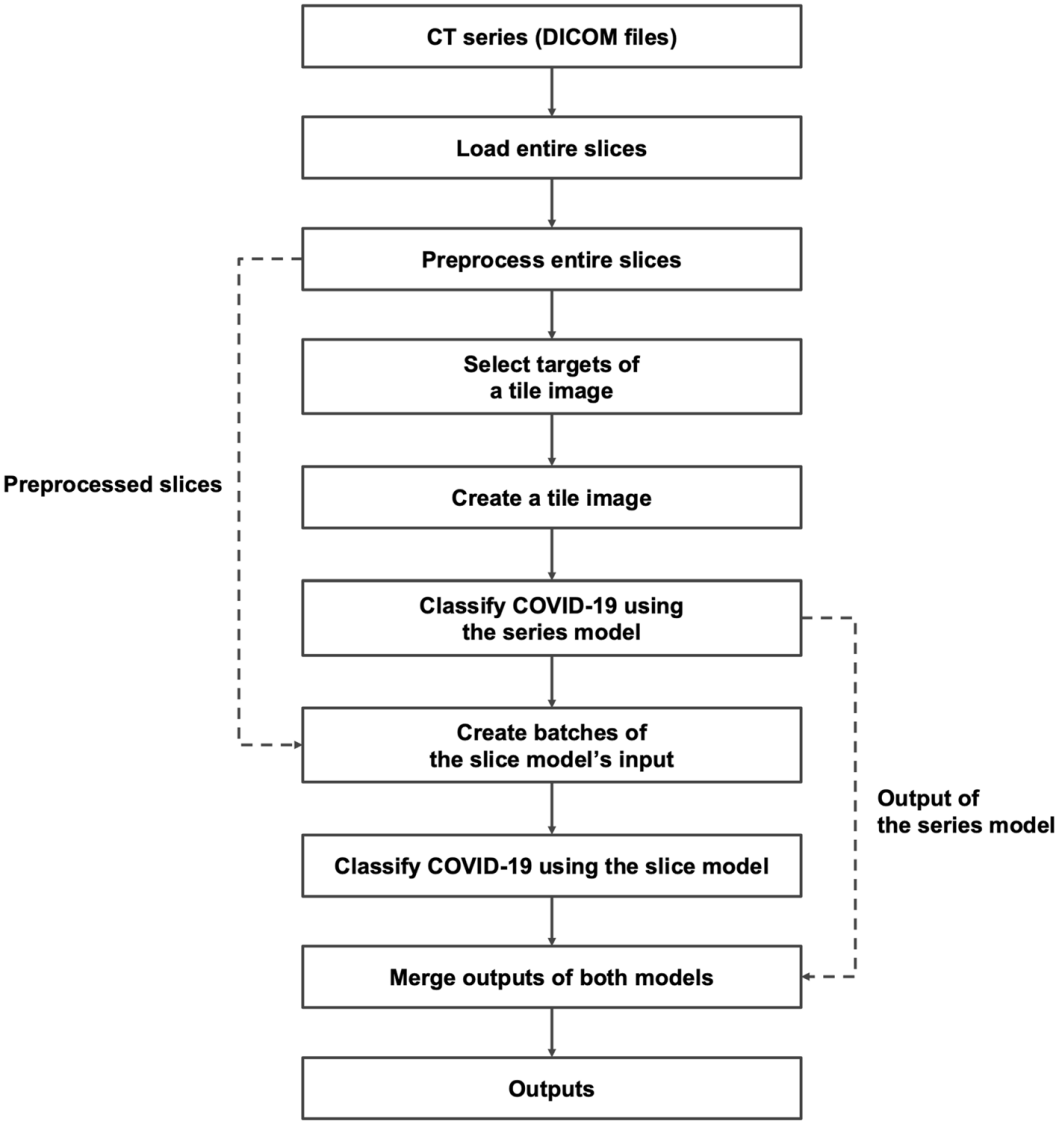
Flowchart of our inference process. Dashed arrows indicate the use of outputs in the past steps.

The inference process was designed as a single common sequence of data loading, data formatting, and execution of each model to obtain the output of the slice model and series model simultaneously (Fig. 5).

A commercially available GPU-equipped laptop machine (Razer RZ09-03305J43-R3J1, 2.30 GHz octa-core Intel Core i7-10875H CPU, 16 GB DDR4 RAM, NVIDIA GeForce RTX 2080 Super with Max-Q Design GPU and 8 GB of GDDR6 VRAM) was used for this inference process.The inference time and memory consumption during the inference were measured under these conditions (Supplementary Sect. 10).

When inference without ingenuity was performed for each model independently, the series model output resulted in an average of 2.58 s (95% CI 2.53–2.63) per series, with a maximum of 3584 MiB of system memory consumption and 1639 MiB of GPU memory consumption. The slice model output results in an average of 11.31 s (95% CI 11.11–11.51) per series, with a maximum of 3485 MiB of system memory consumption and 1511 MiB of GPU memory consumption. In contrast, our improved inference process obtained outputs for both the slice and series models from the same data in an average of 2.83 s (95% CI 2.79–2.88) per series, with a maximum consumption of 3680 MiB of system memory and 3961 MiB of GPU memory (Supplementary Sect. 15).

## Discussion

This is the first study to develop a diagnostic imaging AI system based on predefined rigorous criteria: “the KAIZEN checklist”. This makes our AI system uniquely consistent. In addition, this study is the first to focus on the necessity of inference for diagnostic imaging AI^15,26^, which realizes the implementation of our system in real-world hospitals. Since previous models have not been validated to work on moderate computers and output results quickly, they cannot be applied in hospitals^47^. Our models were developed based on a comprehensive dataset from patients of various ages with various diseases that should be differentiated from COVID-19. This dataset enables our models to recognize mild COVID-19 cases, COVID-19 cases with comorbidities, and pseudo COVID-19 cases such as interstitial lung diseases, pulmonary edema, and atypical pneumonia. The previous AI models cannot recognize these cases because they were never trained or validated by them^48^. In addition, we released the models, their construction methods, and the application software so that our models could be optimized and used worldwide (Supplementary Sect. 16).

The developed deep-learning system can classify COVID-19 accurately (accuracy of 91.4% for the slice model, 92.9% for the series model) in a very short time (2.83 s on average) from the external test dataset CT images of all patients presented to the emergency department. We published the test dataset in an anonymized DICOM format to benchmark it against other AI diagnostic systems.

In the test dataset, 57.1% of the misclassified patients in the series model (either false-negative or false-positive) had pleural effusion or structural changes in the lung such as emphysema, bulla, significant fibrosis, and other old inflammatory changes. The radiologists concluded that some of the other false-negative cases were nonspecific. Most of the other false-positive cases were interstitial lung diseases, which include eosinophilic pneumonia, pneumocystis pneumonia, drug-induced interstitial pneumonia, and silicosis. Further, we examined all these cases with radiologists and confirmed that they had highly similar features to COVID-19. The slice model misidentified the lesion’s upper and lower edges, inflammatory scarring at the apex of the lung, motion artifacts, fibrosis, and atelectasis at the base of the lung. Further, the misclassification was common in slices with frosted grassy shadows because of pulmonary edema and old inflammatory changes. The slices were also challenging to diagnose for radiologists and other physicians. From the saliency maps, dorsal infiltrative shadows were excluded from the regions of interest in both the series and slice models regardless of whether the patients were COVID-19 cases or controls. Both models were assumed to recognize COVID-19 lesions based on the increased concentrations derived from ground-glass opacities. This suggests that it is unlikely they were overfitted with the characteristics of individual institutions or the CT equipment of different manufacturers.

Our system encourages collaboration between physicians and AI^49^. Each slice image can be reviewed with reference to the output of the slice model along with the output results of the series model (Supplementary Sect. 16). Thus, physicians can recognize suspected patients in a moment using the series model output and which part of the case is suspected to be COVID-19 pneumonia with the assistance of the slice model. This system allows physicians to understand AI outputs and focus on essential imaging findings.

**Figure 6:**
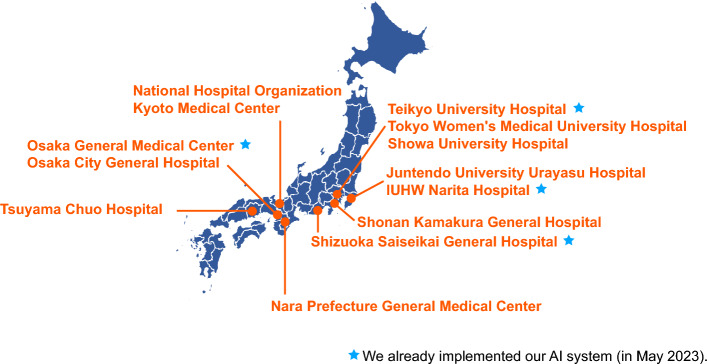
Hospitals that implement our AI system.

Our system is designed to be operated on a non-dedicated laptop to facilitate use at clinical sites. To achieve high computational efficiency in our inference environment, the basic structure for the models is selected to ResNeSt-10144 which delivers high accuracy despite having a relatively low number of parameters. The system can output results in a short time without interrupting clinical workflow, even using a limited computing environment^50^. It has been implemented at the Osaka General Medical Center, Shizuoka Saiseikai General Hospital, Teikyo University Hospital and IUHW Narita Hospital. It is also being implemented at all other partner research institutions (Fig. 6).

The results of this research were published on Zenodo (10.5281/zenodo.5835313) as a Japanese Cabinet Secretariat project, which allowed our deep-learning system to be available for noncommercial use to help end the global crisis caused by COVID-19. In addition, assuming the case where our system does not perform as well as in Japan in some instances because of differences in ethnicity and other conditions such as CT equipment, we included enough information in this paper so that everyone can retune the models by only collecting and annotating CT images from their area^51^. The series model can be tuned only with patient-level labels (COVID-19 or not) without slice-level annotations.

Our study has several limitations, which are listed below:

1. Although the dataset is extensive and covers COVID-19 and its differential diseases, it is limited to the Japanese population. It has not been validated for accuracy in other countries with different ethnic groups, demographics, and CT equipment manufacturers. Therefore, collecting additional data at the application site and tuning the models to increase the accuracy under different circumstances will be necessary.
2. Although we trained and validated the models by removing cases containing artifacts, there are scenarios wherein images with artifacts must be used for diagnosis in clinical practice. In the future, it will be necessary to absorb the effects of artifacts through proper pre-processing steps or to collect a large number of cases containing artifacts and train the models to adapt.
3. There is a residual risk of bias in the annotation because radiologists scored slices based on the assumption that cases containing those slice images were PCR-positive. Therefore, it may have resulted in obtaining higher scores.
4. Although a large dataset was created, the class design was limited to two classes because the number of samples in each category was still insufficient and disproportionate when detailed classifications were made for each type of lung disease. This resulted in only the COVID-19 risk score as the output of the models.
5. There was a risk of producing erroneous outputs if lesions were found only in slices that were not extracted because the series model was based on 27 slices extracted from the entire series as input.
6. The slice model can produce erroneous outputs depending on how the lesion is cropped in the slice because the model does not have 3D information as input.
7. Saliency maps of the implemented models were evaluated only in a qualitative way. We did not evaluate them in a quantitative way.
8. The items about Step 7 in the KAIZEN checklist are based on engineering standards. Further examination might be required applying them to the medical field.

In conclusion, we show that deep-learning models can accurately discriminate COVID-19 patients from non-COVID-19 patients using CT images if they are developed following rigorous criteria. There was no implementable COVID-19 diagnostic imaging AI in previous studies due to methodological flaws. While this system is useful for screening COVID-19 patients because it can be used immediately after CT imaging and provides output in about 3 s, the physician's eye remains essential to pick up COVID-19 patients missed by the system and to eliminate false-positive patients. Future prospective clinical trials are essential for demonstrating the safety and efficacy of diagnostic imaging AI technology. We strongly believe that the universally applicable “KAIZEN Checklist” and our models are facilitating the implementation of not only COVID-19 AI but of future pandemic respiratory diseases.

## Methods

### Ethical approvals, registration

This study was approved by Osaka General Medical Center Clinical medicine Ethics Committee (IRB: 2020-073), which waived the requirement for written informed consent because of the retrospective nature and minimal risk to subjects of this study. It was conducted following the principles of the Declaration of Helsinki. The summary of this study was posted at all participating institutions. This study was registered with the Japan Registry of Clinical Trials (jRCT1050210089).

### Role of the funding source

This study was conducted under the budget of the Japanese Cabinet Secretariat project (https://www.covid19-ai.jp/en-us/, 438-2020-5E, 834-2021-4A, 847-2022-2C, 847-2022-2D). The funders were not involved in the design of the study, its interpretation, or the writing of the paper. The corresponding author is responsible for all the work performed.

### Image datasets

In addition to the COVID-19 pneumonia, other lung diseases (bacterial/viral pneumonia, atypical pneumonia, pulmonary edema, COPD, interstitial lung diseases, tumor, hemorrhage, and trauma) and normal cases were comprehensively collected from multiple institutions. The details of the CT equipment characteristics at all institutions are presented in Supplementary Sect. 3. Data was gathered at the Osaka General Medical Center in the form of anonymized DICOM data (Supplementary Sect. 4). Axial slice images with a thickness of 3–7 mm were used^52^.

Cases with corrupted or duplicate data, without complete lung fields, with artifacts in the lung fields, with devices of the procedure in the thorax, younger than 18 years of age, and COVID-19 cases without significant findings recorded by radiologists were excluded (Fig. 2).

### Ground truth

Images were labeled as COVID-19 positive if the case was PCR-positive and had some CT findings of COVID-19 reported by radiologists. The images were scored independently of each other into five stages of certainty corresponding to findings presented in the COVID-19 Reporting and Data System (CO-RADS). This was completed by eight radiologists who did not directly treat the patients and were given only the images. CO-RADS has six categories according to the degree of COVID-19 certainty and category six was excluded because it was defined as PCR-positive^38^.

Each slice image was scored independently by three different radiologists to obtain a majority vote. Images of the training and validation dataset that failed to gain a majority vote or were noted as challenging to diagnose by even one radiologist were double-checked at the radiologist conferences (comprising at least three board-certified radiologists with more than ten years of clinical experience) at the Osaka General Medical Center. All images in the test dataset were double-checked at the same meeting before the final labels were assigned.

CO-RADS was reported to have a high sensitivity for detecting COVID-19 with a three or higher threshold setting^53^. Therefore, the images with a score of three or higher were given a positive label. The scores were provided to each slice and were independently judged without considering information from the previous or following slices. A series of one patient’s images were labeled as COVID-19 positive if only a single slice had a score of three or higher by a majority vote (Supplementary Sect. 6).

Images were labeled as COVID-19 negative if their case was confirmed as COVID-19 negative by the on-site physician through CT findings and other clinical data including PCR and follow-up examinations. All slices from confirmed negative cases were labeled as negative.

### Model

We developed two models: one determines whether a single CT image contains COVID-19 lesions (slice model), and the other determines whether a patient is infected by COVID-19 from a series of chest CT images (series model). Both models use deep learning to perform binary positive/negative classification. Although the input form differs, the network structure and output format are identical in both models. We adopted the ResNeSt-101 structure^44^ as the network backbone, followed by Global Average Pooling and a fully connected layer with an output dimension of two. Then, the output is subjected to a SoftMax operation such that the sum of the two values equals one, which results in an output value that can be interpreted as the confidence of the input being COVID-19 positive. The structure of the models was developed from scratch in PyTorch (version 1.7.0), referring to the ResNeSt paper^44^. Detailed structures are summarized in a text file using Torchinfo (version 1.6.1). This file is stored in the public repository (10.5281/zenodo.5835313), in which the model’s source code is also available and can be referred to for more details.

Figure 3b(i) shows the preparation of the inputs for the slice model. The input is a 3-channel image of shape (224, 224, 3) consisting of a target slice and slices before and after, arranged in the channel direction in order (before, target, after). In cases where the before and after slices do not exist, such as at the end of the lung field, the missing images were replaced with target slice images.

Figure 3b(ii) shows the preparation of the inputs for the series model. Twenty-seven images were selected at equal intervals from the pre-processed images in the series of the target case and divided into three groups of nine images. Each group was converted into 3 × 3 tiled images. The input to the series model is these tile images concatenated in the channel direction, whose shape is (672, 672, 3). This value of 27 images was designed as a necessary and sufficient value, given that the original images were 3–7 mm thick and found to provide better accuracy than other candidate values during our trials. For a series with less than 27 pre-processed images, the true-black images of shape (224, 224, 1) were inserted backward. The hconcat and vconcat modules of OpenCV (version 4.0.0.21) were used for image tiling. The details of the algorithm for selecting 27 slices from the entire slice of a series at equal intervals are described in the source code of the public repository (10.5281/zenodo.5835313).

### Training

The slice and series models are trained in the environment, as indicated in Table S6. This environment is built on a custom workstation (GPU: NVIDIA GeForce RTX 3090 24G, CPU: Intel Core i9-10980XE 18-core, memory: 128 GB RAM).

We used ImageNet pre-trained weights for the initial parameters of the slice model’s convolutional layers. We performed random rotation, random flip, and random erasing^54^ as data augmentation (Supplementary Sect. 8).

The model was trained with cross entropy loss between predictions and ground truth. The training epoch is 25 in total. The training batch size is 48. During training, we used a Stochastic Gradient Descent (SGD) optimizer at a momentum value of 0.9 and a weight decay coefficient of 0.0001. A learning rate was initialized at 0.01 and decays by a factor of 0.1 at 10th and 15th epoch. The model with the lowest validation loss was selected as the final model. The validation loss was the minimum at the 11th epoch.

We initialized the parameters of the series model’s all layers with those of the final slice model. This fine-tuning was expected to make it easier for the series model to acquire disease features, though it had less training data than the slice model. We performed random rotation, random flip, and random erasing^54^ as data augmentation (Supplementary Sect. 8). The model was trained with cross entropy loss. The training epoch is 50. The training batch size is 10. The optimizer, initial learning rate, and learning rate schedules are the same as those in the slice model.The model with the lowest validation loss was selected as the final model. The validation loss was the minimum at the 37th epoch.

### Evaluation

The following values were calculated to evaluate the performance of the final models in detecting COVID-19. The area under curves (AUC) is calculated from the receiver operating characteristics (ROC) curves for the validation dataset. Then the sensitivity, specificity, and accuracy are calculated from the ROC curves at the threshold point of 0.5, 95% sensitivity, and 95% specificity. The AUCs, sensitivity, specificity, and accuracy were calculated for the external test dataset using the same thresholds to evaluate extrapolation. The interpretability of the models was assessed through visualization using saliency maps^46^ to prove objectivity (Supplementary Sect. 9).

### Statistics

Agreement rates for the CO-RADS scores labeled by radiologists were calculated on a group basis using Fleiss' kappa statistics^55^. The mean values of the percent agreement and its 95% confidence interval were obtained for each group. For model evaluation, the bootstrap method^56^ with 2000 nonparametric nonhierarchical resampling was used to estimate 95% confidence intervals for AUC, sensitivity, specificity, and accuracy. Based on the processing times of the slice and series models measured in all cases of the test data for inference, their means and 95% confidence intervals were obtained. All statistical analyses were performed using Python packages including SciPy, NLTK, scikit-learn, and matplotlib (Supplementary Sect. 11).

### Supplementary Information

Supplementary Information.

## Data Availability

The datasets generated and analyzed during the current study are available in Zenodo (10.5281/zenodo.5835313).

## Code availability

The application software and its implementation code are also available in Zenodo (10.5281/zenodo.5835313).
