# Supplementary material for: “KAIZEN” method realizing implementation of deep-learning models for COVID-19 CT diagnosis in real world hospitals
Source: Sci Rep. 2024 Jan 19;14:1672. doi: 10.1038/s41598-024-52135-y (PMC10799049; doi:10.1038/s41598-024-52135-y)
Supplement: Supplementary file 1 — Supplementary Information. [file 41598_2024_52135_MOESM1_ESM.docx]

Supplementary Information

“KAIZEN” method realizing implementation of deep-learning models for COVID-19 CT diagnosis in real world hospitals

Corresponding author

Author name Naoki Okada, M.D.

Affiliations Osaka General Medical Center

Contact information wggdilp@gmail.com

Authors

Naoki Okada, M.D., Osaka General Medical Center

Yutaka Umemura, M.D., Ph.D., Osaka General Medical Center

Shoi Shi, Ph.D., University of Tsukuba

Shusuke Inoue, M.S., fcuro inc.

Shun Honda, M.S., fcuro inc.

Yohsuke Matsuzawa, Ph.D., Osaka Metropolitan University

Yuichiro Hirano, M.D., fcuro inc.

Ayano Kikuyama, M.D., Osaka General Medical Center

Miho Yamakawa, M.D., Osaka General Medical Center

Tomoko Gyobu, M.D., Ph.D., Osaka General Medical Center

Naohiro Hosomi, M.D., Osaka General Medical Center

Kensuke Minami, M.D., Osaka General Medical Center

Natsushiro Morita, M.D., Osaka General Medical Center

Atsushi Watanabe, M.D., Osaka General Medical Center

Hiroyuki Yamasaki, B.S., Shizuoka Saiseikai General Hospital

Kiyomitsu Fukaguchi, M.D., Shonan Kamakura General Hospital

Hiroki Maeyama, M.D., Ph.D., Tsuyama Chuo Hospital

Kaori Ito, M.D., Ph.D., Teikyo University

Ken Okamoto, M.D., Ph.D., Juntendo University Urayasu Hospital

Kouhei Harano, M.D., Showa University Hospital

Naohito Meguro, M.D., Tokyo Women's Medical University Hospital

Ryo Unita, M.D., National Hospital Organization Kyoto Medical Center

Shinichi Koshiba, M.D., Shizuoka Saiseikai General Hospital

Takuro Endo, M.D., Ph.D., International University of Health and Welfare, School of Medicine, Narita Hospital

Tomonori Yamamoto, M.D., Ph.D., Nara Prefecture General Medical Center

Tomoya Yamashita, M.D., Osaka City General Hospital

Toshikazu Shinba, M.D., Ph.D., Shizuoka Saiseikai General Hospital

Satoshi Fujimi, M.D, Ph.D., Osaka General Medical Center

Table of Contents

[Supplementary Section 1: Participating institutions 3](#_heading=h.30j0zll)

[Supplementary Section 2: KAIZEN Checklist 6](#_heading=h.2et92p0)

[Supplementary Section 3: Characteristics of CT equipment 7](#_heading=h.4d34og8)

[Supplementary Section 4: Anonymization 10](#_heading=h.2s8eyo1)

[Supplementary Section 5: Datasets and data partitions 10](#_heading=h.17dp8vu)

[Supplementary Section 6: Labeling 14](#_heading=h.3rdcrjn)

[Supplementary Section 7: Pre-processing methods 16](#_heading=h.35nkun2)

[Supplementary Section 8: Training 19](#_heading=h.44sinio)

[Training the slice model 19](#_heading=h.2jxsxqh)

[Training the series model 22](#_heading=h.2xcytpi)

[Supplementary Section 9: Saliency maps 25](#_heading=h.3as4poj)

[Supplementary Section 10: Inference environment and processing 28](#_heading=h.1pxezwc)

[Inference environment 28](#_heading=h.49x2ik5)

[Inference process 29](#_heading=h.2p2csry)

[Supplementary Section 11: Statistics method 31](#_heading=h.147n2zr)

[Agreement rate of labeling 31](#_heading=h.3o7alnk)

[Model evaluation 32](#_heading=h.ihv636)

[Processing times 32](#_heading=h.32hioqz)

[Supplementary Section 12: Detailed demographics of the dataset 32](#_heading=h.1hmsyys)

[Data demographics per institution 32](#_heading=h.41mghml)

[Patients’ disease demographics in the test dataset 34](#_heading=h.2grqrue)

[Supplementary Section 13: Classification performance 36](#_heading=h.vx1227)

[Supplementary Section 14: Failure analysis 43](#_heading=h.3fwokq0)

[Breakdown of the misclassified cases of the series model 43](#_heading=h.1v1yuxt)

[Analysis of the misclassification of the series model 43](#_heading=h.4f1mdlm)

[Breakdown of the misidentified cases of the slice model 46](#_heading=h.2u6wntf)

[Analysis of the misidentification of the slice model 51](#_heading=h.19c6y18)

[Supplementary Section 15: Performance of the inference process 53](#_heading=h.3tbugp1)

[Choice of batch size 53](#_heading=h.28h4qwu)

[Performance results 54](#_heading=h.nmf14n)

[Supplementary Section 16: Imaging diagnostic application software 55](#_heading=h.37m2jsg)

[References 58](#_heading=h.1mrcu09)

## Supplementary Section 1: Participating institutions

We collected only domestic cases to optimize the AI model developed for Japanese medical institutions. Twelve partner institutions, including university hospitals and emergency centers, with different geographical locations and sizes, were selected for data acquisition through the Japanese Association for Acute Medicine. This study was approved by the ethical committees of the core institution, the Osaka General Medical Center, and other subcontracting institutions (IRB: 2020-073). Informed consent was omitted after disclosing the study summary at all participating institutions.

Figure 1.1 shows that data is collected by personally sending encrypted hard disk drives (HDDs) as a safety control measure following the Japanese Personal Information Protection Law to prevent patient information from leaking onto external networks. The data stored on the HDDs include anonymized CT data (DICOM format) for each case and an Excel sheet containing the age, sex, and CT scan date, as shown in Figure 1.2. Each case is treated individually with an anonymous ID (e.g., ZA-001, ZA-002), and a table linking this ID to the patient’s name is maintained at each partner institution.


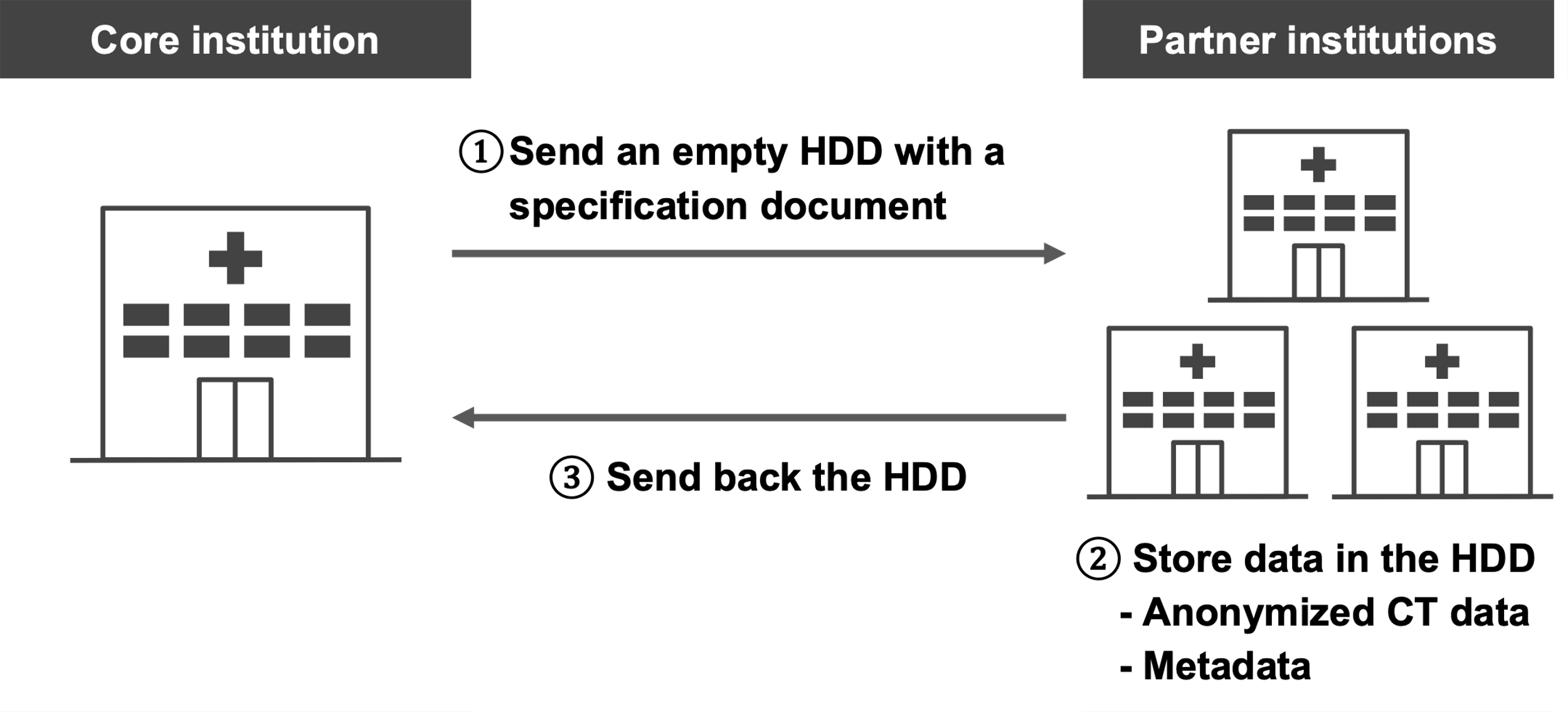


**Figure 1.1 Data collection method**: An empty hard disk drive (HDD) with a specification document was sent from the core institution to each partner institution. Anonymized CT data and accompanying metadata are stored in the HDD in encrypted form. This HDD is then sent to the core institution.


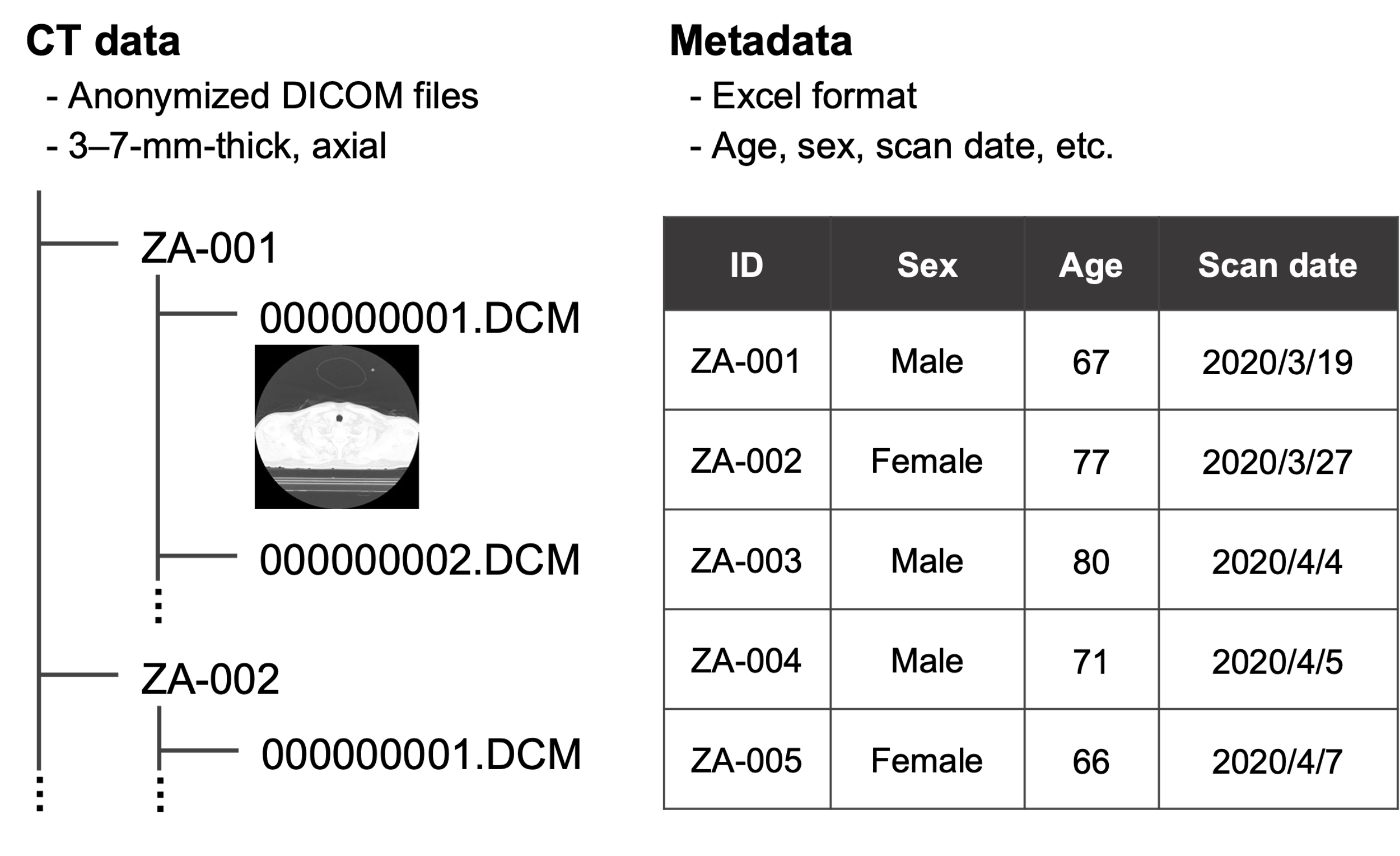


**Figure 1.2 Collected data per institution**: CT scans were performed in an anonymized DICOM format. The meta-analysis included anonymized ID, sex, age, and scan date for each CT scan.

Data is collected for three groups per institution: COVID-19, other lung diseases (OLD), and normal. The COVID-19 group includes all patients with positive PCR test results and treated as COVID-19-infected patients in clinical practice based on clinical judgment and diagnostic imaging by emergency physicians at each institution. Thus, patients with a negative initial PCR test who are strongly suspected and retested later and found to be infected are also included in this group. The OLD group includes patients with fever or respiratory symptoms and can be suspected of having COVID-19; however, their PCR test results are negative or are the cases before the COVID-19 pandemic. The normal group included patients who underwent chest CT scans, but no chest disease was detected by clinical judgment and diagnostic imaging. Further, CT data obtained at later follow-up periods were included in the collected data. Examination data not stored in the hospital's image storage system and missing examination data are excluded from the dataset. Tables 1.1 (a) and (b) show the number of patients, scans, slices, and collection periods for each institution based on the above settings. Table 1.1 (b) shows the data used for the external testing of the model. Although the data from the Osaka General Medical Center are used for both training/validation and external testing, they are completely divided by the period during which the data are acquired.

The number of patients required for the test data is derived by estimating the standard error of the area under the curve (AUC) from the number of positive and negative data^1^. Forty positive and 40 negative data points are estimated to be required for achieving a confidence interval (CI) < 0.1 when the target value of AUC is set to 0.95. Based on this estimation, the test data collection period is selected to meet the minimum of 40 cases in each group.

**Table 1.1 Collected data:** “Patients,” “Series,” and “Slices” represent the number of patients, number of series, and number of slices in total for each institution, respectively.

**(a) Collected data for model development (training/validation).**

|  | **Patients** | **Series** | **Slices** | **Collection Period** |
| --- | --- | --- | --- | --- |
| **Osaka General Medical Center** | | | | |
| COVID-19 | 134 | 253 | 130065 | 2020/03/19–2020/10/08 |
| OLD | 199 | 185 | 24630 | 2020/03/03–2020/11/13 |
| Normal | 152 | 149 | 24725 | 2017/04/01–2019/02/24 |
| **Teikyo University Hospital** | | | | |
| COVID-19 | 132 | 137 | 45628 | 2020/03/29–2020/12/09 |
| OLD | 1404 | 1515 | 658943 | 2020/03/01–2020/12/09 |
| **Shonan Kamakura General Hospital** | | | | |
| COVID-19 | 581 | 1275 | 532653 | 2020/05/23–2021/01/02 |
| **Juntendo University Urayasu Hospital** | | | | |
| COVID-19 | 37 | 60 | 25363 | 2020/04/03–2021/01/19 |
| **IUHW Narita Hospital** | | | | |
| COVID-19 | 107 | 298 | 120883 | 2020/03/31–2021/01/23 |
| **Tokyo Women's Medical University Hospital** | | | | |
| COVID-19 | 164 | 192 | 18232 | 2020/04/20–2020/12/09 |
| **Osaka City General Hospital** | | | | |
| COVID-19 | 143 | 219 | 87970 | 2020/02/17–2021/01/31 |
| **Nara Prefecture General Medical Center** | | | | |
| COVID-19 | 73 | 104 | 46172 | 2020/01/03–2020/12/24 |
| **Tsuyama Chuo Hospital** | | | | |
| COVID-19 | 28 | 50 | 3483 | 2020/09/29–2020/12/07 |
| OLD | 100 | 134 | 9724 | 2020/01/02–2020/03/25 |
| Normal | 100 | 105 | 7539 | 2020/01/01–2020/02/12 |
| **Showa University Hospital** | | | | |
| COVID-19 | 76 | 95 | 55504 | 2020/03/27–2020/12/16 |
| OLD | 188 | 228 | 152148 | 2020/04/06–2020/11/30 |
| **Shizuoka Saiseikai General Hospital** | | | | |
| OLD | 500 | 500 | 167035 | 2019/09/02–2020/12/11 |
| Normal | 500 | 500 | 197550 | 2020/03/01–2020/11/30 |

**(b): Collected data for model testing (test).**

|  | **Patients** | **Series** | **Slices** | **Collection Period** |
| --- | --- | --- | --- | --- |
| **Osaka General Medical Center** | | | | |
| COVID-19 | 123 | 110 | 7911 | 2020/12/01–2021/01/31 |
| OLD | 34 | 32 | 2348 | 2020/12/03–2021/01/27 |
| Normal | 7 | 7 | 505 | 2020/12/10–2021/01/27 |
| **National Hospital Organization Kyoto Medical Center** | | | | |
| COVID-19 | 46 | 57 | 13896 | 2020/04/09–2020/12/14 |
| OLD | 182 | 180 | 40097 | 2020/02/16–2020/12/09 |
| Normal | 146 | 146 | 27196 | 2020/04/17–2020/12/09 |

## Supplementary Section 2: KAIZEN Checklist

Checklist for Artificial Intelligence in Medical Imaging (CLAIM) is a leading guideline for developing diagnostic imaging AI models presented by Mongan et al.^2^. The radiomics quality score (RQS) presented by Lambin et al.^3^ was suggested for evaluating models using machine learning methods. They focus on data design, annotation design, model design, model training design, and model evaluation design.

In addition, inference should be considered to develop diagnostic AI models that can be utilized in clinical practice^4^. It is essential for such AI models to output (infer) results in a short time without obstructing any medical acts, even with limits to the device’s computing environment (whether local or in the cloud) used inside hospitals. We developed COVID-19 CT diagnostic AI models based on the KAIZEN checklist of 45 items. Table 2.1 presents a list of three additional inference items that we propose to add to the 42 items in the CLAIM^5–8^.

Item 43 presents the essential points of the detailed inference process that researchers must describe, including data loading, data formatting, batch size setting, and model execution. For data loading, researchers must deal with any of the characteristics of the data in each clinical site where the model is to be applied. Some data formats, such as NIFTI, Analyze, and Minc, are easy to manage for research purposes and are used during model development^9^. Nonetheless, the inference process should accept data in DICOM format because almost all clinical sites to which we apply the model manage data in the DICOM format^10^. Further, researchers must describe the data formatting method used to apply the loaded file to pre-processing. If the pre-processing algorithm differs from that used for training, it should also be described. Researchers must also describe inference settings optimized for the hardware environment’s specifications (e.g., CPU, memory) wherein the inference process is executed. Sze et al.^5^ pointed out that while deep neural networks deliver state-of-the-art accuracy on many AI tasks, they incur the cost of high computational complexity. Therefore, the batch size at the model runtime and the number of models launched simultaneously must be optimized for the inference environment. Researchers must clarify the entire model execution process flow from loading the data to the result. This is desirable for using a flowchart or other graphical representation.

Item 44 presents the essential points for describing the execution environment. Researchers must describe the hardware specifications, software libraries, frameworks, and packages used for their model inference.

Item 45 presents the inference performance index’s essential points, including inference speed or time and memory consumption during inference. This is necessary because inference processes, including AI models, have variable performance in inference speed and memory consumption based on the hardware device used to implement them^6^. It is desirable to conduct performance comparisons of measures for optimizing the inference processing for the implementation environment to demonstrate their effectiveness.

**Table 2.1 Proposed items 43-45 added to the CLAIM checklist:** We propose adding these items to the CLAIM checklist and calling it the “KAIZEN checklist.”

| **Section/Topic** | **No.** | **Item** |
| --- | --- | --- |
| **INFERENCE** | | |
|  | 43 | Detailed description of the inference process; data loading, data formatting, batch size setting, and model execution |
|  | 44 | Hardware specification, software libraries, frameworks, and packages |
|  | 45 | Evaluation of inference performance including inference speed or time and memory consumption |

## Supplementary Section 3: Characteristics of CT equipment

The details of each institution’s CT equipment, PACS, and anonymization software are listed in Tables 3.1. As indicated in Table 3.1, the data collected are not biased toward any CT imaging equipment or the imaging characteristics of any manufacturer. The PACS and anonymization software are also commonly distributed, and we confirmed that no extra processing is performed on the images during the data acquisition process.

**Table 3.1 Details of each institution’s CT equipment, PACS, and anonymization software.**

| **CT scanner** |  |  | **PACS** | **Anonymization Software** |
| --- | --- | --- | --- | --- |
| **Osaka General Medical Center** | | | | |
| Canon Medical Systems,  Aquilion CX |  |  | FUJIFILM Medical,  SYNAPSE | AMIN, ziostation2 |
| **Teikyo University Hospital** | | | | |
| Canon Medical Systems,  Aquilion 64 | Canon Medical Systems,  Aquilion ONE | Canon Medical Systems,  Aquilion LB | PSP,  EV Insite net | PSP,  EV Insite net |
| Siemens Healthineers,  SOMATOM Definition Flash | GE Healthcare,  LightSpeed VCT XT |  |  |  |
| **Shonan Kamakura General Hospital** | | | | |
| Siemens Healthineers, SOMATOM Scope | Siemens Healthineers,  SOMATOM Sensation Cardiac 64 |  | FUJIFILM Medical,  ShadeQuest/Serv | FUJIFILM Medical,  ShadeQuest/View R |
| **Juntendo University Urayasu Hospital** | | | | |
| Canon Medical Systems,  Aquilion Lightning / Helios Edition | Canon Medical Systems,  Aquilion ONE GENESIS Edition | Siemens Healthineers,  SOMATOM Definition Flash | FUJIFILM Medical,  SYNAPSE | CODONICS,  CODONICS Virtua |
| **IUHW Narita Hospital** | | | | |
| Canon Medical Systems,  AquilionPrimeSP |  |  | GE Healthcare,  Centricity | Array,  Array AOC |
| **Tokyo Women's Medical University Hospital** | | | | |
| Canon Medical Systems, Aquilion 64 | Canon Medical Systems,  Aquilion 16 | Canon Medical Systems,  Aquilion ONE | FUJIFILM Medical,  ShadeQuest/Serv | FUJIFILM Medical,  ShadeQuest/View R |
| Canon Medical Systems,  Aquilion ONE GENESIS Edition | GE Healthcare, Revolution Maxima |  |  |  |
| **Osaka City General Hospital** | | | | |
| Canon Medical Systems,  Aquilion PRIME |  |  | FUJIFILM Medical,  SYNAPSE | Array,  Array AOC |
| **Nara Prefecture General Medical Center** | | | | |
| Canon Medical Systems,  Aquilion Prime SP |  |  | FUJIFILM Medical,  SYNAPSE | Carina system,  ADMENIC client |
| **Tsuyama Chuo Hospital** | | | | |
| GE Healthcare,  LightSpeed VCT Vision | GE Healthcare,  BlightSpeed ELITE |  | PSP,  EV Insite net | PSP,  EV Insite net |
| **Showa University Hospital** | | | | |
| GE Healthcare,  LightSpeed VCT Vision |  |  | FUJIFILM Medical,  SYNAPSE | FUJIFILM Medical,  SYNAPSE |
| **Shizuoka Saiseikai General Hospital** | | | | |
| GE Healthcare,  Discovery 750 HD | GE Healthcare,  Revolution CT |  | GE Healthcare,  Centricity | Array,  Array AOC |
| **National Hospital Organization Kyoto Medical Center** | | | | |
| Canon Medical Systems,  Aquilion Prime SP |  |  | GE Healthcare,  Centricity | Array,  Array AOC |

## Supplementary Section 4: Anonymization

Anonymization is performed on the collected patients based on the Japanese Personal Information Protection Law. Individual patient information is anonymized when partner institutions store data on HDDs, as described in Supplementary Section 1. The CT data are anonymized by omitting personally identifiable names, dates of birth, and other specific information from the DICOM headers. Further, we excluded head CT slices from the data if included to ensure that the identification of the individual’s face cannot be performed. We confirmed that anonymization is completed after collecting the data at the core institution of the Osaka General Medical Center. In the confirmation process at the core institution, Pydicom (version 2.1.2) automatically confirmed that the names and dates of birth were not included in the DICOM headers. The exclusion of the head CT slices is visually confirmed in all cases.

## Supplementary Section 5: Datasets and data partitions

We excluded the following cases from the collected data:

1. Cases with missing or corrupted CT data in lung fields.
2. Cases without complete lung fields in the CT data.
3. Cases with motion artifacts.
4. Cases with other artifacts (e.g., metal artifacts overlapping the lung fields and intrathoracic devices).
5. PCR-positive cases without any COVID-19 findings by radiologists.
6. Cases with patients younger than 18 years old.

Patients were excluded in this order. Step (1) describes cases included in the Excel data; however, CT scans were missing or corrupted during the collection or anonymization process. We selected exclusion targets in steps (2)–(4) by visually checking all captured data in the same environment and with the same workers as for the annotations shown in Supplementary Section 6. Figure 5.1 shows examples of cases excluded in steps (3) and (4). Step (5) was based on the results of the annotation of patients in the COVID-19 group shown in Supplementary Section 6. Cases with no significant COVID-19 lesions in the lung fields were excluded in this step. We used Excel data described in Supplementary Section 1 to select cases to be excluded in step (6).

After exclusion, we split the data collected for training/validation into training and validation datasets. The training dataset was used to train the models’ parameters, and the validation dataset was used for the termination conditions of the training and selection of the final models. Splitting was performed using the following steps as a holdout method:

- Assigning a unique ID to each case
- Generating an array consisting of the IDs of all cases
- Shuffling the array randomly
- Defining the first 80% of the array as the training dataset and the remaining data as the validation dataset

Splitting was performed using Python 3.7.4, and the random module of the standard library was used for shuffling the array. This splitting was performed on a per-patient basis, and the CT images of the same patient were not included in either the training or validation datasets.

The data exclusion and splitting results described above are presented in Tables 5.1 (a) and (b). Table 5.1 (a) shows the training and validation data used to develop the model, and Table 5.1 (b) shows the data used to test the model. For each group, the total number of samples, the number of samples excluded in steps (1)–(6) above, and the number of samples after exclusion are presented in the tables.


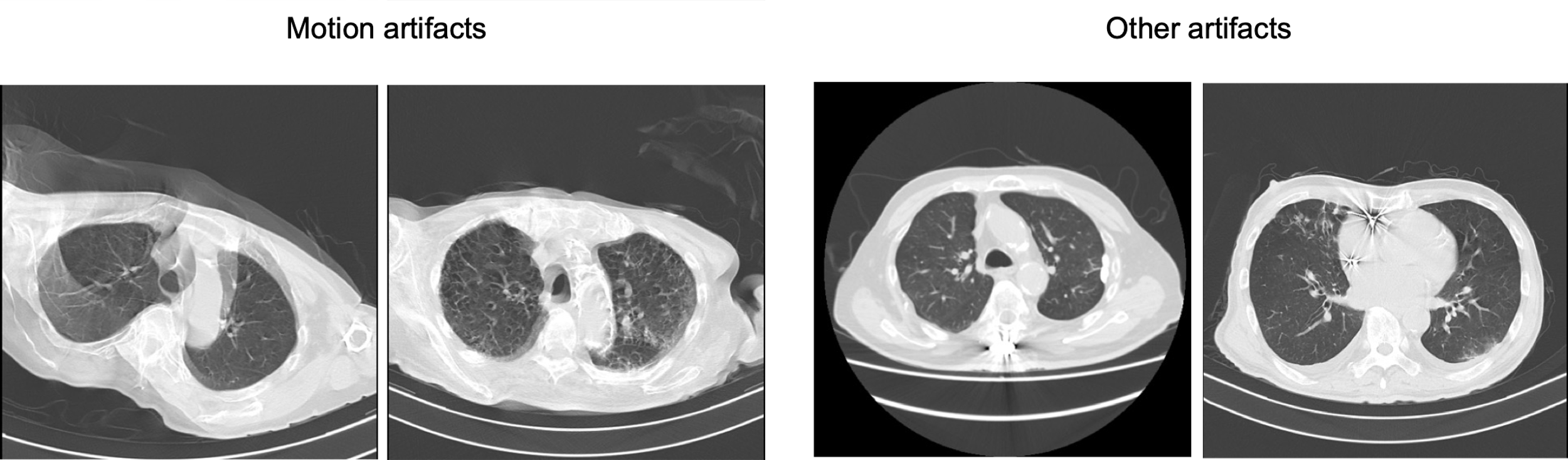


**Figure 5.1 Examples of excluded cases:** The two images on the left are slices with motion artifacts, such as body movement. The two right images are examples of slices with other artifacts, such as metal artifacts, which can affect the visibility of the lung fields.

**Table 5.1 Data exclusion and splitting:** “Collected” and “Excluded” represent the number of collected samples in total and the numbers of samples excluded in steps (1) to (6) described in the supplemental text, respectively. Further, “Included” represents the number of samples finally included. The “training data (slices),” “validation data (slices),” and “test data (slices)” represent the number of slices used. The “training data (series),” “validation data (series),” and “test data (series)” represent the number of series used.

**(a): Data exclusion and splitting for model development (training/validation).**

|  | **Collected** | **Excluded** | **Included** | **Slice model** | | **Series model** | |
| --- | --- | --- | --- | --- | --- | --- | --- |
|  |  |  |  | **Training data**  **(slices)** | **Validation data**  **(slices)** | **Training data**  **(series)** | **Validation data**  **(series)** |
| **Osaka General Medical Center** | | | | | | | |
| COVID-19 | 134 | (1) 2 (2) 0 (3) 8 (4) 2 (5) 5 (6) 0 | 117 | 3398 | 912 | 180 | 34 |
| OLD | 199 | (1) 14 (2) 0 (3) 61 (4) 2 (5) 0 (6) 0 | 122 | 4398 | 1035 | 104 | 18 |
| Normal | 152 | (1) 3 (2) 0 (3) 31 (4) 9 (5) 0 (6) 9 | 100 | 3749 | 982 | 77 | 23 |
| **Teikyo University Hospital** | | | | | | | |
| COVID-19 | 132 | (1) 0 (2) 0 (3) 2 (4) 2 (5) 44 (6) 2 | 82 | 1480 | 287 | 66 | 18 |
| OLD | 1404 | (1) 17 (2) 19 (3) 285 (4) 34 (5) 0 (6) 11 | 1038 | 42130 | 10030 | 881 | 219 |
| **Shonan Kamakura General Hospital** | | | | | | | |
| COVID-19 | 581 | (1) 0 (1) 5 (2) 102 (3) 5 (4) 115 (5) 0 | 354 | 7232 | 1825 | 558 | 159 |
| **Juntendo University Urayasu Hospital** | | | | | | | |
| COVID-19 | 37 | (1) 1 (2) 0 (3) 0 (4) 4 (5) 0 (6) 30 | 30 | 790 | 71 | 43 | 5 |
| **IUHW Narita Hospital** | | | | | | | |
| COVID-19 | 107 | (1) 0 (2) 0 (3) 1 (4) 1 (5) 28 (6) 77 | 77 | 1615 | 586 | 188 | 50 |
| **Tokyo Women's Medical University Hospital** | | | | | | | |
| COVID-19 | 164 | (1) 0 (2) 2 (3) 6 (4) 41 (5) 0 | 115 | 2314 | 589 | 104 | 24 |
| **Osaka City General Hospital** | | | | | | | |
| COVID-19 | 143 | (1) 21 (2) 11 (3) 5 (4) 7 (5) 0 (6) 98 | 98 | 4176 | 539 | 142 | 24 |
| **Nara Prefecture General Medical Center** | | | | | | | |
| COVID-19 | 73 | (1) 2 (2) 1 (3) 3 (4) 4 (5) 2 (6) 0 | 61 | 1935 | 362 | 63 | 20 |
| **Tsuyama Chuo Hospital** | | | | | | | |
| COVID-19 | 28 | (1) 0 (2) 0 (3) 1 (4) 1 (5) 12 (6) 0 | 14 | 262 | 116 | 23 | 2 |
| OLD | 100 | (1) 0 (2) 0 (3) 4 (4) 0 (5) 0 (6) 84 | 84 | 3110 | 657 | 78 | 30 |
| Normal | 100 | (1) 0 (2) 0 (3) 9 (4) 8 (5) 0 (6) 0 | 83 | 2841 | 871 | 76 | 11 |
| **Showa University Hospital** | | | | | | | |
| COVID-19 | 76 | (1) 6 (2) 5 (3) 2 (4) 3 (5) 23 (6) 0 | 37 | 671 | 185 | 33 | 11 |
| OLD | 188 | (1) 6 (2) 29 (3) 8 (4) 7 (5) 0 (6) 0 | 138 | 5249 | 1111 | 125 | 33 |
| **Shizuoka Saiseikai General Hospital** | | | | | | | |
| OLD | 500 | (1) 0 (2) 1 (3) 83 (4) 22 (5) 0 (6) 0 | 394 | 29215 | 8569 | 310 | 84 |
| Normal | 500 | (1) 0 (2) 0 (3) 20 (4) 5 (5) 0 (6) 5 | 470 | 38444 | 10567 | 375 | 95 |

**(b): Data exclusion for model testing (test).**

|  | **Collected** | **Excluded** | **Included** | **Slice model** | **Series model** |
| --- | --- | --- | --- | --- | --- |
|  |  |  |  | **Test data (slices)** | **Test data (series)** |
| **Osaka General Medical Center** | | | | | |
| COVID-19 | 123 | (1)13 (2) 0 (3) 8 (4) 5 (5) 4 (6) 0 | 93 | 4045 | 93 |
| OLD | 34 | (1) 2 (2) 0 (3) 8 (4) 1 (5) 0 (6) 0 | 23 | 980 | 23 |
| Normal | 7 | (1) 0 (2) 0 (3) 0 (4) 0 (5) 0 (6) 0 | 7 | 298 | 7 |
| **National Hospital Organization Kyoto Medical Center** | | | | | |
| COVID-19 | 46 | (1) 1 (2) 0 (3) 7 (4) 2 (5) 9 (6) 0 | 27 | 1249 | 27 |
| OLD | 179 | (1) 3 (2) 3 (3) 34 (4) 8 (5) 0 (6) 1 | 133 | 5863 | 133 |
| Normal | 146 | (1) 0 (2) 1 (3) 12 (4) 6 (5) 0 (6) 4 | 123 | 6843 | 123 |

## Supplementary Section 6: Labeling

We labeled a slice image as COVID-19 positive if the case was PCR-positive and found to have some CT findings of COVID-19 by radiologists’ majority vote. All CT images of PCR-positive cases were grouped by the institution where they were taken and further subdivided internally for institutions, Tables 6.1 (a) and (b). These were scored independently of each other into five categories corresponding to the findings presented in the CO-RADS^11^ by eight radiologists who did not directly treat the patients and were given only these images. Category 6 was excluded because it was defined as PCR-positive.

**Table 6.1 Labeling agreement rates**: Each row represents a sub-group. “Patients” represent the number of patients in the subgroup, the average agreement rate in the subgroup with its 95% confidence interval, interpretation of the average agreement rate (“Poor” for values under 0, “Slight” for values from 0.00 to 0.20, “Fair” for values from 0.21 to 0.40, “Moderate” for values from 0.41 to 0.60, “Substantial” for values from 0.61 to 0.80, and “Almost Perfect” for values from 0.81 to 1.00), respectively.

**(a): Labeling agreement rates (training/validation).**

| **Institution** | **Patients** | **Agreement rate [95% CI]** | **Interpretation** |
| --- | --- | --- | --- |
| Osaka General Medical Center | 132 | 0.760 [0.718–0.803] | Substantial |
| Teikyo University Hospital | 132 | 0.559 [0.504–0.615] | Moderate |
| Shonan Kamakura General Hospital | 100 | 0.513 [0.370–0.656] | Moderate |
|  | 100 | 0.561 [0.485–0.637] | Moderate |
|  | 100 | 0.659 [0.593–0.725] | Substantial |
|  | 100 | 0.650 [0.587–0.714] | Substantial |
|  | 100 | 0.709 [0.649–0.769] | Substantial |
|  | 81 | 0.753 [0.700–0.806] | Substantial |
| Juntendo University Urayasu Hospital | 36 | 0.602 [0.524–0.679] | Moderate |
| IUHW Narita Hospital | 107 | 0.781 [0.732–0.831] | Substantial |
| Tokyo Women's Medical University Hospital | 164 | 0.645 [0.583–0.706] | Substantial |
| Osaka City General Hospital | 122 | 0.752 [0.690–0.813] | Substantial |
| Nara Prefecture General Medical Center | 71 | 0.672 [0.623–0.720] | Substantial |
| Tsuyama Chuo Hospital | 28 | 0.735 [0.660–0.809] | Substantial |
| Showa University Hospital | 70 | 0.694 [0.609–0.779] | Substantial |
| TOTAL | 1443 | 0.675 [0.658–0.691] | Substantial |

**(b): Labeling agreement rates (test).**

| **Institution** | **Patients** | **Agreement rate [95% CI]** | **Interpretation** |
| --- | --- | --- | --- |
| Osaka General Medical Center | 98 | 0.617 [0.518–0.715] | Substantial |
|  | 12 | 0.432 [0.374–0.490] | Moderate |
| National Hospital Organization Kyoto Medical Center | 45 | 0.525 [0.300–0.750] | Moderate |
| TOTAL | 155 | 0.493 [0.443–0.543] | Moderate |

The radiologists were trained before labeling to eliminate variations. They were instructed to refer to the manual on CO-RADS and label dozens of cases as a trial according to the manual. We published the manual on Zenodo (<https://doi.org/10.5281/zenodo.5835313>).

Each radiologist was provided a blank Excel file with case numbers for which they were responsible, and all of them were instructed to fill in the following items:

- Name of the radiologist
- CO-RADS score per slice
- Comorbidities identified
- Range of slices that were difficult to label and reason
- Other notes

We checked for deficiencies and requested they correct the data again if any corrections were necessary after the radiologists in charge completed the work. The results were exported to CSV files and converted to case-by-case Yaml files for ease of further analysis and use. This conversion was performed mechanically with Python 3.7.4 using the standard library csv module and PyYAML (version 6.0).

The images of the training and validation dataset in which the majority vote failed or for which at least one radiologist noted it was challenging to diagnose were double-checked and labelled at the radiologist conferences (where at least three board-certified radiologists with more than ten years of clinical experience attended) at the Osaka General Medical Center (Figure 6.1). All images in the test dataset were double-checked at the same conference before the final labels were assigned.


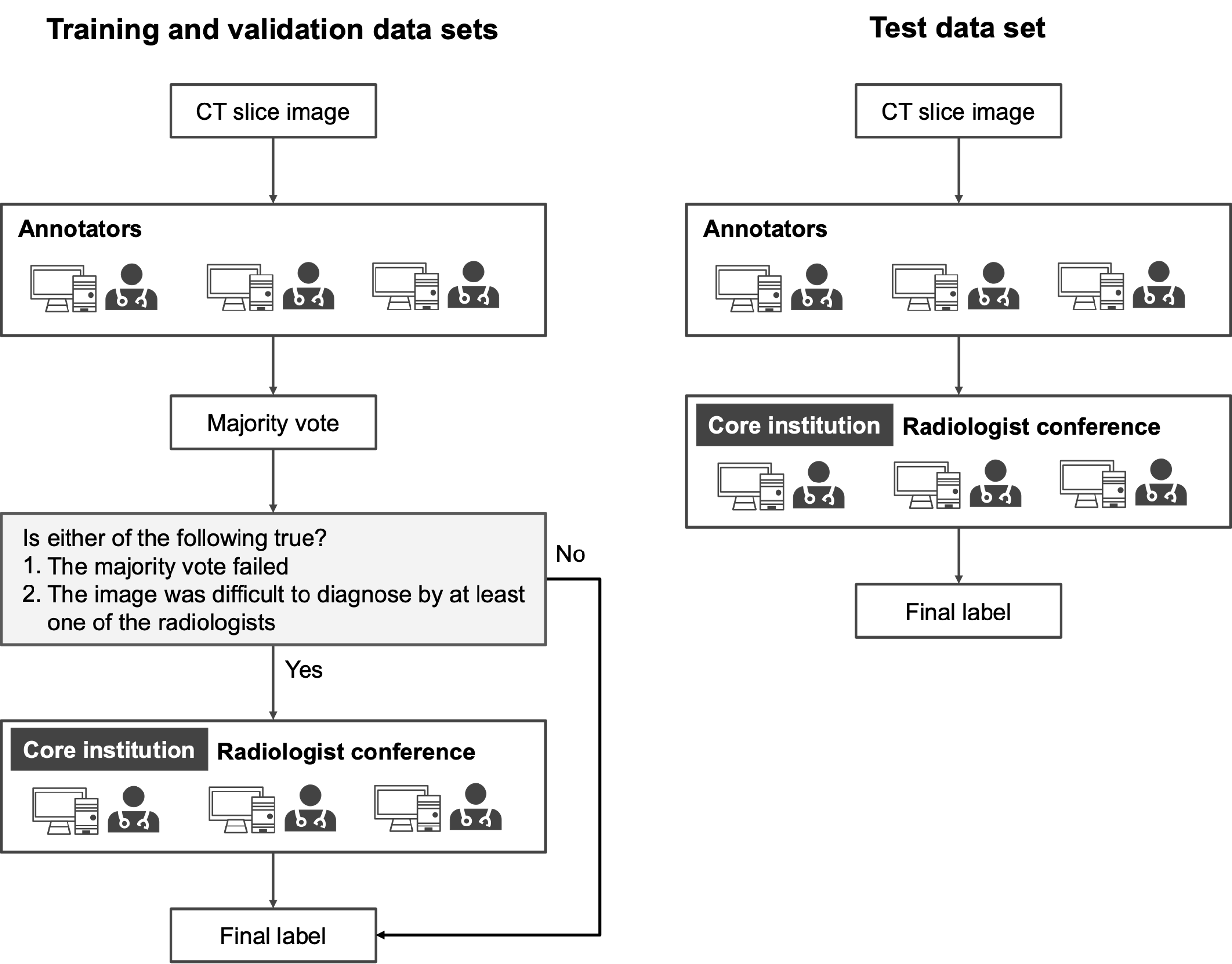


**Figure 6.1 Annotation scheme:** Three radiologists independently assigned scores for each slice image, and the majority vote results were adopted. If the majority vote failed or if the image was difficult to diagnose by at least one of the radiologists, it was further discussed in radiologist conferences at the Osaka General Medical Center. All images in the test dataset are double-checked at the same conference before the final labels are assigned.

CO-RADS had a high sensitivity for detecting COVID-19 with a threshold setting of three or higher^11^; therefore, images with a score of three or higher were given a positive label. Scores were provided for each slice image, and the images were judged independently without considering the information from the previous or following slices. A positive label was assigned to a group of images for the entire case if the majority vote provided even one slice image with a score of three or higher.

The collected CT data were divided into subgroups of approximately 100 patients, and three different radiologists labeled each subgroup. Tables 6.1 (a) and 6.1 (b) present the number of labeled patients and agreement rates of the three radiologists for each subgroup. Table 6.1 (a) describes the results for the training and validation datasets, and Table 6.1 (b) presents the results for the test dataset.

The agreement rates shown in the tables were calculated as follows:

1. Slices with a CO-RADS score of three or higher are considered positive, otherwise negative.
2. Calculated Fleiss’ kappa between the three radiologists in charge of each patient.
3. Calculated the average of Fleiss’ kappa across all the patients in a subgroup.

See Supplementary Section 11 for details on how the values of Fleiss’ kappa were calculated and their interpretation.

Table 6.1 (a) shows that the agreement rates for the training and validation data ranged from 0.513 (interpretation [IP], moderate) to 0.781 (IP, substantial) with an average of 0.675 (95% confidence interval [CI], 0.658–0.691; IP, substantial). As shown in Table 6.1 (b), the agreement rates on the test data ranged from 0.432 (IP, moderate) to 0.617 (IP, moderate), with an average of 0.493 (95% CI: 0.443–0.543; IP, moderate).

## Supplementary Section 7: Pre-processing methods

The lungmask package (<https://github.com/JoHof/lungmask>) filters the slices containing lung fields from the collected data and standardizes the input data. This package is open-source software (OSS) for lung field detection developed by Hofmanninger et al. and published in Apache License 2.0^12^. The OSS contains a pre-trained U-Net model based on CT data of non-COVID-19 cases collected at the Medical University of Vienna and chest CT data of COVID-19 cases published by MedSeg. It can perform 3-class semantic segmentation (left lung, right lung, and background) with CT slices as input. We forked the lungmask repository to fix the source code and model parameters and published it (<https://github.com/fcuro-inc/lungmask>) under the same license as the original repository.

Figure 7.1 shows the pre-processing method in detail. The lungmask module takes an array of shapes (512, 512, 1) as input, which should be part of the pixel array tag in a DICOM file and represents the CT values. We performed lung field detection on all horizontal slices (3–7 mm thick) on the collected CT scans. From the results, we filtered out slice images without lung fields and cropped and resized slice images with lung fields so that only lung fields remained.

A slice image was filtered out if any of the following conditions were met:

- Both right and left lungs are not detected.
- The minimum bounding rectangle (MBR) of the detected right and left lungs does not intersect with the vertical center line of the image.
- The height and width of the MBR of the detected right and left lungs are less than one-fifth of those of the image.

The following image transformations were applied to all slice images that were not filtered out:

- Window the image with the pulmonary window setting (width, 1500 HU; window center, –700 HU). The windowing results are stored in 32-bit floating-point variables between 0 and 1.
- Smooth the image with a 3 × 3 median filter.
- The region outside the lungs is masked by replacing the values in the pixels recognized as the background by the lungmask with ones.
- The image is cropped using the MBR of the detected right and left lungs using a lungmask with 5% horizontal and vertical margins.
- Resize the cropped image to 224 × 224.


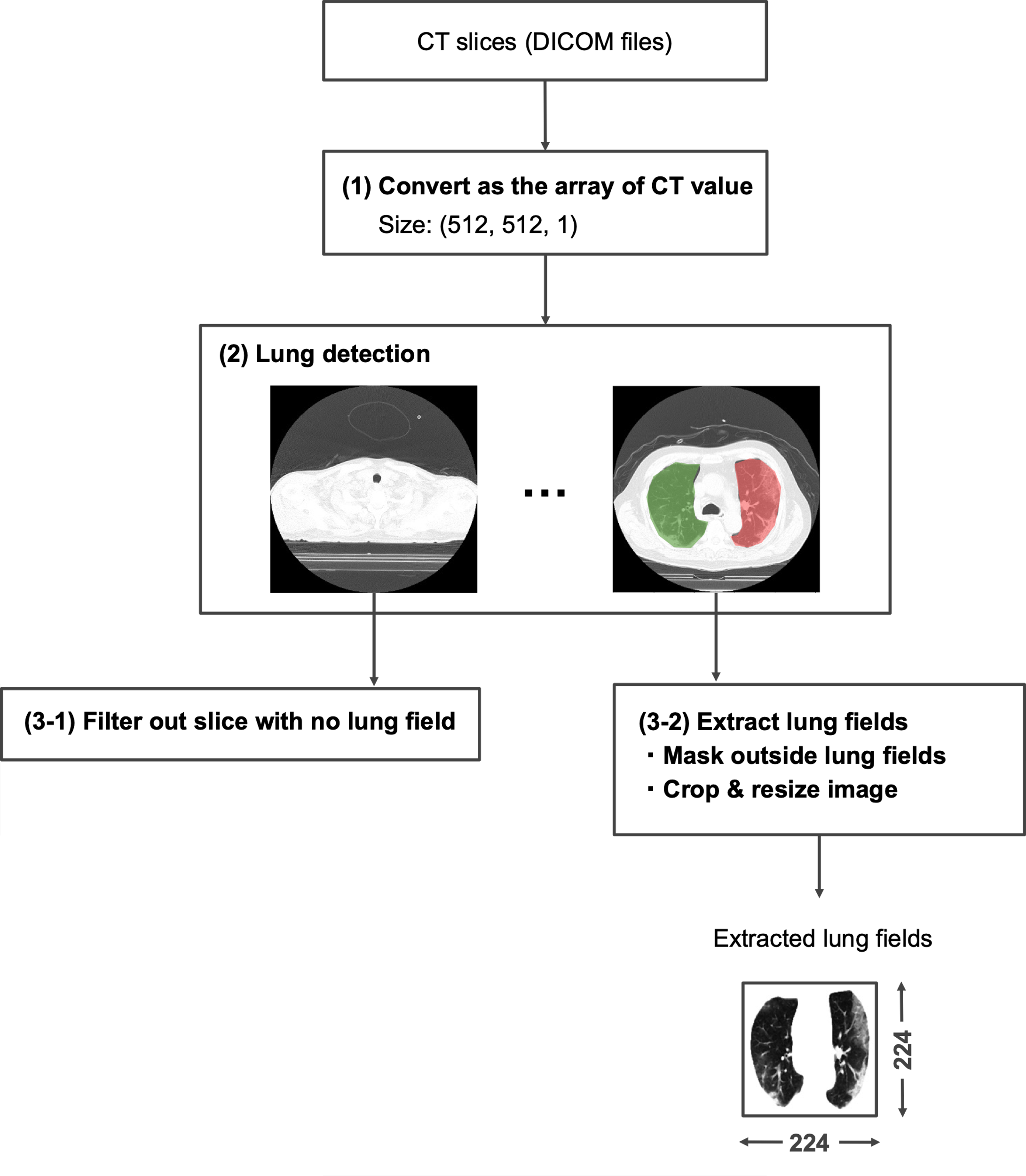


**Figure 7.1 Flow of pre-processing:** Each CT slice is first fed to a lung mask for lung field detection. Slices without lung fields are filtered out. Subsequently, the slices are cropped and resized so that only the lung fields remain.

In the above process, smoothing standardizes the noise characteristics of each imaging device and the image characteristics after reconstruction. Masking reduces the risk of bias of overfitting the model to regions outside the lung fields. For resizing the image to 224, a value widely used in general image recognition, we employed bicubic interpolation to minimize degradation. We selected this value because the original size of the slice image was 512 × 512, from which only the lung fields were extracted, and because of the memory constraints of the GPU. The details of this filtering and image transformation pre-processing and the library used are provided in the source code of our public repository (<https://doi.org/10.5281/zenodo.5835313>).

The pre-processing results are shown in Figure 7.2. The figure includes data from all institutions in which the data are collected. The upper part of the figure shows the original slice images, and the lower part shows the corresponding pre-processed image for each slice.

Figure 7.2 indicates that the original slice images have variations in the position and magnification of the lung fields, the presence or absence of arms, and the chest parts other than the lung fields based on the institution and patient. If these features are trained as they are, there is a risk of bias in which the model overfits the above features other than the lung fields; however, these differences are absorbed in the pre-processed images, and the risk of bias is eliminated.


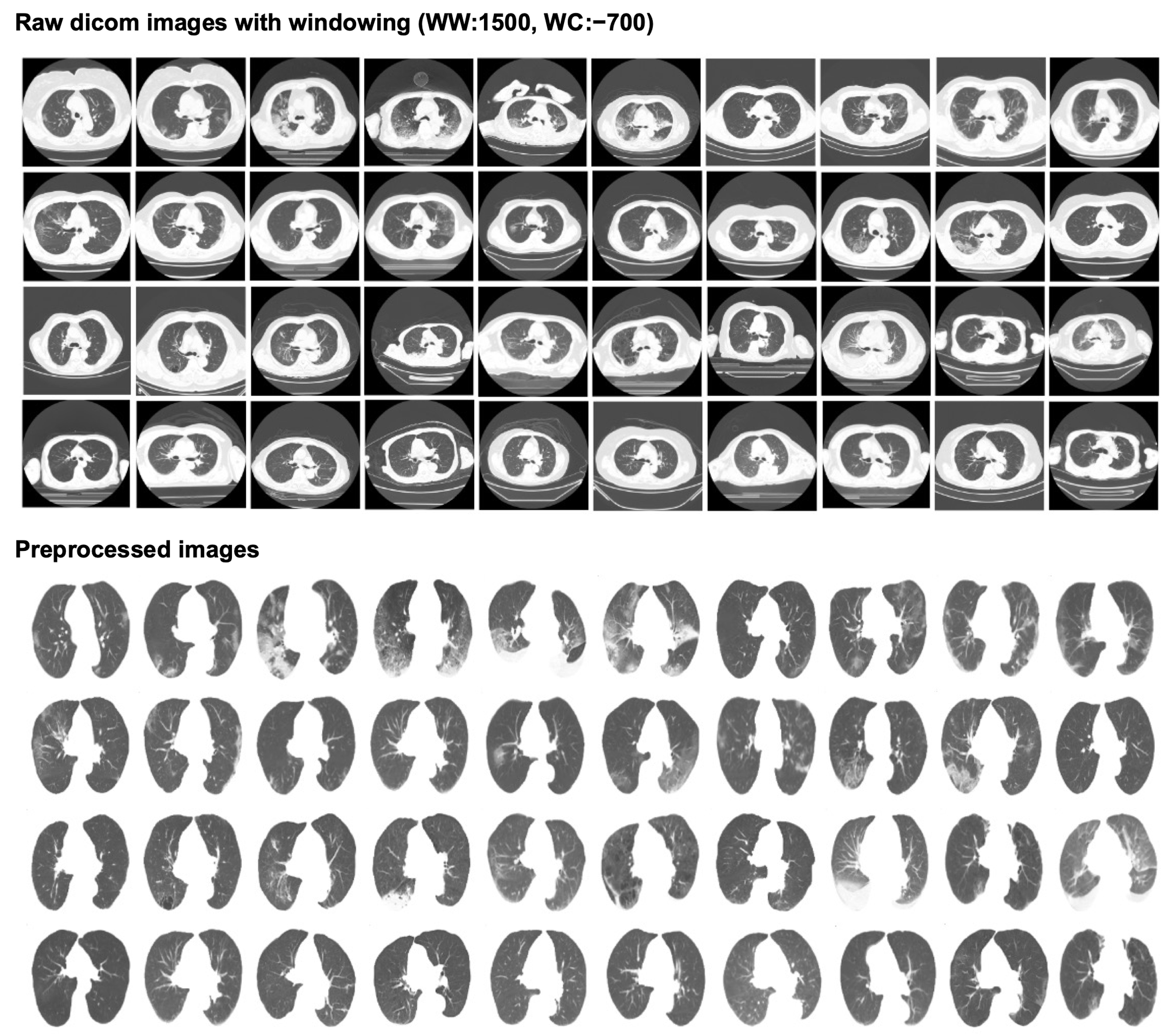


**Figure 7.2 Examples of pre-processing results:** The upper half of the figure shows the original slices with a window width of 1500 HU and a window center of –700 HU. The corresponding pre-processed images are shown in the lower half of the figure.

## Supplementary Section 8: Training

The slice and series models described in the Methods section are trained in the environment, as indicated in Table 8.1. This environment is built on a custom workstation (GPU: NVIDIA GeForce RTX 3090 24G, CPU: Intel Core i9-10980XE 18-core, memory: 128 GB RAM).

**Table 8.1 Training environment**

| **Training environment** | |
| --- | --- |
| OS | Ubuntu 20.04.1 LTS |
| GPU | Nvidia GeForce RTX 3090 24G |
| NVIDIA libraries | CUDA (version 11.0), cuDNN (version 8.0.4) |
| Python version | 3.7.4 |
| Deep-learning modules | Pytorch (version 1.7.0), Tensorboard (version 2.2.0) |
| DICOM processing modules | Pydicom (version 2.1.2), gdcm (version 1.1) |

### Training the slice model

The training dataset for the slice model consisted of 23,873 images of COVID-19, 84,102 images of OLD, and 45,034 normal images. The validation dataset consisted of 5,472 images of COVID-19, 21,402 images of OLD, and 12,420 normal images. The breakdown of the data by the institution is presented in Table 8.2. For the slice model, only the CT series taken for the first time for each patient was used, excluding those taken at the follow-up. COVID-19 images were treated as positive, whereas OLD and normal images were treated as negative. Thus, there were 23,873 positive and 129,136 negative images in the training dataset and 5,472 positive and 33,822 negative images in the validation dataset.

We used ImageNet pre-trained weights for the initial parameters of the slice model’s convolutional layers. The final fully connected layer weights were randomly initialized using the uniform distribution U(-$\sqrt{k}, \sqrt{k}$) ($k=1/2048$). A random range was designed because the number of units in the fully connected layer was 2048. Training hyperparameters were determined as follows;

- Optimizer selected from Momentum SGD^13^, Adam^14^, and AdamW^15^
- Mini-batch size selected from [24, 48]
- Weight decay selected from [0.00001, 0.00005, 0.0001, 0.0005, 0.001, 0.005, 0.001]
- Initial learning rate selected from [0.1, 0.05, 0.01, 0.005, 0.001]

The best result was achieved using the settings listed in Table 8.3.

During training, the loss and accuracy of the validation data were calculated for each epoch, and a checkpoint was saved for each epoch. We did not freeze the network parameters, which include ImageNet pre-trained parameters, and we updated the parameters for all the layers.

The following three transformations were performed as data augmentation for each iteration during training.

- Rotate the image (random rotation)
- Flip the image left and right (random flip)
- Convert a portion of the image into random noise (random erasing^16^)

**Table 8.2 Training and validation data for the slice model:** “Patients,” “Series,” and “Slices” represent the number of patients included, the number of series used, and the number of slices used in total. The number of series was always equal to the number of patients because we only used initial CT scans taken for each patient in the slice model.

|  | **Training dataset** | | | **Validation dataset** | | |
| --- | --- | --- | --- | --- | --- | --- |
|  | **Patients** | **Series** | **Slices** | **Patients** | **Series** | **Slices** |
| **Osaka General Medical Center** | | | | | | |
| COVID-19 | 94 | 94 | 3398 | 23 | 23 | 912 |
| OLD | 99 | 99 | 4398 | 23 | 23 | 1035 |
| Normal | 79 | 79 | 3749 | 21 | 21 | 982 |
| **Teikyo University Hospital** | | | | | | |
| COVID-19 | 67 | 67 | 1480 | 15 | 15 | 287 |
| OLD | 838 | 838 | 42130 | 200 | 200 | 10030 |
| **Shonan Kamakura General Hospital** | | | | | | |
| COVID-19 | 280 | 280 | 7232 | 74 | 74 | 1825 |
| **Juntendo University Urayasu Hospital** | | | | | | |
| COVID-19 | 26 | 26 | 790 | 4 | 4 | 71 |
| **IUHW Narita Hospital** | | | | | | |
| COVID-19 | 59 | 59 | 1615 | 18 | 18 | 586 |
| **Tokyo Women's Medical University Hospital** | | | | | | |
| COVID-19 | 93 | 93 | 2314 | 22 | 22 | 589 |
| **Osaka City General Hospital** | | | | | | |
| COVID-19 | 87 | 87 | 4176 | 11 | 11 | 539 |
| **Nara Prefecture General Medical Center** | | | | | | |
| COVID-19 | 51 | 51 | 1935 | 10 | 10 | 362 |
| **Tsuyama Chuo Hospital** | | | | | | |
| COVID-19 | 10 | 10 | 262 | 4 | 4 | 116 |
| OLD | 69 | 69 | 3110 | 15 | 15 | 657 |
| Normal | 64 | 64 | 2841 | 19 | 19 | 871 |
| **Showa University Hospital** | | | | | | |
| COVID-19 | 31 | 31 | 671 | 6 | 6 | 185 |
| OLD | 114 | 114 | 5249 | 24 | 24 | 1111 |
| **Shizuoka Saiseikai General Hospital** | | | | | | |
| OLD | 304 | 304 | 29215 | 90 | 90 | 8569 |
| Normal | 367 | 367 | 38444 | 103 | 103 | 10567 |

**Table 8.3 Training settings of the slice model**

| **Training settings (slice model)** | |
| --- | --- |
| Loss function | Cross entropy loss |
| Optimizer | Momentum SGD (momentum = 0.9, weight_decay = 0.0001) |
| Mini-batch size | 48 |
| Max epoch | 25 |
| Learning rate | Starts at 0.01, decays to 1/10 at 10 and 15 epochs (step decay method) |

###

Each transformation was executed with a 50% probability for each iteration. The angle for random rotation was an integer in degrees, which was determined by rounding a random value based on a normal distribution with a mean of 0 and a standard deviation of 10. The size of the noisy image added by random erasing was determined based on random numbers generated using a uniform distribution of 0.02–0.4 times the original image width and an aspect ratio of 0.3–3.3. The noisy image replaces pixel values with random numbers following a uniform distribution in the range [0, 1]. Figure 8.1 shows examples of the transformations described above. The images shown in the figure represent the input data for training the slice model.


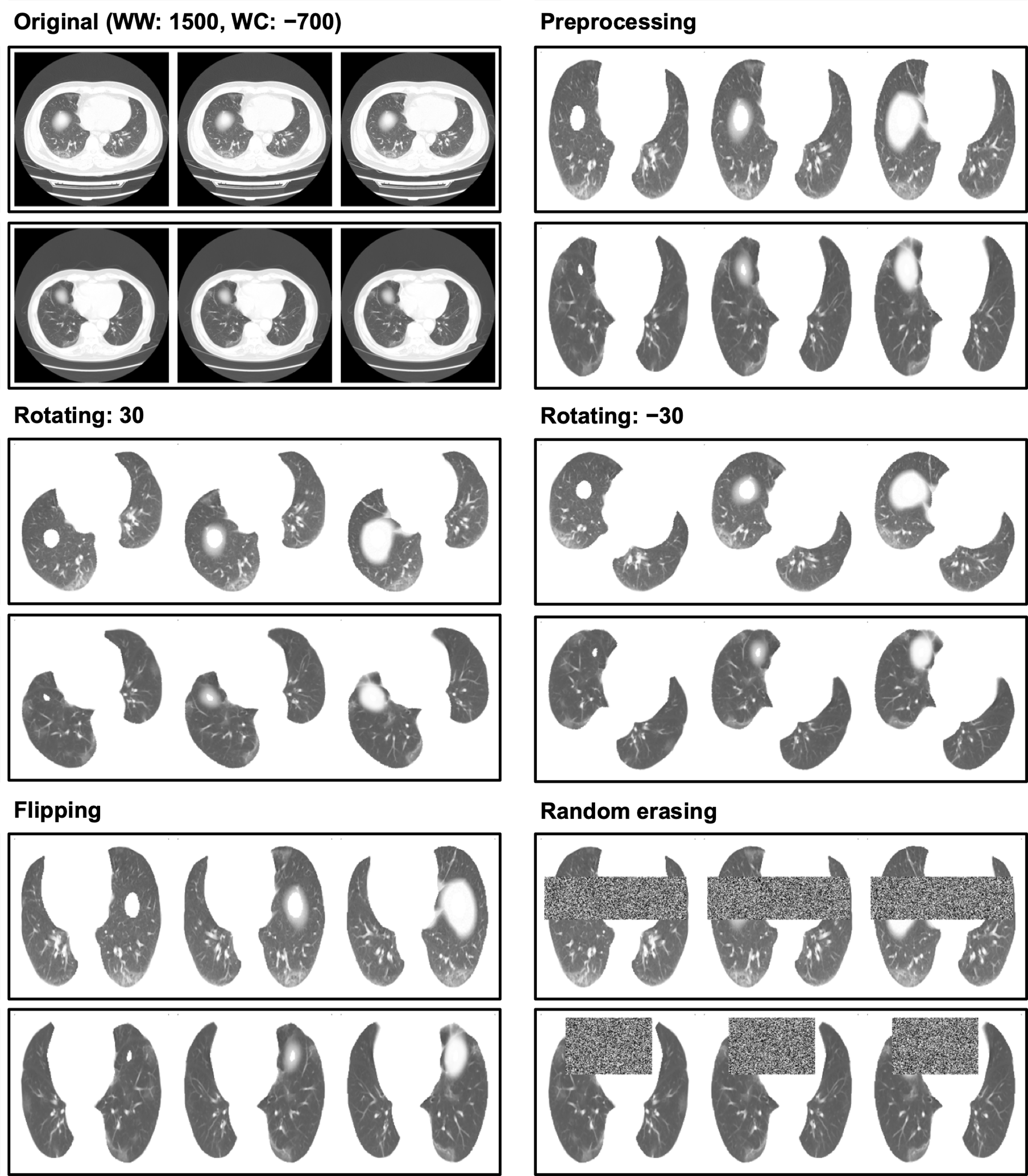


**Figure 8.1 Examples of the transformations for the slice model:** “Original image,” “Pre-processed,” and “Rotating,” “Flipping,” and “Random erasing” represent original CT slices after the windowing operation, images after the pre-processing described in Supplementary Section 7, and the images after the corresponding transformations, respectively.

The learning curve resulting from these settings is shown in Figure S8.2. The left and right vertical axes represent the log scale loss and accuracy, respectively. The training loss and accuracy for each iteration and the validation loss and accuracy for each epoch are shown in Figure 8.2. The model with the lowest validation loss was selected as the final model. The validation loss was the minimum at the 11th epoch; after that, the training loss decreased, whereas the validation loss increased slowly. Thus, the checkpoint stored at the 11th epoch is adopted as the final model.


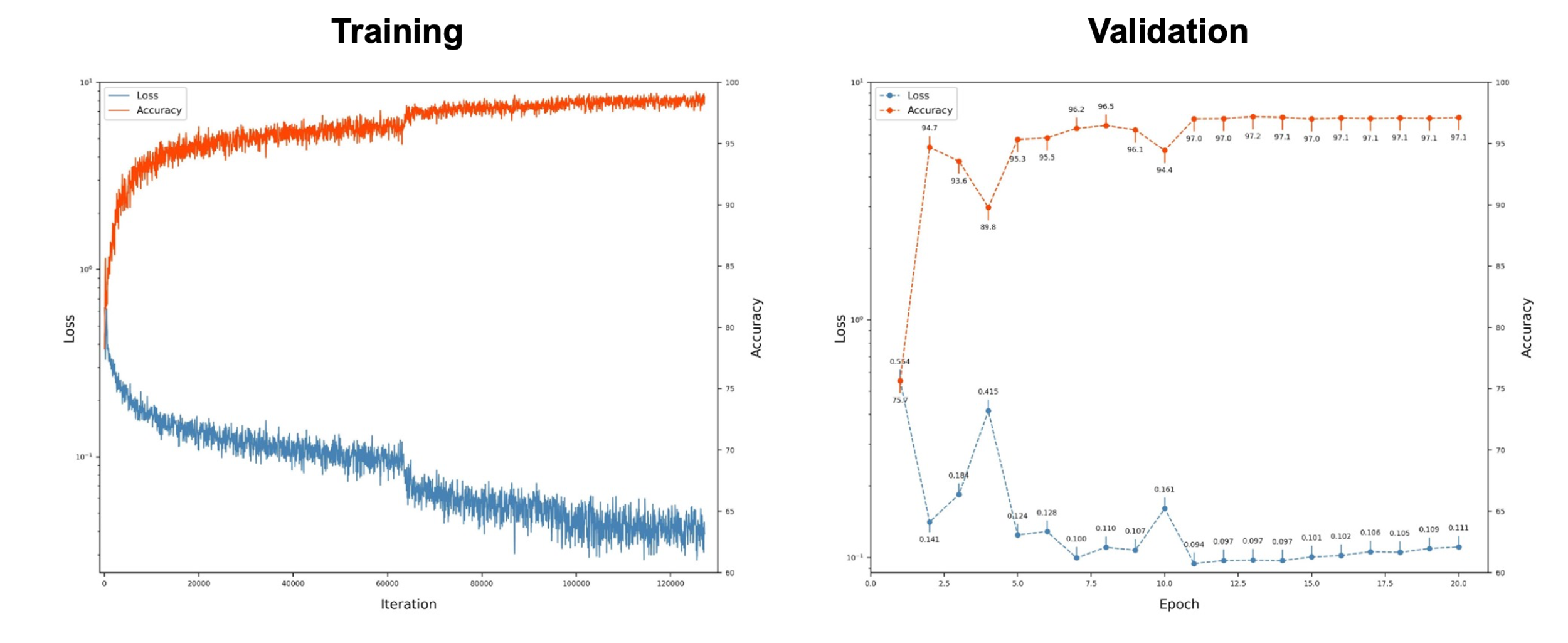


**Figure 8.2 The learning curve of the slice model:** “Accuracy” indicates the accuracy while training and validation, respectively; “Loss” indicates the training and validation loss, respectively; and the left vertical axis represents the log scale loss, and the right vertical axis represents the accuracy.

### Training the series model

The training data for the series model consisted of 1,400 COVID-19, 1,498 OLD, and 528 normal series. The validation data consisted of 347 COVID-19, 384 OLD, and 129 normal series. The breakdown of data by the institution is presented in Table 8.4. For the series model, the CT series taken at the initial and follow-up periods increase the data. For each series that was not excluded in Supplementary Section 5, 27 images were extracted and used for training as described in Methods section. COVID-19 cases were treated as positive, whereas OLD and normal cases were treated as negative. Therefore, there were 1,400 positive and 2,026 negative series in the training data and 347 positive and 513 negative series in the validation data.

We initialized the parameters with those of the slice model pre-trained using the training data and performed fine-tuning in all layers. Although the input image size is different, their structures are identical; thus, already trained parameters can be used in all layers. This fine-tuning was expected to make it easier for the series model to acquire disease features, though it had less training data than the slice model. Training hyperparameters were determined as follows;

- Optimizer selected from Momentum SGD^13^, Adam^14^, and AdamW^15^.
- Mini-batch size selected from [1, 4, 8, 10].
- Weight decay selected from [0.00001, 0.00005, 0.0001, 0.0005, 0.001, 0.005, 0.001].
- Initial learning rate selected from [0.1, 0.05, 0.01, 0.005, 0.001].

The best result was achieved using the settings listed in Table 8.5.

**Table 8.4 Training and validation data for the series model:** “Patients” and “Series” represent the number of patients included and the number of series used, respectively.

|  | **Training dataset** | | **Validation dataset** | |
| --- | --- | --- | --- | --- |
|  | **Patients** | **Series** | **Patients** | **Series** |
| **Osaka General Medical Center** | | | | |
| COVID-19 | 96 | 180 | 21 | 34 |
| OLD | 104 | 104 | 18 | 18 |
| Normal | 77 | 77 | 23 | 23 |
| **Teikyo University Hospital** | | | | |
| COVID-19 | 65 | 66 | 17 | 18 |
| OLD | 840 | 881 | 198 | 219 |
| **Shonan Kamakura General Hospital** | | | | |
| COVID-19 | 277 | 558 | 77 | 159 |
| **Juntendo University Urayasu Hospital** | | | | |
| COVID-19 | 26 | 43 | 4 | 5 |
| **IUHW Narita Hospital** | | | | |
| COVID-19 | 62 | 188 | 15 | 50 |
| **Tokyo Women's Medical University Hospital** | | | | |
| COVID-19 | 94 | 104 | 21 | 24 |
| **Osaka City General Hospital** | | | | |
| COVID-19 | 81 | 142 | 17 | 24 |
| **Nara Prefecture General Medical Center** | | | | |
| COVID-19 | 47 | 63 | 14 | 20 |
| **Tsuyama Chuo Hospital** | | | | |
| COVID-19 | 12 | 23 | 2 | 2 |
| OLD | 59 | 78 | 25 | 30 |
| Normal | 72 | 76 | 11 | 11 |
| **Showa University Hospital** | | | | |
| COVID-19 | 27 | 33 | 10 | 11 |
| OLD | 109 | 125 | 29 | 33 |
| **Shizuoka Saiseikai General Hospital** | | | | |
| OLD | 310 | 310 | 84 | 84 |
| Normal | 375 | 375 | 95 | 95 |

**Table 8.5 Training settings of the series model**

| **Training settings (series model)** | |
| --- | --- |
| Loss function | Cross entropy loss |
| Optimizer | Momentum SGD (momentum = 0.9, weight_decay = 0.0001) |
| Mini-batch size | 10 |
| Max epoch | 50 |
| Learning rate | Starts at 0.01, decays to 1/10 at 10 and 15 epochs (step decay method) |

Like the slice model, we performed random rotation, random flip, and random erasing during data augmentation. Each transformation was executed with a 50% probability for each iteration. The angle for random rotation was determined similarly to the slice model, and if performed, all 27 slice images were rotated by the same angle. A random flip was applied to all 27 slice images when it was performed. The parameters for random erasing were the same as those for the slice model, and noisy images were added to the tile images and not to the individual slice images. Figure 8.3 shows examples of the transformations described above. The images shown in the figure represent input data for training the series model.

The learning curve resulting from these settings is shown in Figure 8.4. Like the slice model, the left vertical axis is the log scale loss, and the right vertical axis shows accuracy. The validation loss was minimal at the 37th epoch, and the checkpoint at that epoch was adopted as the final model.


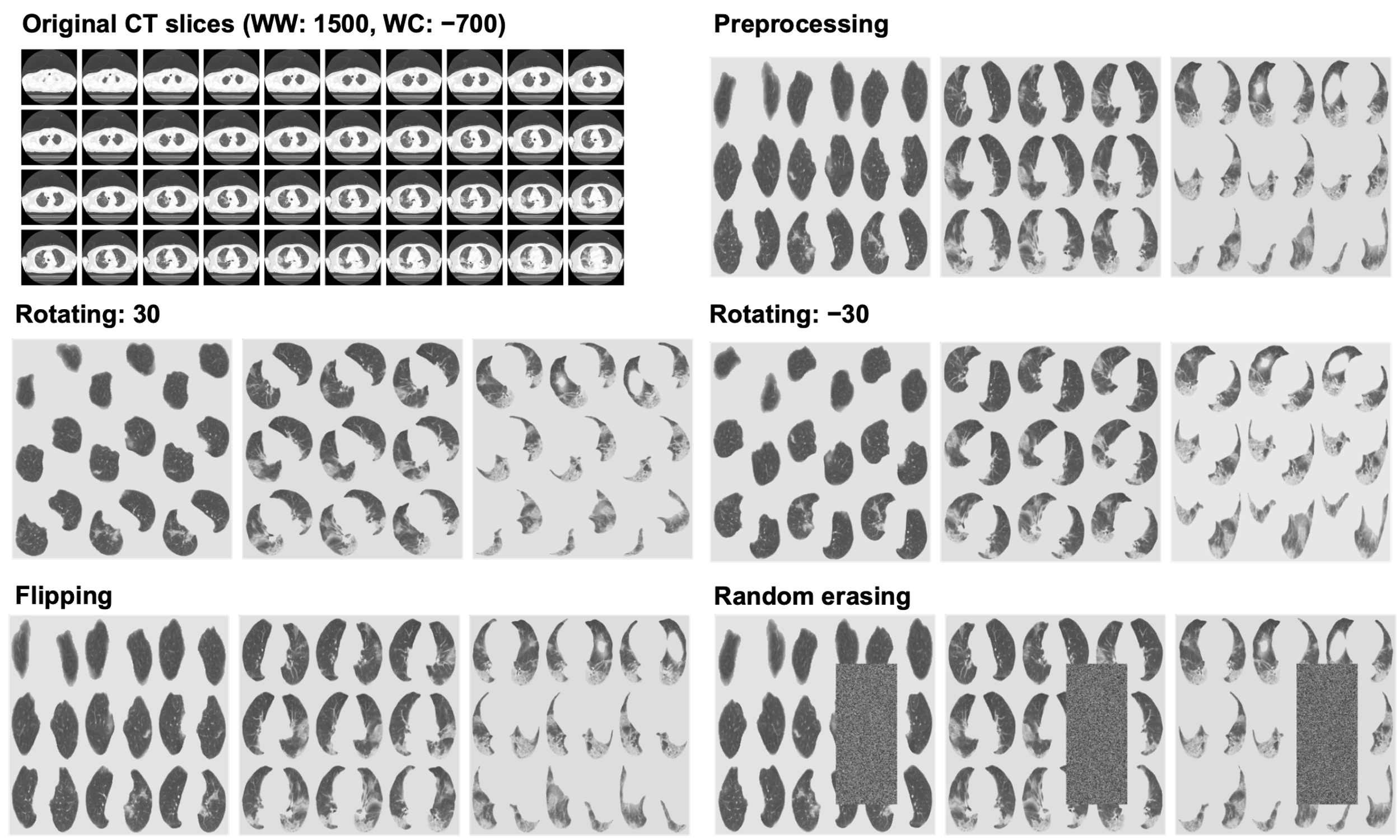


**Figure 8.3 Examples of the transformations for the series model:** “Original CT slices,” “Pre-processed,” and “Rotating,” “Flipping,” and “Random erasing” represent the original CT slices after the windowing operation, tiled images generated in the pre-processing described in Supplementary Section 7, and images after the corresponding transformations, respectively.


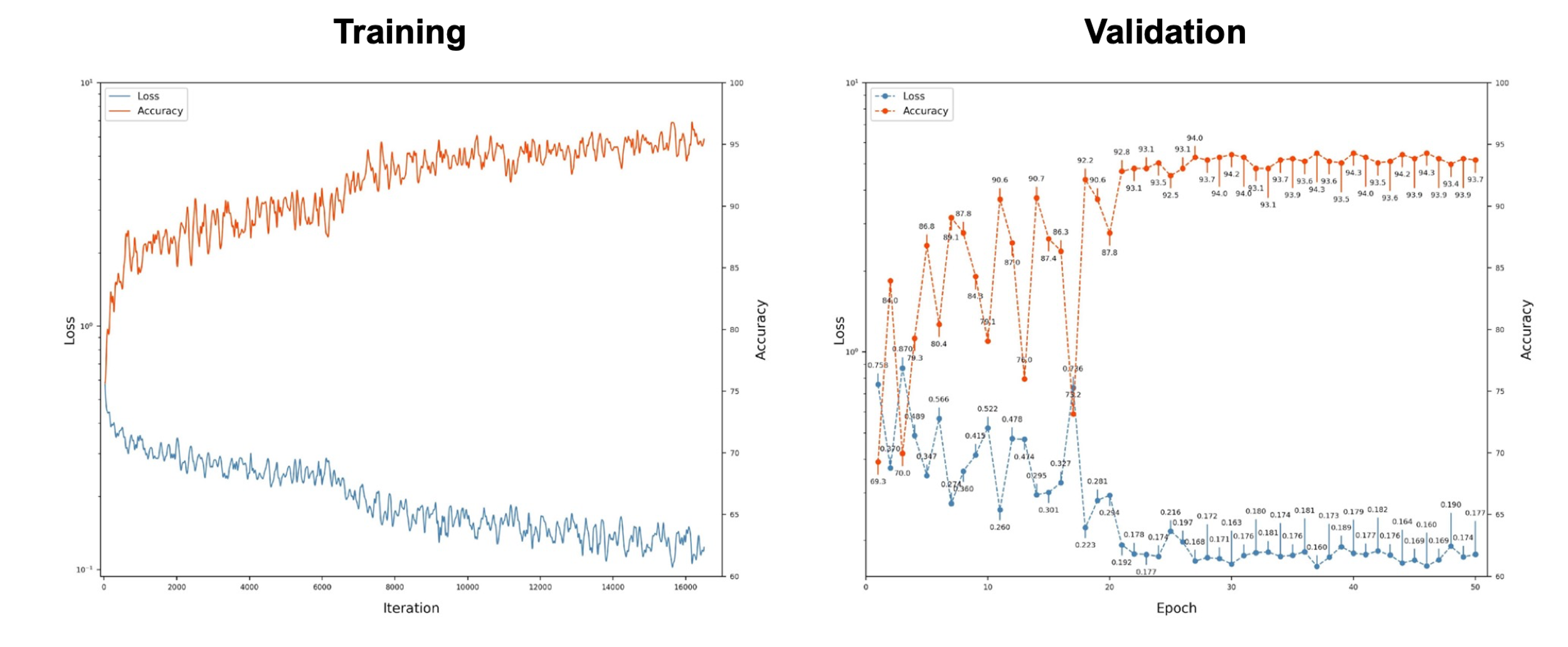


**Figure 8.4 The learning curve of the series model:** “Accuracy” indicates the accuracy while training and validation, respectively; “Loss” indicates the training and validation loss, respectively; and the left vertical axis represents the log scale loss, and the right vertical axis represents the accuracy.

## Supplementary Section 9: Saliency maps

We generated saliency maps in the following steps using the validation data under the same environment as during the training described in Supplementary Section 8.

1. We executed the forward pass of the model.
2. We performed backpropagation, treating the class with the highest confidence score as the true label.
3. The loss function gradient for each input image pixel was computed, and the absolute values were visualized.

The saliency maps obtained in these steps can be interpreted as heat maps showing how well the model responds to each pixel in the input image.

Figure 9.1 (a)–(e) show the saliency maps of the slice model. Figure 9.1 (a) and (b) show the saliency maps for COVID-19. The slice model responded to ground-glass opacities and nodules in image (a). The slice model did not respond to dorsal consolidation or pleural effusion but to ground-glass opacities and nodules in image (b). Figure 9.1 (c) and (d) show saliency maps for cases of pneumonia other than COVID-19. Similarly, the slice model responded to ground-glass opacities and nodules in these cases. Figure 9.1 (e) shows the saliency maps for the normal case. In this case, the slice model responds to linear opacities.

Figure 9.2 (a)–(e) show saliency maps of the series model. Figure 9.2 (a) and show saliency maps for COVID-19. The series model did not respond to dorsal consolidation or pleural effusion but responded to ground-glass opacities and nodules. Figure 9.2 (c) and (d) show saliency maps for cases of pneumonia other than COVID-19. Similarly, the series model responded to ground-glass opacities and nodules in these cases. Figure 9.2 (e) shows the saliency maps for the normal case.


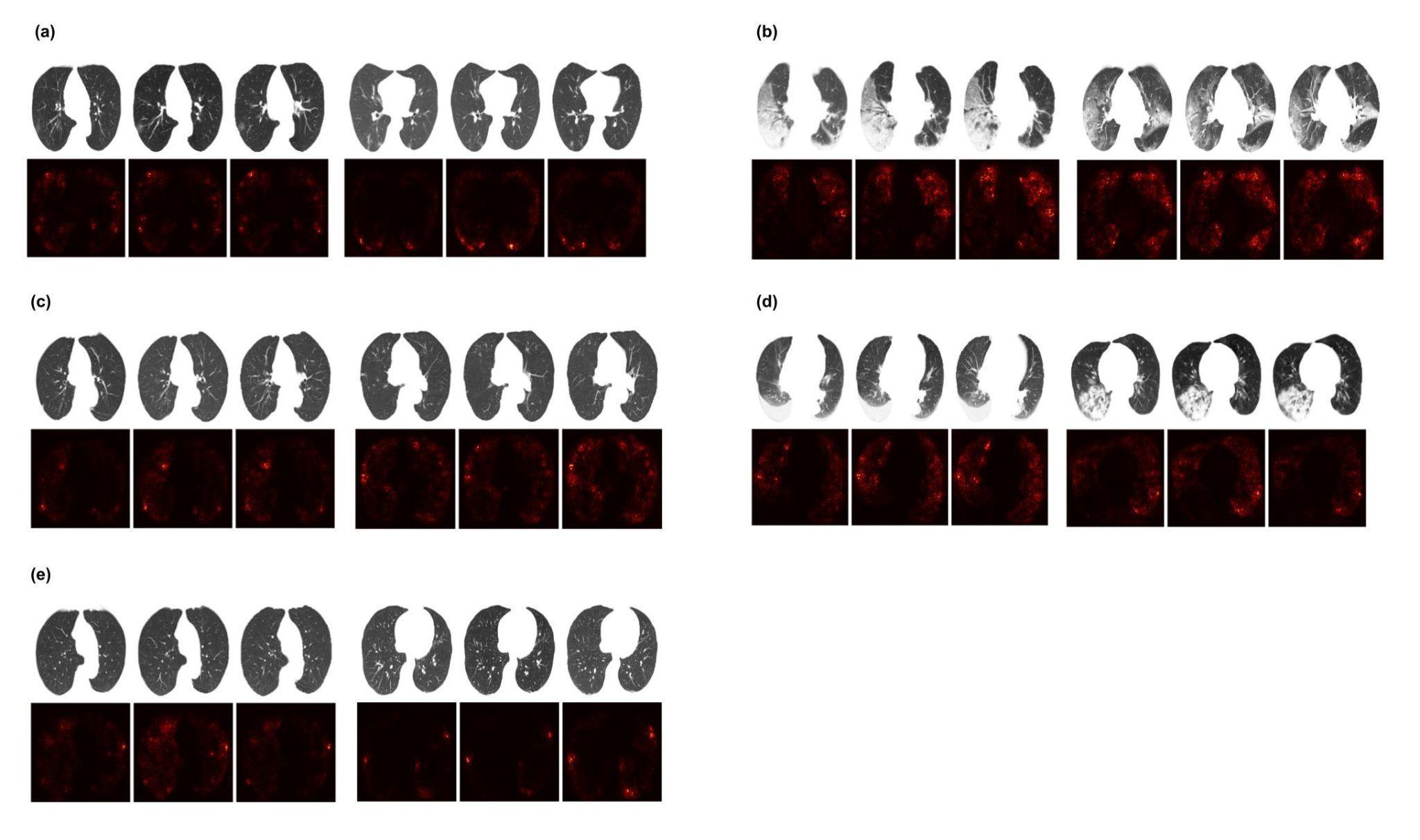


**Figure 9.1: Saliency maps of the slice model:** The input images for the slice model are shown in the top rows, and their corresponding saliency maps are shown in the bottom rows. The pixels with high values in the saliency maps are shown in red. (a): Saliency maps of the slice model for a case of mild COVID-19. (b): Saliency maps of the slice model for a case of severe COVID-19. (c): Saliency maps of the slice model for a case of mild pneumonia other than COVID-19. (d): Saliency maps of the slice model for a case of severe pneumonia other than COVID-19. (e): Saliency maps of the slice model for a normal case.


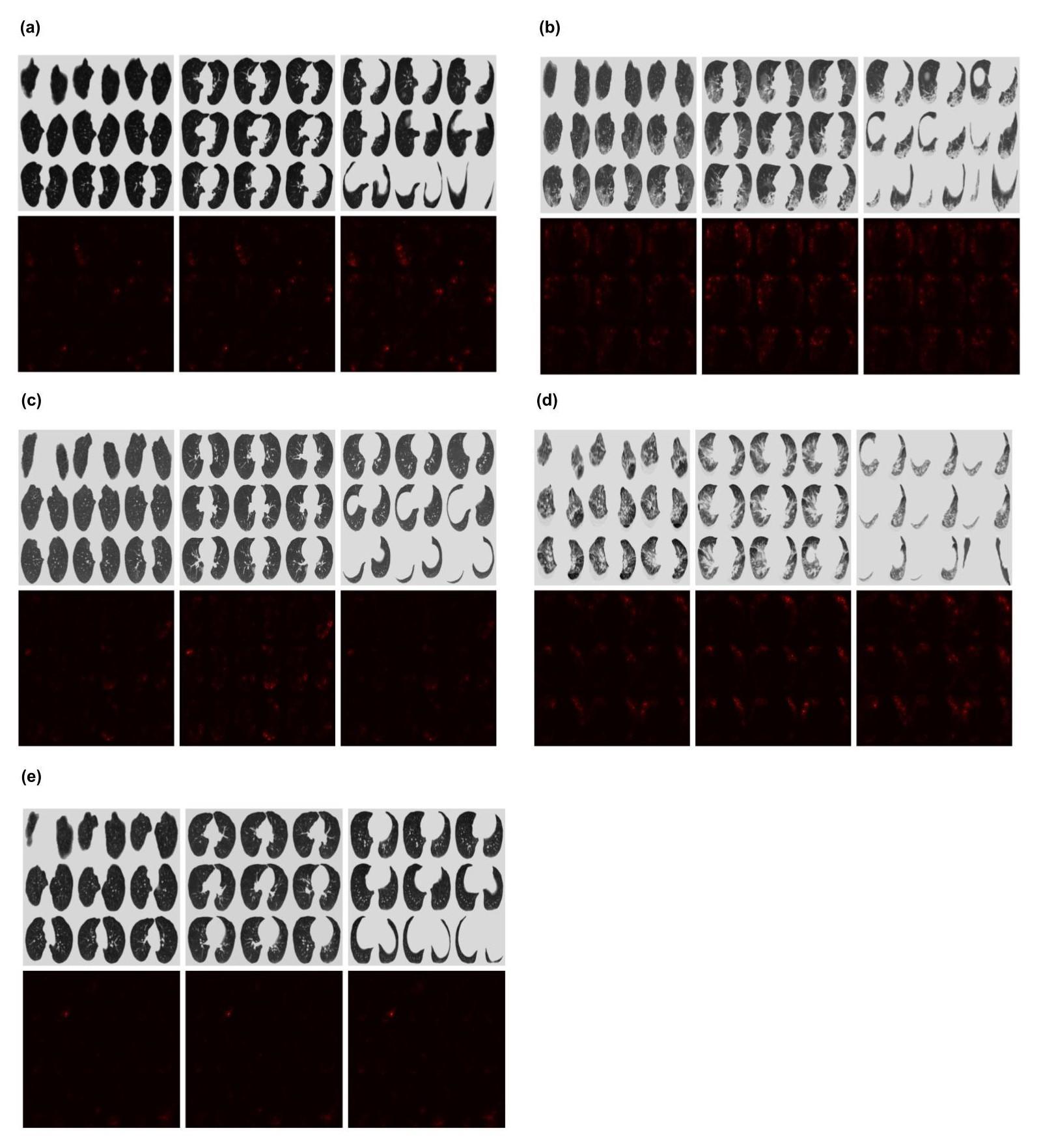


**Figure 9.2: Saliency maps of the series model:** The input images for the series model are shown in the top rows, and their corresponding saliency maps are shown in the bottom rows. The pixels with high values in the saliency maps are shown in red. (a): Saliency maps of the series model for a case of mild COVID-19. (b): Saliency maps of the series model for a case of severe COVID-19. (c): Saliency maps of the series model for a case of mild pneumonia other than COVID-19. (d): Saliency maps of the series model for a case of severe pneumonia other than COVID-19. (e): Saliency maps of the series model for a normal case.

## Supplementary Section 10: Inference environment and processing

### Inference environment

We performed experiments on the inference process using two trained models with Razer Blade 15 advanced 2020, a commercially available GPU-equipped laptop PC (model number: RZ09-03305J43-R3J1, GPU: NVIDIA GeForce RTX 2080 Super Max-Q 8G, CPU: Intel i7-10875H 8-core, memory: 16 GB RAM).

Since PCs are used in all aspects of daily clinical practice, we selected a laptop-type PC with fewer placement restrictions than desktop PCs. Our inference process loads data from the local storage, and all processes are performed on hardware without communication with an external network. Thus, our process has no risk of data leakage and no delay in data transmission.

The operating environment for the inference process was created on the hardware using the following steps:

1. Changed the OS to Ubuntu 20.04.1 LTS
2. Installed the NVIDIA-driver-470
3. Installed docker (version 20.10.12) and NVIDIA Container Toolkit (version 1.7.0)
4. Built a docker image to run the inference process

The base image of the docker image built in step (4) was nvidia/cudagl:10.1-runtime-ubuntu18.04 (<https://hub.docker.com/layers/nvidia/cudagl/10.1-runtime-ubuntu18.04/images/sha256-84e222ce42d473348b2250c9f1095f7cc5e596962e0af7b8c8a7cf586a3a747e?context=explore>) on the docker hub. In this step, we installed Python 3.7.4, installed the Python packages described in Table 10.1, cloned from GitHub a version of lungmask (<https://github.com/fcuro-inc/lungmask>) that we had forked and added changes, copied the source code of our inference process into the Docker image file system, and modified PYTHONPATH and CUDA paths.

Further, we published the environment setup instructions described above for Zenodo (<https://doi.org/10.5281/zenodo.5835313>). The public documents include instructions for setting up the environment, automation scripts, an accessible playbook to run with the scripts, and the Dockerfile.

**Table 10.1 Python packages for inference**

| **Package name** | **Version** |
| --- | --- |
| addict | 2.4.0 |
| cython | 0.29.24 |
| fill_voids | 2.0.1 |
| gdcm | 1.1 |
| h5py | 2.10.0 |
| imagecodecs | 2021.8.26 |
| numpy | 1.20.0 |
| opencv-contrib-python | 4.0.0.21 |
| Pillow | 7.1.0 |
| pycryptodome | 3.11.0 |
| pydicom | 2.1.2 |
| pylibjpeg | 1.1.1 |
| pylibjpeg-libjpeg | 1.1.0 |
| PyYAML | 4.2b1 |
| pyzmq | 22.3.0 |
| scikit-image | 0.15.0 |
| scipy | 1.4.1 |
| SimpleITK | 2.0.2 |
| six | 1.12.0 |
| torch | 1.6.0 |
| tqdm | 4.48.2 |

### Inference process

Figure 10.1 (a) shows a flowchart of the inference process.

The two models share the same input data loading and pre-processing workflow by using context that stores the necessary state of each processing step as it is executed. The input data are loaded in the DICOM format. The input data’s 3–7 mm thick axial slices are loaded, and others are filtered out.

Pre-processing described in Supplementary Section 7 is applied to the loaded data. The pre-processed data are stored in a context to allow both models to handle them. Subsequently, the inference is performed using the series and slice models in sequence.

First, the series model creates tile images as its input, the format described in Methods section, and the model’s inference is executed. The inference results are stored in the context to merge with the results of the slice model. The slice model creates mini-batches of input slice images, and the model’s inference is executed. The selection of the optimal batch size for our inference process is described in Supplementary Section 15. Finally, the results of the slice model are merged with those of the series model stored in the context. This inference process is designed to speed up processing compared to the case where the slice and series models are run independently. For comparison, Figure 10.1 (b) shows the processing flow in that pattern.

The slice model takes the target slice and the slices before and after it as inputs to run the model and obtain the output. We repeat the process from data loading to model execution for each slice to apply it to an entire series. In contrast, the series model considers all slices as inputs for a single run. Although we can naively implement the inference processes independently by reusing the training source code with a few modifications, optimal execution speed still needs to be attained.

In contrast, we add three improvements to our inference process to obtain outputs for each case in a shorter time:

1. Commonization of data loading and pre-processing: The input data-loading process could be customized because the input for the slice and series models is the same DICOM file. In addition, the pre-processing of the input data, i.e., windowing, resizing, image smoothing, lung field detection, and filtering after lung field detection, are commonized. This standardization allows data loading and pre-processing to be performed only once for each case slice.
2. Part of the processing is omitted through sequence optimization of the pre-processing and execution of the slice model: We sorted all slices along the z-axis, applied processing from the top of the body, and omitted the pre-processing and execution of the slice model after the position where the lung fields are no longer detected. The omission is triggered when (i) at least 27 slices of the series contain the lung fields necessary for the series model and (ii) when the lung fields are not detected for three consecutive slices.
3. Adjustment of batch size for pre-processing and execution of the slice model: Our inference process is designed to load data on a series basis. The pre-processing and slice model can take a mini-batch, including multiple slices as input. We explore the optimal batch size for our inference environment.

The details of the inference process's execution performance compared with the case in which the slice and series models were run independently are described in Supplementary Section 15.

We published the source code for the inference process described above in Zenodo (<https://doi.org/10.5281/zenodo.5835313>).


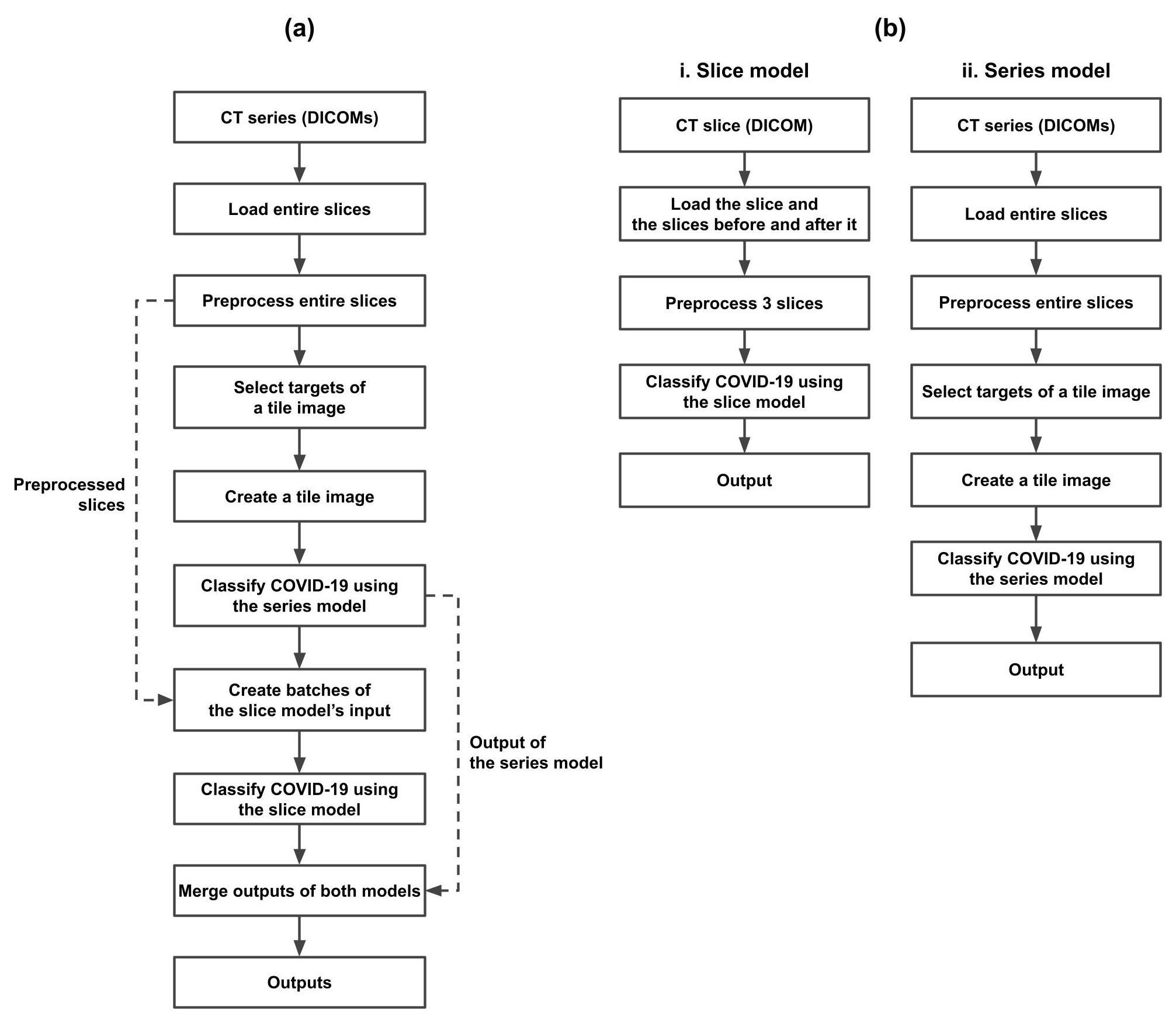


**Figure 10.1 Comparison of inference processes:** (a) Flowchart of our inference process. Dashed arrows indicate the use of outputs in the past steps. (b) Flowcharts of the processes when both models are run independently.

## Supplementary Section 11: Statistics method

### Agreement rate of labeling

Agreement rates for scores labeled by radiologists are calculated on a subgroup basis using Fleiss' kappa statistics^17^, frequently used for three or more raters^18^. The interpretations of the acquired agreement rates are provided with six-level labels based on the study of Landis et al.^19^: “Poor,” “Slight,” “Fair,” “Moderate,” “Substantial,” and “Almost Perfect,” for values under 0, from 0.00 to 0.20, from 0.21 to 0.40, from 0.41 to 0.60, from 0.61 to 0.80, and from 0.81 to 1.00, respectively. The mean values of the agreement rates and 95% confidence intervals are obtained for each subgroup (Table 6.1). The analysis results are acquired using NLTK (version 3.4.4) in Python 3.7.4.

### Model evaluation

For both models, the 95% confidence intervals for AUC, sensitivity, specificity, and accuracy are calculated using the bootstrap method^20^ during the evaluation with validation data. The specific methods used are described below:

- For each COVID-19 positive and negative value, sampling is performed with a replacement whose sampling frequency is identical to that of the population.
- The accuracy of the model is validated using the generated sample data.
- Based on the results obtained by repeating the above process 2000 times, we derived the mean value of the accuracy and 95% confidence interval.

Accuracy evaluation is performed in the training environment described in Supplementary Section 8. Sampling with replacement is performed using the random module in Python 3.7.4. The 95% confidence intervals are calculated using NumPy (version 1.20.0), the ROC curves are visualized using matplotlib (version 3.5.0), and the AUC calculations are performed using scikit-learn (version 1.0.1).

### Processing times

The 95% confidence intervals for the test dataset are derived for inference processing time using the bootstrap method^20^. The specific methods are described below:

- Sampling is performed with replacement, whose sampling frequency is identical to the total number of test data.
- The processing time is validated using the generated sample data.
- Based on the results obtained by repeating the above process 2000 times, we calculated the mean value of the processing time and the 95% confidence interval.

Sampling with the replacement and calculation of the 95% confidence interval is performed using NumPy (version 1.20.0).

## Supplementary Section 12: Detailed demographics of the dataset

### Data demographics per institution

Tables 12.1 and 12.2 present the breakdown of the patients in this study. Table 12.1 summarizes the patients for training and validation of the model, and Table 12.2 lists the patients for external testing of the model. Each table shows the distribution of sex and age of patients with COVID-19, OLD, and the normal group by the institution.

**Table 12.1 Patient demographics in training and validation.**

|  | **Sex (male rate)** | **Age** |
| --- | --- | --- |
| **Osaka General Medical Center** | | |
| COVID-19 | 84 males, 33 females (71.8%) | 28–94 (Ave. 62.4) |
| OLD | 77 males, 45 females (63.1%) | 18–95 (Ave. 58.1) |
| Normal | 77 males, 23 females (77.0%) | 18–88 (Ave. 45.7) |
| **Teikyo University Hospital** | | |
| COVID-19 | 49 males, 33 females (59.8%) | 20–87 (Ave. 53.3) |
| OLD | 620 males, 418 females (59.7%) | 18–97 (Ave. 61.4) |
| **Shonan Kamakura General Hospital** | | |
| COVID-19 | 207 males, 147 females (58.5%) | 20–94 (Ave. 65.0) |
| **Juntendo University Urayasu Hospital** | | |
| COVID-19 | 21 males, 9 females (70.0%) | 23–72 (Ave. 50.1) |
| **IUHW Narita Hospital** | | |
| COVID-19 | 43 males, 34 females (55.8%) | 19–88 (Ave. 54.5) |
| **Tokyo Women's Medical University Hospital** | | |
| COVID-19 | 78 males, 37 females (67.8%) | 21–95 (Ave. 51.3) |
| **Osaka City General Hospital** | | |
| COVID-19 | 79 males, 19 females (80.6%) | 29–89 (Ave. 67.5) |
| **Nara Prefecture General Medical Center** | | |
| COVID-19 | 45 males, 16 females (73.8%) | 23–90 (Ave. 62.4) |
| **Tsuyama Chuo Hospital** | | |
| COVID-19 | 7 males, 7 females (50.0%) | 23–87 (Ave. 67.8) |
| OLD | 61 males, 23 females (72.6%) | 22–95 (Ave. 73.7) |
| Normal | 44 males, 39 females (53.0%) | 26–97 (Ave. 71.7) |
| **Showa University Hospital** | | |
| COVID-19 | 23 males, 14 females (62.2%) | 20–87 (Ave. 64.3) |
| OLD | 87 males, 51 females (63.0%) | 28–101 (Ave. 77.5) |
| **Shizuoka Saiseikai General Hospital** | | |
| OLD | 241 males, 153 females (61.2%) | 23–101 (Ave. 74.1) |
| Normal | 212 males, 258 females (45.1%) | 18–95 (Ave. 61.9) |

**Table 12.2 Patient demographics in testing:** “Sex” indicates the number of people and the percentage of men for each group. “Age” indicates each group’s minimum, maximum, and average ages.

|  | **Sex (male rate)** | **Age** |
| --- | --- | --- |
| **Osaka General Medical Center** | | |
| COVID-19 | 73 males, 20 females (78.5%) | 34–94 (Ave. 71.2) |
| OLD | 16 males, 7 females (69.6%) | 24–95 (Ave. 69.6) |
| Normal | 4 males, 3 females (57.1%) | 56–102 (Ave. 76.4) |
| **National Hospital Organization Kyoto Medical Center** | | |
| COVID-19 | 12 males, 15 females (44.4%) | 42–93 (Ave. 67.0) |
| OLD | 82 males, 51 females (61.7%) | 24–98 (Ave. 73.1) |
| Normal | 66 males, 57 females (53.7%) | 18–99 (Ave. 58.5) |

### Patients’ disease demographics in the test dataset

A detailed breakdown of all comorbidities and 156 patients with OLD used for the external test data of the model is provided in Figures 12.1 (a), (b), and (c). Regarding the comorbidities, only thoracic diseases that could appear as visual findings in the lung fields were included.

Figure 12.1 (a) shows that the following diseases accompany the COVID-19 group: pulmonary emphysema in 24 patients, old inflammatory changes (fibrosis, calcification) in 22 patients, pulmonary edema and pleural effusion in 35 patients, bacterial pneumonia in 6 patients, pulmonary tumor in 2 patients, and others in 5 patients.

Figure 12.1 (b) shows the detailed breakdown of OLD: 81 bacterial pneumonia, 23 viral pneumonia, four atypical pneumonia, 27 interstitial lung disease (ILD), three cardiogenic pulmonary edema, two chronic obstructive pulmonary diseases (COPD), and 16 others (lung cancer, pneumothorax, hemothorax, lung contusion, pyothorax, pneumosilicosis). For the comorbidity findings, there were 24 patients with pulmonary emphysema, 12 with old inflammatory changes, 41 with pulmonary edema and pleural effusion, 10 with pulmonary tumors, and 15 with other diseases.

Figure 12.1 (c) shows the comorbidity findings in normal patients: emphysema in 18 patients, old inflammatory changes in 42 patients, small pleural effusion in 5 patients, and the other in one patient.


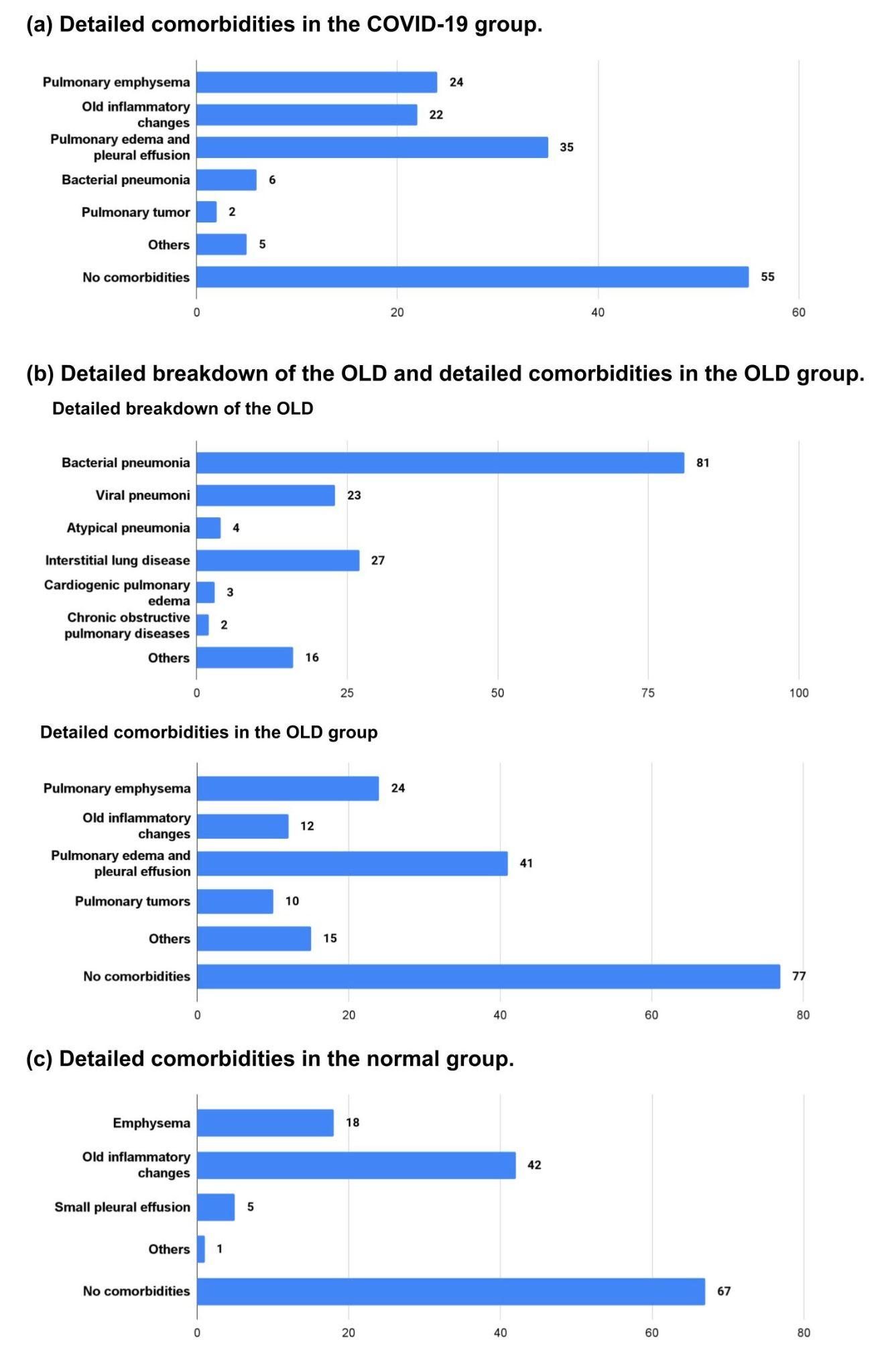


**Figure 12.1 Detailed comorbidities:** The number of patients with each comorbidity for each group is shown.

## Supplementary Section 13: Classification performance

The classification performances of the slice and series models on the validation data are illustrated in Figures 13.1 and Table 13.1. The ROC curves for both models are shown in Figure 13.1, and the accuracy for each threshold is summarized in Table 13.1.

In the validation dataset, the slice model distinguishes COVID-19 images from OLD and normal images with an AUC of 0.989 (95% CI: 0.986–0.991). With a threshold of 0.5, the sensitivity was 90.3% (95% CI: 89.5–91.1), the specificity was 98.1% (95% CI: 98.0–98.2), and the accuracy was 97.0% (95% CI: 96.9–97.2). With a threshold of 0.165, with the sensitivity exceeding 95%, the specificity was 96.0% (95% CI: 95.8–96.2), and the accuracy was 95.9% (95% CI: 95.7–96.0). With the threshold of 0.115, with the specificity exceeding 95%, the sensitivity was 95.7% (95% CI: 95.2–96.2), and the accuracy was 95.2% (95% CI: 95.0–95.4).

The series model classified COVID-19 patients with an AUC of 0.982 (95% CI: 0.966–0.993). With a threshold of 0.5, the sensitivity was 91.6% (95% CI: 88.5–94.5), the specificity was 95.7% (95% CI: 94.0–97.5), and the accuracy was 94.0% (95% CI: 92.4–95.7). With a threshold of 0.255, with the sensitivity exceeding 95%, the specificity was 91.6% (95% CI: 89.1–94.0), and the accuracy was 93.0% (95% CI: 91.3–94.7). At a threshold of 0.430, with the specificity exceeding 95%, the sensitivity and accuracy were 93.0 % (95% CI: 90.2–95.7) and 93.0% (95% CI: 90.2–95.7), and the accuracy was 94.3% (95% CI: 92.7–95.9).


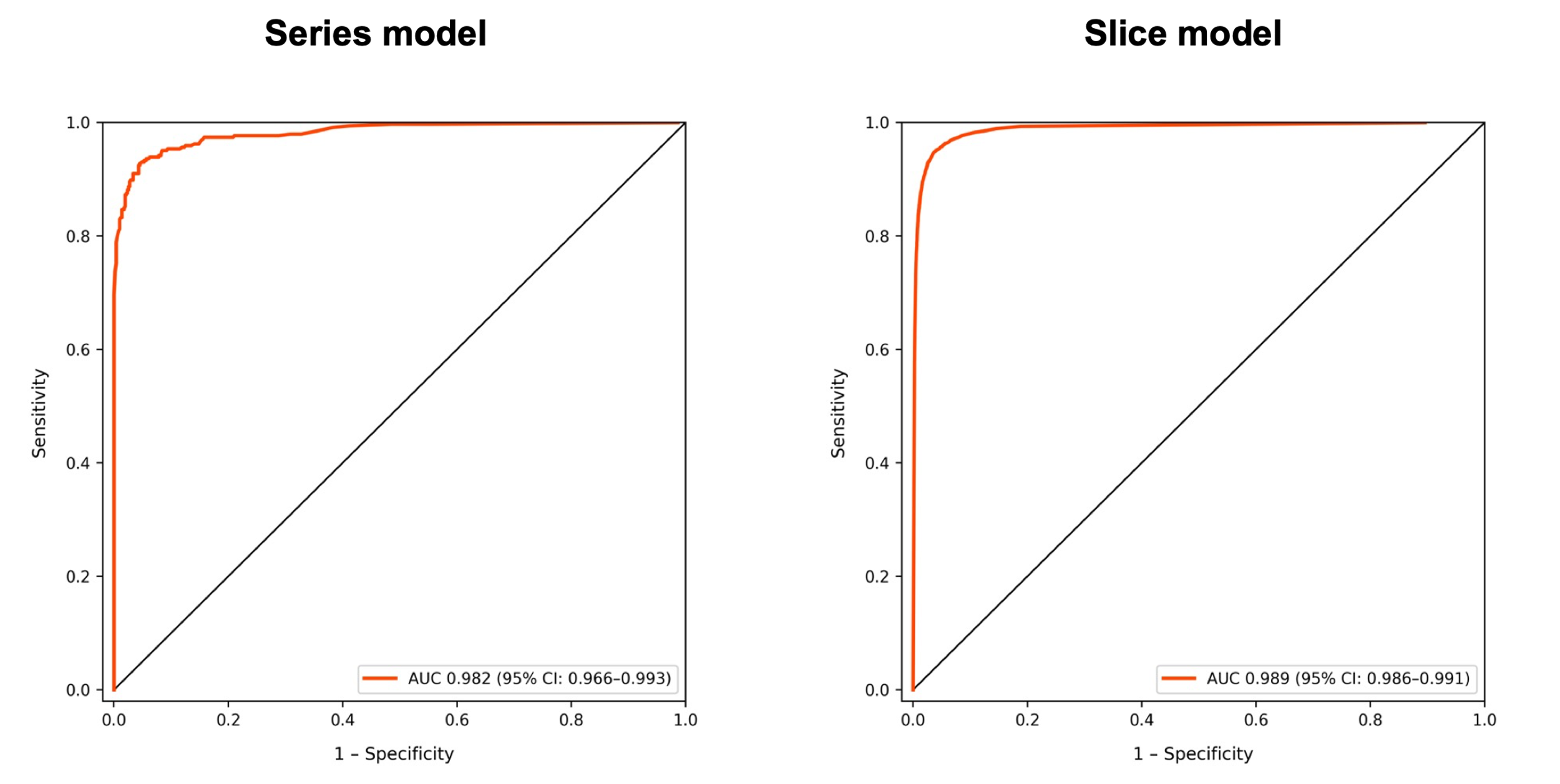


**Figure 13.1 ROC curves of our models at validation:** The ROC curves of the series and slice models for the validation data are shown in the figure. The AUC values and their 95% confidence intervals are presented.

**Table 13.1 Classification performance measures for the validation dataset:** “Threshold” represents the threshold used for separating positive and negative. For each threshold, the sensitivity, specificity, and accuracy of the series and slice models are shown in the table with their 95% confidence intervals.

| **Threshold** | **Accuracy [95% CI]** | **Sensitivity [95% CI]** | **Specificity [95% CI]** |
| --- | --- | --- | --- |
| **Slice model** | | | |
| 0.115 | 95.2 [95.0–95.4] | 95.7 [95.2–96.2] | 95.1 [94.8–95.3] |
| 0.165 | 95.9 [95.7–96.0] | 95.1 [94.5–95.6] | 96.0 [95.8–96.2] |
| 0.500 | 97.0 [96.9–97.2] | 90.3 [89.5–91.1] | 98.1 [98.0–98.2] |
| **Series model** | | | |
| 0.255 | 93.0 [91.3–94.7] | 95.1 [92.5–97.1] | 91.6 [89.1–94.0] |
| 0.430 | 94.3 [92.7–95.9] | 93.0 [90.2–95.7] | 95.1 [93.2–96.9] |
| 0.500 | 94.0 [92.4–95.7] | 91.6 [88.5–94.5] | 95.7 [94.0–97.5] |

Table 13.2 shows the validation dataset accuracy for each institution at a threshold of 0.5. The amount of data and correct answers are listed next to accuracy because the amount of data varies by institution. We can infer that our model can achieve sufficiently high accuracy, even under conditions in which the distribution of patients, imaging characteristics, or equipment differs from one institution to another.

**Table 13.2 Validation accuracy for each partner institution (threshold = 0.5):** The “series model” represents the accuracy of the series model for all series of validation datasets. The “slice model” represents the accuracy of the slice model for all slices of the validation dataset. A threshold of 0.5 was used for both models. The numbers in parentheses indicate the number of correct cases divided by the total number of cases.

|  | **Series model** | **Slice model** |
| --- | --- | --- |
| **Osaka General Medical Center** | | |
| COVID-19 | 88.2 (30/34) | 93.5 (853/912) |
| OLD | 94.4 (17/18) | 90.5 (937/1035) |
| Normal | 100.0 (23/23) | 99.7 (979/982) |
| **Teikyo University Hospital** | | |
| COVID-19 | 88.9 (16/18) | 83.6 (240/287) |
| OLD | 94.1 (206/219) | 96.2 (9654/10030) |
| **Shonan Kamakura General Hospital** | | |
| COVID-19 | 97.5 (155/159) | 92.0 (1679/1825) |
| **Juntendo University Urayasu Hospital** | | |
| COVID-19 | 100.0 (5/5) | 94.3 (67/71) |
| **IUHW Narita Hospital** | | |
| COVID-19 | 82.0 (41/50) | 97.7 (573/586) |
| **Tokyo Women's Medical University Hospital** | | |
| COVID-19 | 83.3 (20/24) | 97.2 (572/588) |
| **Osaka City General Hospital** | | |
| COVID-19 | 91.7 (22/24) | 75.7 (408/539) |
| **Nara Prefecture General Medical Center** | | |
| COVID-19 | 95.0 (19/20) | 82.6 (299/362) |
| **Tsuyama Chuo Hospital** | | |
| COVID-19 | 100.0 (2/2) | 90.5 (105/116) |
| OLD | 93.3 (28/30) | 90.1 (592/657) |
| Normal | 100.0 (11/11) | 98.0 (854/871) |
| **Showa University Hospital** | | |
| COVID-19 | 72.7 (8/11) | 78.4 (145/185) |
| OLD | 84.8 (28/33) | 96.5 (1072/1111) |
| **Shizuoka Saiseikai General Hospital** | | |
| OLD | 98.8 (83/84) | 99.5 (8530/8569) |
| Normal | 100.0 (95/95) | 99.9 (10565/10567) |

Figure 13.2 (a) and (b) and Tables 13.3 (a) and (b) show and list the classification performances of the slice and series models on external test data, respectively. The ROC curves for the slice and series models are shown in Figure 13.2 (a) and (b), and the accuracies for each threshold are shown in Tables 13.3 (a) and (b), respectively. In the external test dataset, the slice model detected COVID-19 with an AUC of 0.958 (95% CI: 0.937–0.974). With a threshold of 0.5, the sensitivity was 80.3% (95% CI: 75.7–84.2), the specificity was 95.0% (95% CI: 93.6–96.3), and the accuracy was 91.4% (95% CI: 90.0–92.7). With a threshold of 0.165, the sensitivity was 88.4% (95% CI: 85.0–91.3), the specificity was 90.6% (95% CI: 88.8–92.3), and the accuracy was 90.0% (95% CI: 88.5–91.6). With a threshold of 0.115, the sensitivity was 90.4% (95% CI: 87.3–93.1), the specificity was 89.2% (95% CI: 87.2–91.0), and the accuracy was 89.5% (95% CI: 87.9–91.0).

The series model detected COVID-19 with an AUC of 0.953 (95% CI: 0.907–0.986). With a threshold of 0.5, the sensitivity was 90.0% (95% CI: 84.2–95.0), the specificity was 94.1% (95% CI: 91.2–96.6), and the accuracy was 92.9% (95% CI: 90.3–95.2). With a threshold of 0.255, the sensitivity was 92.5% (95% CI: 87.6–96.7), the specificity was 90.9% (95% CI: 87.6–94.1), and the accuracy was 91.4% (95% CI: 88.8–94.1). With a threshold of 0.43, the sensitivity was 91.7% (95% CI: 86.3–96.4), the specificity was 93.4% (95% CI: 90.4–96.2), and the accuracy was 92.9% (95% CI: 90.3–95.3).

Further, we compared the accuracy of classifying the external test data for each patient's background and disease in the series model, with a threshold of 0.5. Table 13.4 (a) shows the accuracy according to patient sex. For males, the accuracy was 89.4% for COVID-19, 89.8% for OLD, and 97.1% for normal, while for females, the accuracy was 91.4% for COVID-19, 93.1% for OLD, and 100% for normal. As a result, the accuracy was nearly equal between males and females.


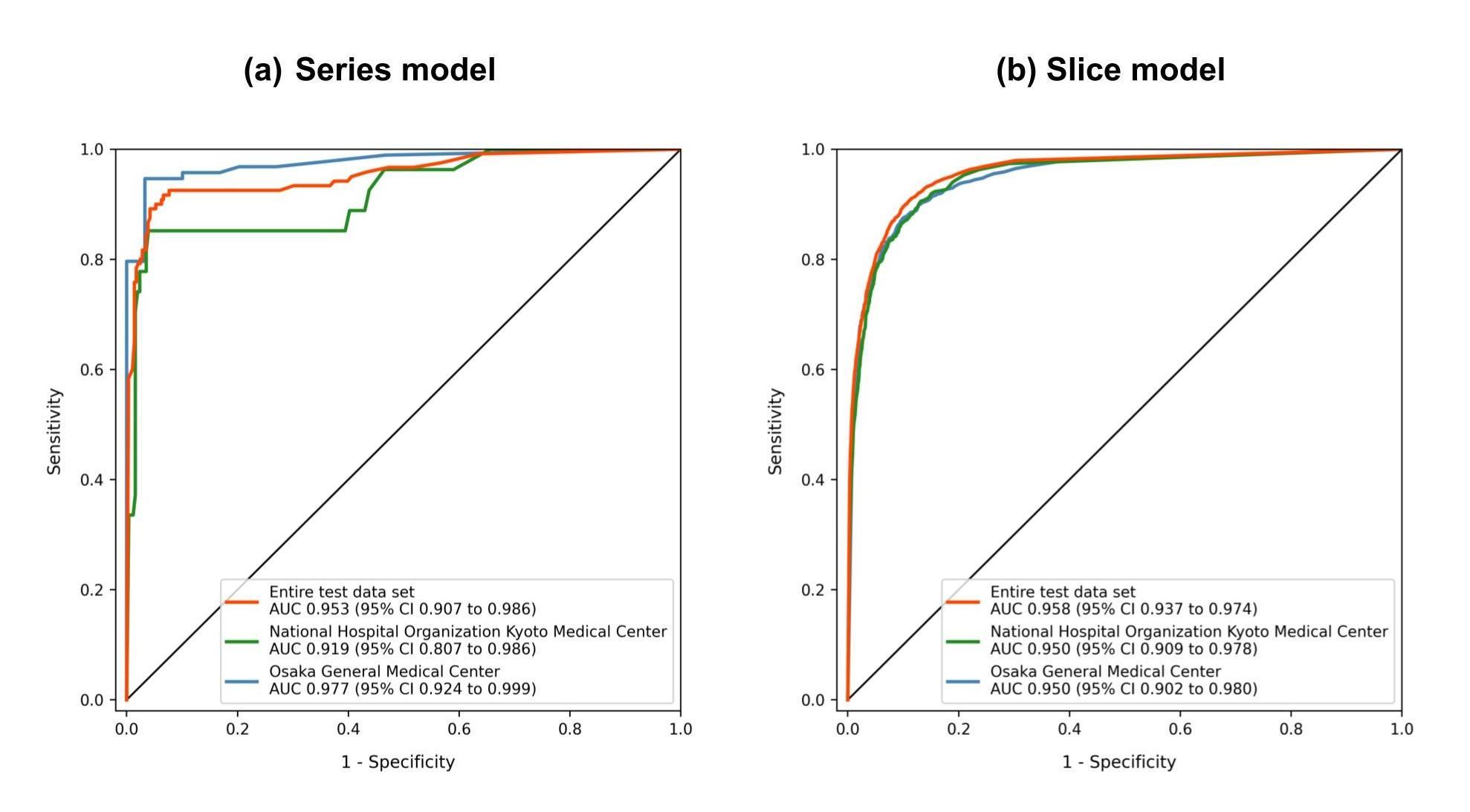


**Figure 13.2 ROC curves of our models at external testing:** The figures show the ROC curves for the entire test dataset and each institution (Osaka General Medical Center and National Hospital Organization Kyoto Medical Center) for each model. The AUC values and 95% confidence intervals are shown in the figures.

**Table 13.3: Classification performance measures for the test dataset.**

**(a): Classification performance measures of the series model for the test dataset.**

“Threshold” represents the threshold used for separating positive and negative. For each threshold, the sensitivity, specificity, and accuracy of the series model for the entire test dataset and each institution are shown in the table with 95% confidence intervals.

| **Threshold** | **Accuracy [95% CI]** | **Sensitivity [95% CI]** | **Specificity [95% CI]** |
| --- | --- | --- | --- |
| **Total** | | | |
| 0.255 | 91.4 [88.8–94.1] | 92.5 [87.6–96.7] | 90.9 [87.6–94.1] |
| 0.430 | 92.9 [90.3–95.3] | 91.7 [86.3–96.4] | 93.4 [90.4–96.2] |
| 0.500 | 92.9 [90.3–95.2] | 90.0 [84.2–95.0] | 94.1 [91.2–96.6] |
| **Osaka General Medical Center** | | | |
| 0.255 | 94.3 [90.0–97.7] | 94.7 [89.8–98.9] | 93.4 [83.3–100.0] |
| 0.430 | 94.3 [89.9–97.7] | 93.6 [87.9–97.9] | 96.7 [89.7–100.0] |
| 0.500 | 92.7 [87.9–96.8] | 91.4 [85.1–96.7] | 96.7 [89.7–100.0] |
| **National Hospital Organization Kyoto Medical Center** | | | |
| 0.255 | 90.1 [86.7–93.5] | 85.2 [69.6–96.6] | 90.6 [87.1–94.1] |
| 0.430 | 92.2 [89.1–95.3] | 85.2 [69.6–96.6] | 93.0 [89.7–94.1] |
| 0.500 | 92.9 [89.9–95.8] | 85.2 [69.6–96.6] | 93.8 [90.6–96.5] |

**(b): Classification performance measures of the slice model for the test dataset.**

“Threshold” represents the threshold used for separating positive and negative. For each threshold, the sensitivity, specificity, and accuracy of the slice model for the entire test dataset and each institution are shown in the table with 95% confidence intervals.

| **Threshold** | **Accuracy [95% CI]** | **Sensitivity [95% CI]** | **Specificity [95% CI]** |
| --- | --- | --- | --- |
| **Total** | | | |
| 0.115 | 89.5 [87.9–91.0] | 90.4 [87.3–93.1] | 89.2 [87.2–91.0] |
| 0.165 | 90.0 [88.5–91.6] | 88.4 [85.0–91.3] | 90.6 [88.8–92.3] |
| 0.500 | 91.4 [90.0–92.7] | 80.3 [75.7–84.2] | 95.0 [93.6–96.3] |
| **Osaka General Medical Center** | | | |
| 0.115 | 89.5 [86.4–92.2] | 91.1 [87.7–93.9] | 85.0 [77.4–91.0] |
| 0.165 | 88.7 [85.6–91.5] | 89.2 [85.6–92.2] | 87.5 [80.5–92.9] |
| 0.500 | 84.5 [80.6–87.7] | 80.9 [76.0–85.1] | 94.1 [88.6–97.9] |
| **National Hospital Organization Kyoto Medical Center** | | | |
| 0.115 | 89.5 [87.5–91.3] | 87.1 [78.8–93.4] | 89.6 [87.6–91.6] |
| 0.165 | 90.5 [88.6–92.3] | 84.9 [75.9–91.9] | 90.9 [89.1–92.7] |
| 0.500 | 94.0 [92.5–95.4] | 77.7 [66.7–86.8] | 95.1 [93.7–96.4] |

Table 13.4 (b) shows the accuracy for each patient age group. For patients aged 18–39 years, the accuracy was 100% for COVID-19, 90.0% for OLD, and 100% for normal. For patients aged 40–59 years, the rates were 87.5% for COVID-19, 93.3% for OLD, and 96.3% for normal. For patients aged 60–79 years, the rates were 94.0% for COVID-19, 88.6% for OLD, and 100% for normal. For patients aged 60–79 years, 94.0% had COVID-19, 88.6% had OLD, and 100% were normal. For patients 80 years and older, 81.5% had COVID-19, 93.4% had OLD, and 96.2% were normal. As a result, it indicated a trend toward lower accuracy in COVID-19 patients over 80 years of age. Table 13.4 (c) shows the percentage of correct answers for each comorbidity. When bacterial pneumonia coexisted, the accuracy was 100% for COVID-19 patients and 100% for OLD patients. OLD with comorbid ILD had a 100% accuracy. When pulmonary emphysema coexisted, the accuracy was 79.2% for COVID-19, 83.3% for OLD, and 100% for normal cases. When pleural effusion coexisted, the accuracy was 87.1% for COVID-19, 94.3% for OLD, and 100% for normal. In the case of comorbid pulmonary edema, the accuracy was 100% for COVID-19 and 100% for OLD. Both COVID-19 and OLD showed 100% accuracy for comorbid lung tumors. In the case of concomitant old inflammatory changes, the accuracy was 100% for COVID-19, 91.7% for the OLD, and 97.6% for the normal. In other comorbidities (e.g., hiatal hernia of the esophagus, atelectasis), the correct response rates were 80.0% for COVID-19, 100% for OLD, and 100% for normal. The accuracy for COVID-19 without comorbidity was 92.7%, 90.9% for OLD, and 98.5% for normal. The accuracy was particularly low for COVID-19 and OLD patients with pulmonary emphysema.

Table 13.4 (d) shows the accuracy for each disease. It was 90.0% for COVID-19. For OLD, the accuracy was 93.8% for bacterial pneumonia, 85.2% for ILD, 91.3% for viral pneumonia, 75.0% for atypical pneumonia, 100% for cardiogenic pulmonary edema, 100.0% for COPD, and 87.5% for others. Normal cases had an accuracy of 98.5%. The results showed lower accuracy for ILD and atypical pneumonia.

**Table 13.4: Comparison of accuracies**

Each value is expressed as a percentage of the accuracy. The numbers in parentheses indicate the number of correct cases divided by the total number of cases.

**(a): Comparison of accuracies by patient sex.**

|  | **COVID-19** | **OLD** | **Normal** |
| --- | --- | --- | --- |
| Male | 89.4 (76/85) | 89.8 (88/98) | 97.1 (68/70) |
| Female | 91.4 (32/35) | 93.1 (54/58) | 100.0 (60/60) |

**(b): Comparison of accuracies by patient age.**

|  | **COVID-19** | **OLD** | **Normal** |
| --- | --- | --- | --- |
| 18–39 | 100.0 (2/2) | 90.0 (9/10) | 100.0 (30/30) |
| 40–59 | 87.5 (21/24) | 93.3 (14/15) | 96.3 (26/27) |
| 60–79 | 94.0 (63/67) | 88.6 (62/70) | 100.0 (47/47) |
| 80– | 81.5 (22/27) | 93.4 (57/61) | 96.2 (25/26) |

**(c): Comparison of accuracies by comorbidities.**

|  | **COVID-19** | **OLD** | **Normal** |
| --- | --- | --- | --- |
| Bacterial pneumonia | 100.0 (6/6) | 100.0 (6/6) |  |
| ILD |  | 100.0 (1/1) |  |
| Pulmonary emphysema | 79.2 (19/24) | 83.3 (20/24) | 100.0 (18/18) |
| Pleural effusion | 87.1 (27/31) | 94.3 (33/35) | 100.0 (5/5) |
| Pulmonary edema | 100.0 (4/4) | 100.0 (6/6) |  |
| Pulmonary tumor | 100.0 (2/2) | 100.0 (10/10) |  |
| Old inflammatory changes | 100.0 (22/22) | 91.7 (11/12) | 97.6 (41/42) |
| Others | 80.0 (4/5) | 100.0 (8/8) | 100.0 (1/1) |
| No comorbidities | 92.7 (51/55) | 90.9 (70/77) | 98.5 (66/67) |

**(d): Comparison of accuracies by detailed findings.**

|  | **COVID-19** | **OLD** | **Normal** |
| --- | --- | --- | --- |
| COVID-19 | 90.0 (108/120) |  |  |
| Bacterial pneumonia |  | 93.8 (76/81) |  |
| ILD |  | 85.2 (23/27) |  |
| Viral pneumonia |  | 91.3 (21/23) |  |
| Atypical pneumonia |  | 75.0 (3/4) |  |
| Cardiogenic pulmonary edema |  | 100.0 (3/3) |  |
| COPD |  | 100.0 (2/2) |  |
| Others |  | 87.5 (14/16) |  |
| Normal |  |  | 98.5 (128/130) |

## Supplementary Section 14: Failure analysis

We analyzed the characteristics of the cases misclassified by the two models for the test data.

### Breakdown of the misclassified cases of the series model

The series model with a threshold of 0.5 misclassified 28 cases (12 COVID-19 positive and 16 negative cases) out of 406 cases (120 COVID-19 positive and 286 negative cases). Table 14.1 lists the disease details of the 28 misclassified cases. Each case is assigned an ID for use in the analysis described below, with series model Error ID 1–12 being COVID-19 positive cases and Error ID 13–28, negative cases. Table 14.1 lists the case ID (series model error ID), name of the disease, and comorbidities.

Among these cases, 12 were false negatives, five had pulmonary emphysema, four had pleural effusions, and one had a hiatal hernia. Sixteen false-positive cases were as follows: five bacterial pneumonia, two viral pneumonia, one atypical pneumonia, five ILD, one lung tumor, and two normal cases. Among the false-positive cases, four had pulmonary emphysema, two had pleural effusions, and two had inflammatory changes.

### Analysis of the misclassification of the series model

The characteristics of the 28 cases misclassified by the series model are analyzed and classified into the following four patterns:

1. Both false-negative and false-positive cases with pleural effusion or structural changes in the lung, such as emphysema, bulla, significant fibrosis, and other old inflammatory changes (both COVID-19 positive and negative cases).
2. COVID-19 positive cases were noted as nonspecific by radiologists, i.e., false negatives.
3. False-positive cases of ILD, including eosinophilic pneumonia, pneumocystis pneumonia, drug-induced interstitial pneumonia, and silicosis.
4. False-negative and false-positive cases attributed to insufficient training of the models.

The failure analysis of the 12 cases in which the series model misclassified COVID-19 positive cases as negative is detailed as follows.

Figure 14.1 (a) and (b) are classified as (1). Figure 14.1 (a) shows the cases with ID No. 7 at the top and No. 12 at the bottom of Table 14.1. In these cases, the characterization of COVID-19 may be complex because of the structural changes in the lungs. Similar considerations apply to ID Nos. 2, 4, 5, and 11. Figure 14.1 (b) shows the case with ID No. 1 at the top and No. 6 at the bottom. In these cases, the area of identifiable lesions is small because of pleural effusions, which makes it challenging to characterize COVID-19. Figure 14.1 (c) is classified as (2), with ID No. 9 at the top and No. 10 at the bottom. These cases are believed to be misclassified because the imaging features are nonspecific as COVID-19. Figure 14.1 (d) is classified as (4), with ID No. 3 at the top and No. 8 at the bottom. These cases are diagnosed as typical for COVID-19; a lack of training in the model may have caused the misclassification.

**Table 14.1 Failure cases of the series model with a threshold of 0.5.**

| **Series model Error ID** | **Pneumonia** | **Comorbidities** |
| --- | --- | --- |
| 1 | COVID-19 | Pleural effusions |
| 2 | COVID-19 | Pulmonary emphysema |
| 3 | COVID-19 |  |
| 4 | COVID-19 | Pulmonary emphysema |
| 5 | COVID-19 | Pulmonary emphysema |
| 6 | COVID-19 | Pleural effusions |
| 7 | COVID-19 | Pleural effusions, Pulmonary emphysema |
| 8 | COVID-19 |  |
| 9 | COVID-19 |  |
| 10 | COVID-19 | Pleural effusions, Other (Hiatal hernia) |
| 11 | COVID-19 | Pulmonary emphysema |
| 12 | COVID-19 |  |
| 13 | OLD—Bacterial pneumonia (aspiration) |  |
| 14 | OLD—Viral pneumonia |  |
| 15 | OLD—Atypical pneumonia |  |
| 16 | OLD—ILD |  |
| 17 | OLD—Bacterial pneumonia | Old inflammatory changes |
| 18 | OLD—ILD |  |
| 19 | OLD—ILD |  |
| 20 | OLD—ILD | Pulmonary emphysema |
| 21 | OLD—Lung tumor | Pleural effusions |
| 22 | OLD—Bacterial pneumonia | Pulmonary emphysema |
| 23 | OLD—Viral pneumonia | Pulmonary emphysema |
| 24 | OLD—ILD |  |
| 25 | OLD—Bacterial pneumonia | Pulmonary emphysema |
| 26 | OLD—Bacterial pneumonia (aspiration) | Pleural effusions |
| 27 | Normal |  |
| 28 | Normal | Old inflammatory changes |

The following 16 cases are false positives.

Figure 14.2 (a) is classified as (1), with ID No. 17 at the top and No. 25 at the bottom in Table 14.1. These cases are considered complicated to classify because of the structural changes in the lungs. Similar considerations apply to the cases with ID Nos. 20, 22, and 28. Figure 14.2 (b) is classified as (1), with ID No. 21 at the top and No. 26 at the bottom in Table 14.1. These cases are considered challenging to classify because of pleural effusion. Figure 14.2 (c) is classified as (3) with ID No. 15 at the top and No. 24 at the bottom. These are ILDs, and the similarity of the imaging findings to COVID-19 may have caused misclassification. Similar considerations apply to ID Nos. 16, 18, 19, and 23. Figure 14.2 (d) is classified as (4), with ID no. 13 at the top and no. 27 at the bottom. These were OLD or normal cases with small amounts of pleural effusion, obsolete inflammatory changes, and poor inspiration; a lack of training in the model may have caused the misclassification. Similar considerations were applied for ID No. 14.


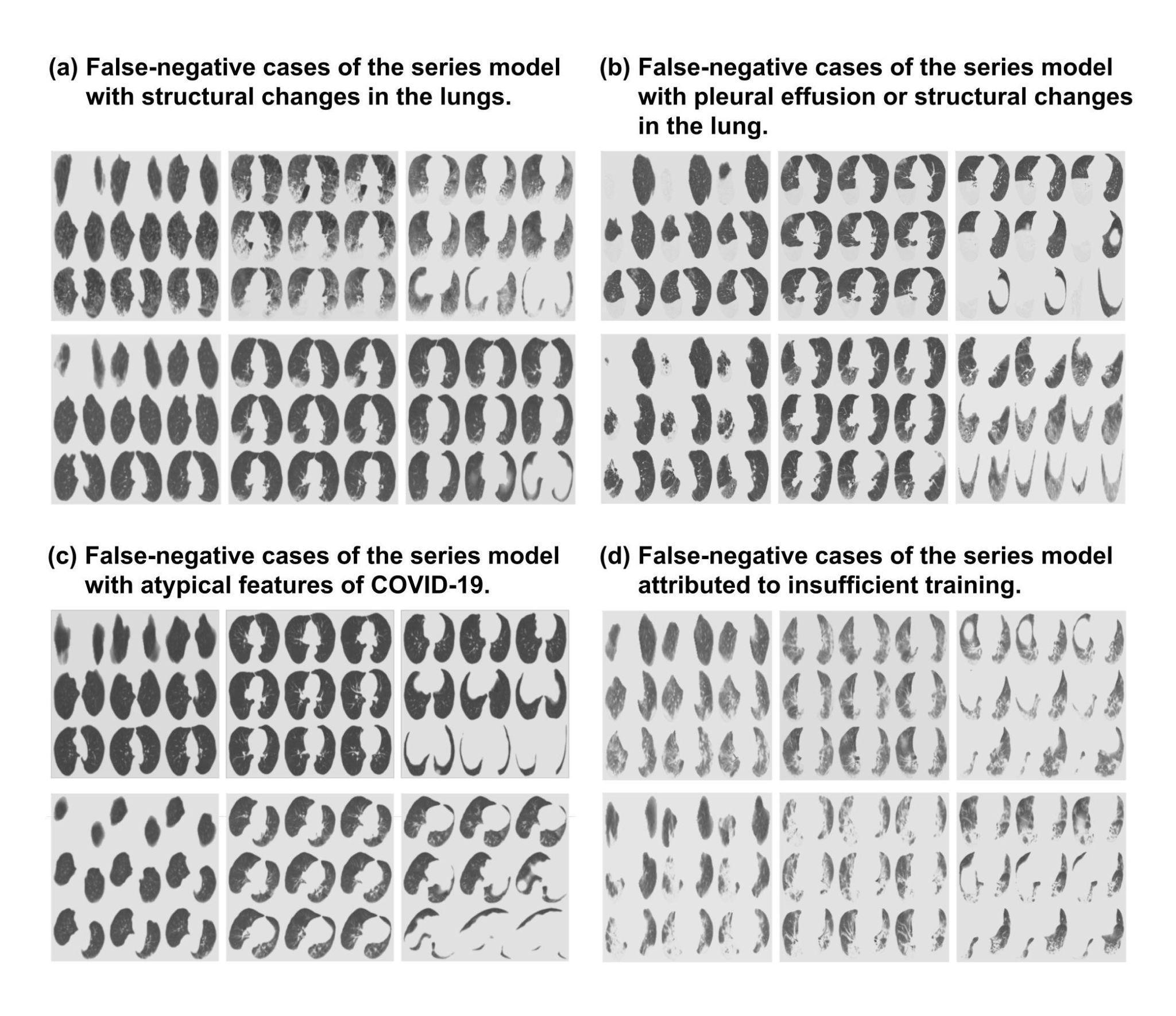


**Figure 14.1 False-negative cases of the series model:**Two examples were presented for each pattern. The images are the inputs to the series model after pre-processing.


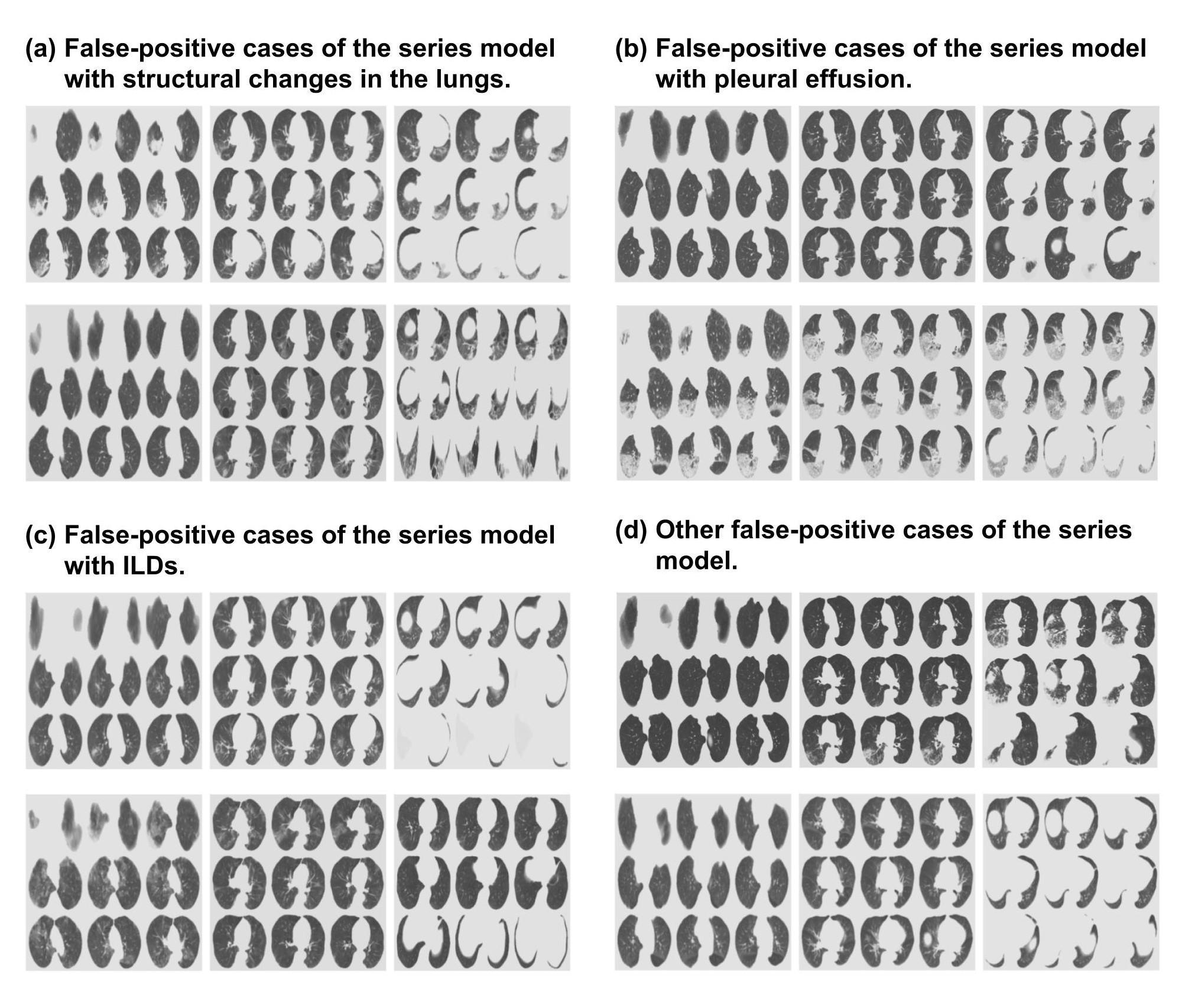


**Figure 14.2 False-positive cases of the series model:** Two examples were presented for each pattern. The images are the inputs to the series model after pre-processing.

### Breakdown of the misidentified cases of the slice model

With a threshold of 0.5, the slice model was incorrect in 1,620 slices (8.8%). Of these, 996 slices were false negative, and 654 were false positive. There were 40 cases (9.8%) in which the slice model was highly misidentified: more than 20% of slices of the series or more than 50% of all COVID-19 positive slices of the case. In addition, the series model misclassified seven positive and eight negative cases, and there was a high percentage of misidentification with the slice model.

The slice model misidentification included images of the lesion’s upper and lower edges, the lung’s apex, and the lung base, as shown in Figure 14.3. Radiologists at a radiology conference at the Osaka General Medical Center judged these to be challenging to differentiate from a single-slice image because of the small lesion area.

Slice model misclassification often included images of the boundary between the lesion and normal areas, the apex of the lung, and the lung base, as shown in Figure 14.3. The figure shows four cases, each different from the others in vertical alignment. For each case, the original image of the slice in which the misclassification occurred is presented on the left, and the input data for the slice model, which includes adjacent slices, are shown on the right. The first line of the figure shows an example in which the model misclassified a slice with a negative label as positive in a COVID-19 positive case. The second line shows an example in which the model misclassified a slice with a positive label as negative in a COVID-19 positive case. The third and fourth lines show examples of slices in a COVID-19 negative case misclassified as positive. Even the experts cannot determine whether a slice is COVID-19 positive or negative only by looking at the corresponding slice, and it is not surprising that the model misclassifies it.

Cases with a high misidentification rate were selected and analyzed on a case-by-case basis rather than on a slice-by-slice basis. The cases to be analyzed were as follows:

1. COVID-19 positive cases in which more than 50% of all slices with a positive label were misclassified.
2. Negative cases misclassified in more than 20% of all slices.


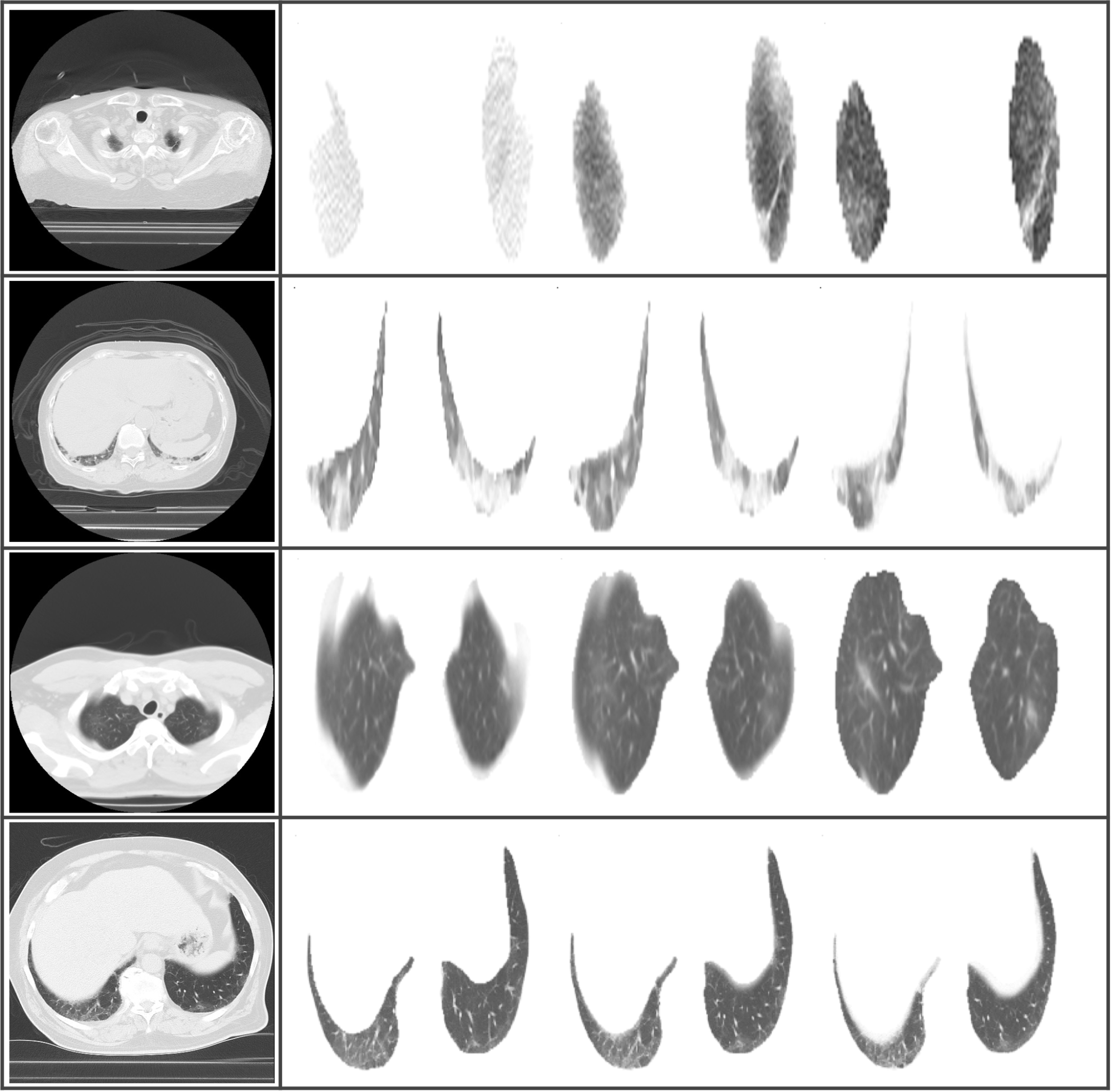


**Figure 14.3 Failure cases of the slice model at the top and bottom of the lungs:** The images on the left show the original slices misclassified by the slice model. The images on the right show the inputs to the slice model after pre-processing, which include the slices before and after the target images.

Table 14.2 (a) shows the false-negative cases, and Table 14.2 (b) shows the false-positive cases under the conditions above. The number of misidentified cases in the slice model was 40 (20 COVID-19 positive and 20 negative cases) out of 406 (120 COVID-19 positive and 286 negative cases).

Table 14.2 (a) indicates that the comorbidities of the 20 false-negative cases included six cases of pulmonary emphysema, eight cases of pleural effusion, three cases of bacterial pneumonia (including one aspiration), one case of pulmonary edema, three old inflammatory changes, and two other cases (one lung abscess and one hiatal hernia). The 20 false-positive cases included seven cases of bacterial pneumonia (five aspirations), two cases of viral pneumonia, eight cases of ILD, one case of cardiogenic pulmonary edema, one case of other (pulmonary tuberculosis), and one normal case. Comorbidities included pulmonary emphysema in cases, pleural effusion in six cases, bacterial pneumonia in one case, lung tumor in another, and old inflammatory changes in four cases. Seven of the 20 false-negative slice model cases were false-negative for the series model. Eight of the 20 false-positive slice model cases were false-positive for the series model.

**Table 14.2 Failure cases of the slice model with a threshold of 0.5:** “Slice model Error ID,” “Comorbidities,” and “Series model Error ID” represent IDs referred to in the main text, comorbidities the patients had, and IDs regarding Table 14.1 if the series model is also misclassified, respectively.

**(a): False-negative cases of the slice model with a threshold of 0.5.**

| **Slice model Error ID** | **Pneumonia** | **Comorbidities** | **Series model Error ID** |
| --- | --- | --- | --- |
| 1 | COVID-19 | Bacterial pneumonia (aspiration), Pleural effusion, Pulmonary emphysema |  |
| 2 | COVID-19 | Pleural effusion | 1 |
| 3 | COVID-19 |  |  |
| 4 | COVID-19 | Other (Lung abscess) |  |
| 5 | COVID-19 | Pulmonary emphysema | 2 |
| 6 | COVID-19 |  | 3 |
| 7 | COVID-19 | Pleural effusion |  |
| 8 | COVID-19 | Old inflammatory changes |  |
| 9 | COVID-19 | Bacterial pneumonia |  |
| 10 | COVID-19 | Pleural effusion, Old inflammatory changes |  |
| 11 | COVID-19 | Pulmonary emphysema, Old inflammatory changes |  |
| 12 | COVID-19 | Bacterial pneumonia, Pleural effusion |  |
| 13 | COVID-19 | Pulmonary edema |  |
| 14 | COVID-19 | Pleural effusion, Pulmonary emphysema | 7 |
| 15 | COVID-19 | Pleural effusion, Pulmonary emphysema |  |
| 16 | COVID-19 | Pleural effusion, Pulmonary emphysema |  |
| 17 | COVID-19 |  | 8 |
| 18 | COVID-19 |  | 9 |
| 19 | COVID-19 | Pleural effusion, Other (Hiatal hernia) | 10 |
| 20 | COVID-19 | Pleural effusion |  |

**(b): False-positive cases of the slice model with a threshold of 0.5.**

| **Slice model Error ID** | **Pneumonia** | **Comorbidities** | **Series model Error ID** |
| --- | --- | --- | --- |
| 21 | OLD—ILD | Pulmonary edema, Pleural effusion |  |
| 22 | OLD—Bacterial pneumonia (aspiration) | Pleural effusion |  |
| 23 | OLD—ILD |  | 16 |
| 24 | OLD—Bacterial pneumonia | Old inflammatory changes | 17 |
| 25 | OLD—ILD |  | 19 |
| 26 | OLD—Viral pneumonia |  |  |
| 27 | OLD—Bacterial pneumonia (aspiration) | Pulmonary emphysema, Old inflammatory changes |  |
| 28 | OLD—ILD | Lung tumor, Pleural effusion, Pulmonary emphysema |  |
| 29 | OLD—ILD | Pulmonary emphysema | 20 |
| 30 | OLD—Bacterial pneumonia | Pulmonary emphysema | 22 |
| 31 | OLD—Other (Pulmonary tuberculosis) |  |  |
| 32 | OLD—ILD |  | 24 |
| 33 | OLD—Bacterial pneumonia (aspiration) | Pleural effusion |  |
| 34 | OLD—Bacterial pneumonia (aspiration) | Pleural effusion |  |
| 35 | OLD—ILD | Pulmonary emphysema |  |
| 36 | OLD—Bacterial pneumonia (aspiration) | Pleural effusion | 26 |
| 37 | OLD—Viral pneumonia |  |  |
| 38 | OLD—Cardiogenic pulmonary edema | Bacterial pneumonia |  |
| 39 | OLD—ILD |  |  |
| 40 | Normal | Old inflammatory changes | 28 |

### Analysis of the misidentification of the slice model

The 40 cases misidentified by the slice model can be classified into four patterns similar to those of the series model.

1. Both false-negative and false-positive cases with pulmonary edema, pleural effusion, or structural changes in the lung, such as emphysema, bulla, significant fibrosis, and other old inflammatory changes (both COVID-19 positive and negative cases).
2. COVID-19 positive cases noted as nonspecific by radiologists, i.e., false negative.
3. False-positive ILD cases, including eosinophilic pneumonia, pneumocystis pneumonia, drug-induced interstitial pneumonia, and silicosis.
4. False-negative and false-positive cases attributed to a lack of learning.

An analysis of the 20 cases in which the slice model showed false negatives is presented below.

Figure 14.4 (a) and (b) are classified as (1). Figure 14.4 (a) shows cases with ID No. 4 at the top and No. 11 at the bottom of Table 14.2 (a). In these cases, the characterization of COVID-19 may be problematic because of the structural changes in the lungs. Similar considerations apply to ID Nos. 1, 5, 8, 14, and 15. Figure 14.4 (b) shows the case with ID No. 2 at the top and No. 13 at the bottom. In these cases, the area of identifiable lesions is small because of pleural effusions, which makes it challenging to characterize COVID-19. Similar considerations apply to ID Nos. 7, 10, and 16. Figure 14.4 (c) is classified as (2), with ID No. 18 at the top and No. 19 at the bottom. These cases are believed to be misidentified because the imaging features are nonspecific as COVID-19. Similar considerations apply to ID No. 12. Figure 14.4 (d) is classified as (4), with ID No. 6 at the top and No. 17 at the bottom. These cases were diagnosed as typical for COVID-19, and a lack of training in the model may have caused misidentification. Similar considerations apply to ID Nos. 3, 9, and 20.

The following 20 cases are slice models that showed false positives.

Figure 14.5 (a) is classified as (1), with ID No. 24 at the top and No. 30 at the bottom in Table 14.2 (b). These cases are considered complex to classify because of the structural changes in the lungs. Similar considerations apply to the cases with ID Nos. 29, 35, and 40. Figure 14.5 (b) is classified as (1), with ID No. 36 at the top and No. 38 at the bottom in Table 14.2 (b). These cases are considered challenging to classify because of pleural effusion. Similar considerations apply to ID Nos. 21, 22, 28, 33, and 34.

Figure 14.5 (c) is classified as (3), with ID No. 23 at the top and No. 32 at the bottom. These are ILDs, and the similarity of the imaging results to COVID-19 may have caused misidentification. Similar considerations apply to ID Nos. 25, 26, 37, and 39. Figure 14.5 (d) is classified as (4), with ID No. 27 at the top and No. 31 at the bottom. These were OLD or normal cases with small pleural effusion, obsolete inflammatory changes, and poor inspiration.


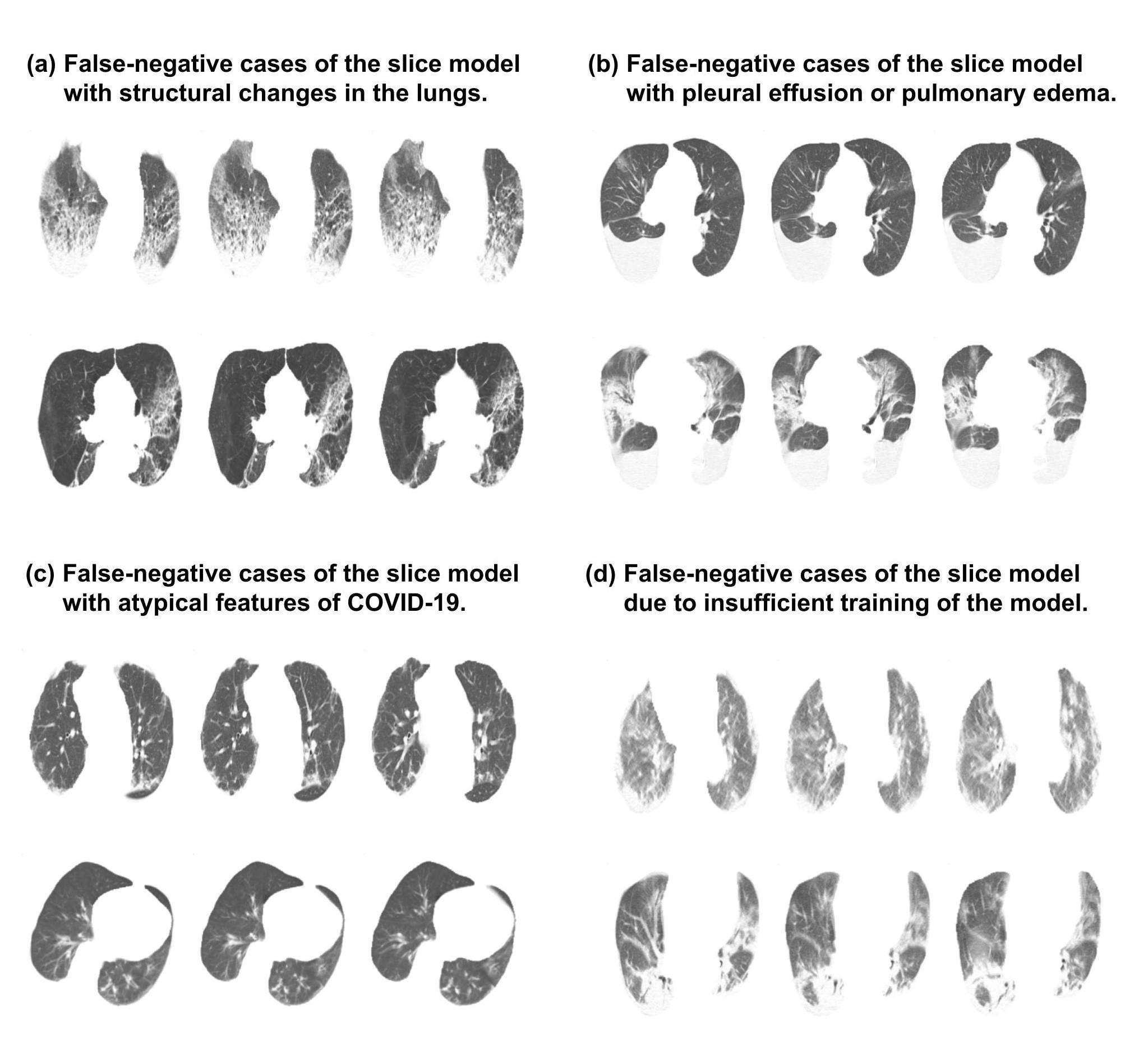


**Figure 14.4 False-negative cases of the slice model:** Two examples were presented for each pattern. The images are inputs to the slice model after pre-processing. The top and bottom images are not necessarily obtained from the same patient.


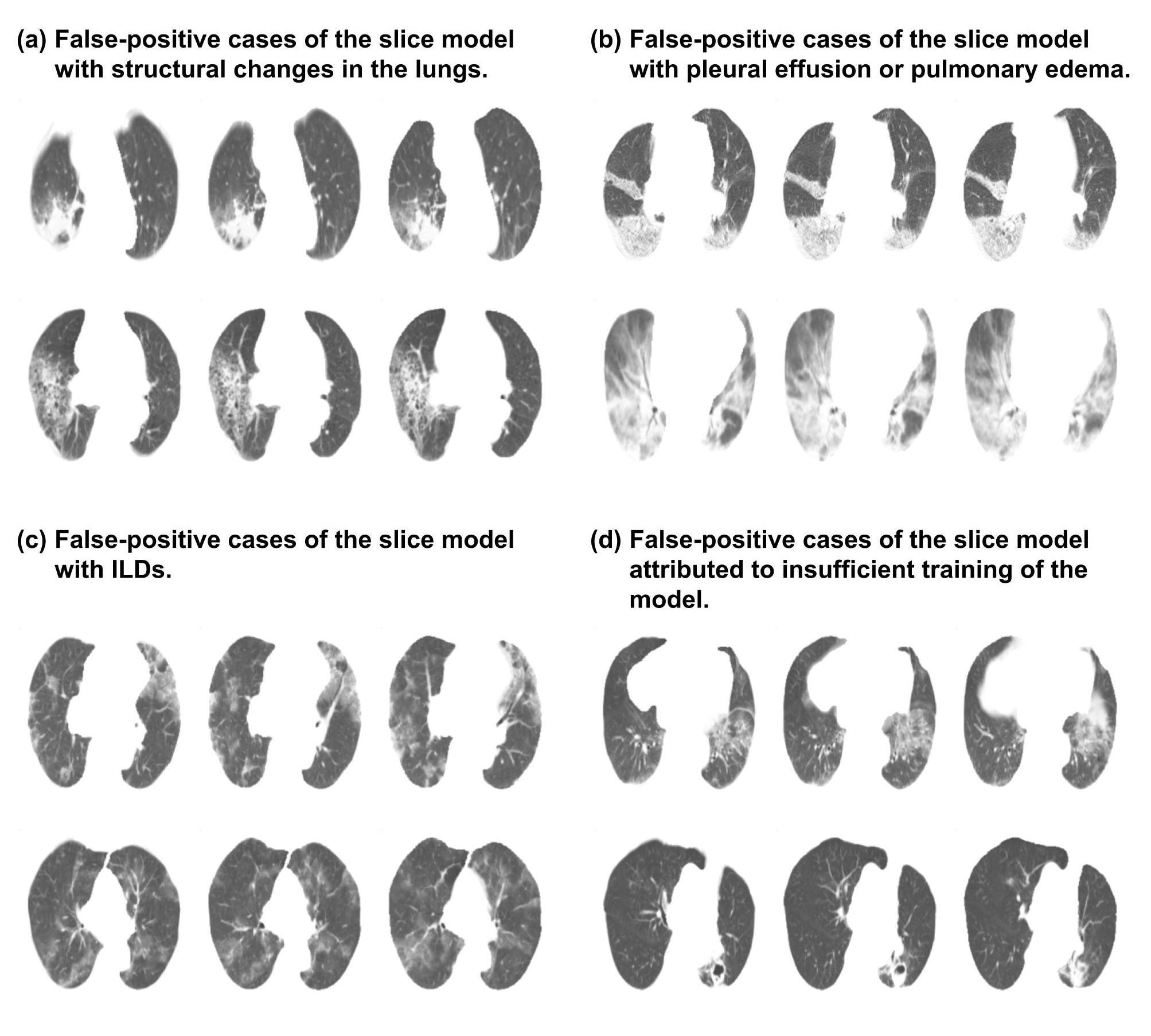


**Figure 14.5 False-positive cases of the slice model**

## Supplementary Section 15: Performance of the inference process

We measured the inference process performance using test data in the inference environment described in Supplementary Section 10.

### Choice of batch size

We conducted performance measurements to select the optimal batch sizes in the inference process before measuring the final inference process performance. This performance measurement targets lung field detection and slice models included in the pre-processing. The series model is executed only once for each series, and the batch size is always one; therefore, it is excluded from this performance measurement.

Figure 15.1 shows the average inference time per series, maximum system memory consumption, and maximum GPU memory consumption for different batch sizes of lung-field detection. Figure 15.2 shows the average inference time per series, maximum system memory consumption, and maximum GPU memory consumption for the different batch sizes of the slice model.

This performance measurement targets all test data. All slices of each series were input for lung field detection, and all slices with detected lung fields were input for the slice model. Therefore, lung field detection was the fastest, with a batch size of two at an average of 1.98 s. The execution of the slice model was the fastest, with a batch size of 64 at an average of 0.25 s.


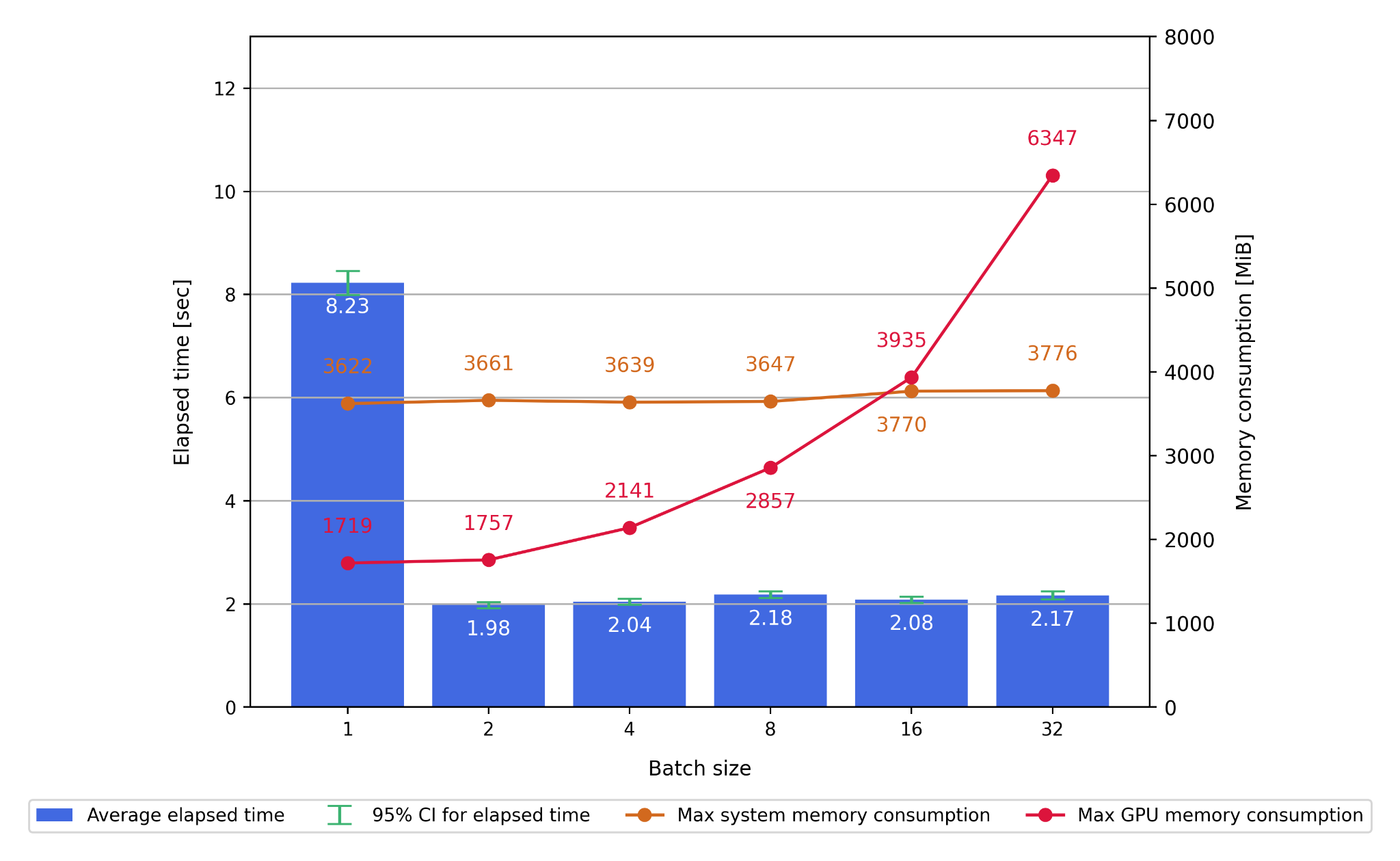


**Figure 15.1 Inference performance of lung field detection for different batch sizes:** “Average elapsed time” represents the average time taken for lung field detection per series. “Max system memory consumption” and “Max GPU memory consumption” represent the maximum memory consumption throughout the entire inference process with the test dataset.


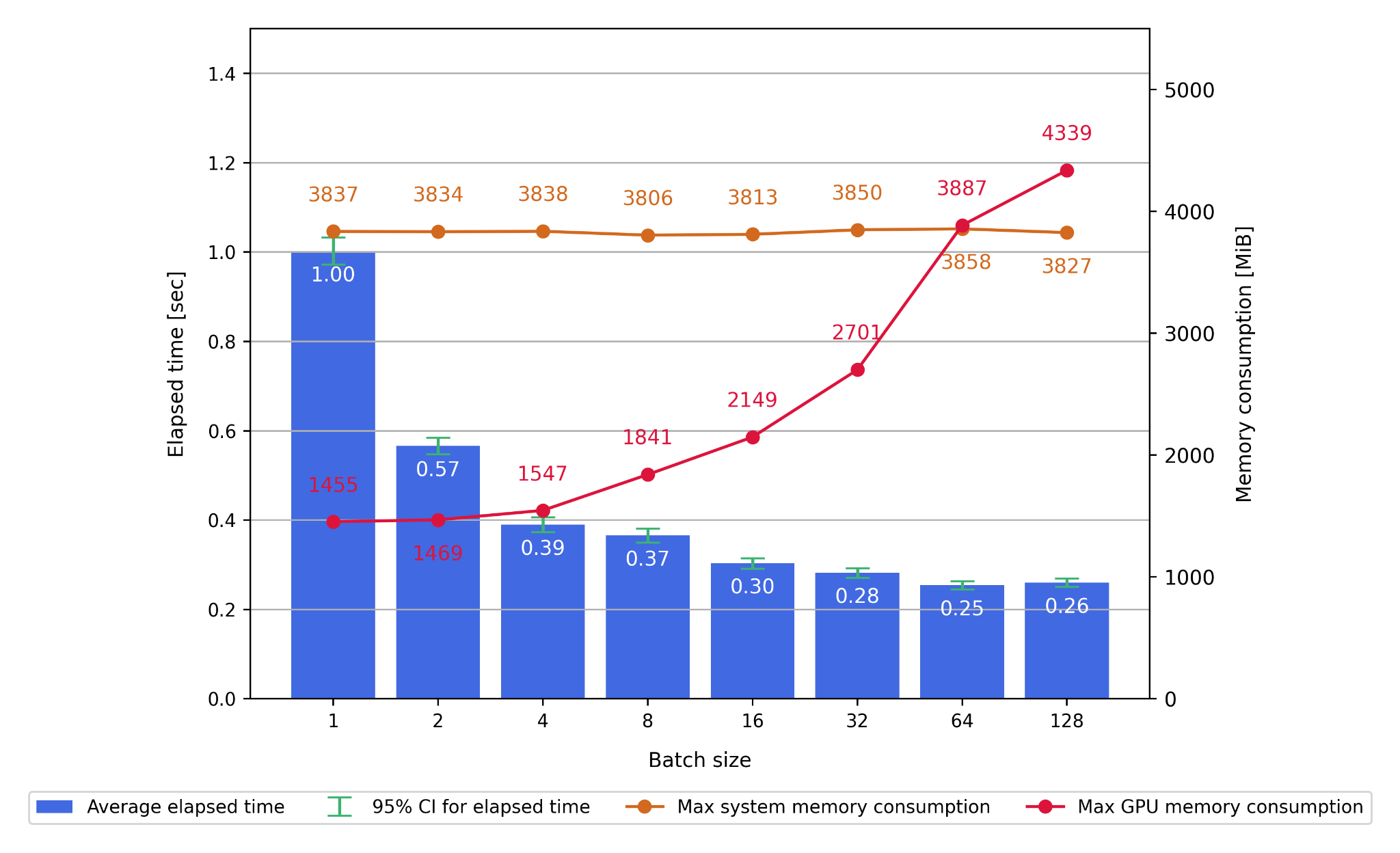


**Figure 15.2 Inference performance of the slice model for different batch sizes:** “Average elapsed time” represents the average time taken for lung field detection per series. “Max system memory consumption” and “Max GPU memory consumption” represent the maximum memory consumption throughout the entire inference process with the test dataset.

### Performance results

The comparison of the performances of our inference process, series, and slice models when executed independently is presented in Table 15.1. When we performed inference for each model independently, the series model output resulted in an average of 2.58 s (95% CI: 2.53–2.63) per series, a maximum of 3584 MiB of system memory consumption, and a maximum of 1639 MiB of GPU memory consumption after the DICOM data of the test dataset were given as input. The slice model output resulted in an average of 11.31 s (95% CI: 11.11–11.51) per series, a maximum of 3485 MiB of system memory consumption, and a maximum of 1511 MiB of GPU memory consumption after the DICOM data of the test dataset were given as input.

In contrast, our implementation of the inference process obtained outputs for both the slice and series models from the same data in an average of 2.83 s (95% CI: 2.79–2.88) per series, with a maximum consumption of 3680 MiB of system memory and 3961 MiB of GPU memory.

**Table 15.1 Performance comparison of our inference process, series model, and slice model when run independently.**

|  | **Average elapsed time [s] (95% CI)** | **Max system memory consumption [MiB]** | **Max GPU memory consumption [MiB]** |
| --- | --- | --- | --- |
| Our inference processing | 2.83 (2.79–2.88) | 3680 | 3961 |
| **Inference when running both models independently** | | | |
| Series model | 2.58 (2.53–2.63) | 3584 | 1639 |
| Slice model | 11.31 (11.11–11.51) | 3485 | 1511 |

## Supplementary Section 16: Imaging diagnostic application software

We developed and released an application software that allows model implementation without modification. The application environment can be built by installing Python 3.7.4, and the Python packages are presented in Table 16.1. The video of this application software is available on Zenodo (<https://doi.org/10.5281/zenodo.5835313>).

This application runs in the environment described in Supplementary Section 10 and is built using Kivy (version 2.0.0) module from Python. Kivy supports operation in Android, iOS, Linux, macOS X, and Windows. The inference application has been tested only in the environment described in Supplementary Section 10. The source code of this application is available on Zenodo (<https://doi.org/10.5281/zenodo.5835313>), along with detailed instructions for building the operating environment.

After the application is built according to the published instructions, the “COVID-19 Recognizer” icon is placed on the desktop, and double-clicking this icon can start the application. A GUI for the user operation is displayed on the screen when the application is launched; the docker for executing the inference process is also launched. Figure 16.1 shows the screen at the start of the application.

When entering case data, it is necessary to drag and drop the directory containing the CT images taken from the file system onto the application. The CT images are displayed on the left side of the screen when the data are entered, and the inference process is executed simultaneously. The screen immediately looks like Figure 16.2, and the progress bar at the bottom shows the state of the image-inference process. The output results are sent to the application, and the contents are displayed on the right half of the screen when the inference process is completed.

**Table 16.1 Python packages for application software**

| **Package name** | **Version** |
| --- | --- |
| zwaddict | 2.4.0 |
| gdcm | 1.1 |
| imagecodecs | 2021.8.26 |
| kivy | 2.0.0 |
| kivymd | 0.104.2 |
| numpy | 1.20.0 |
| opencv-contrib-python | 4.0.0.21 |
| pydicom | 2.1.2 |
| pylibjpeg | 1.1.1 |
| pylibjpeg-libjpeg | 1.1.0 |
| pycryptodome | 3.11.0 |
| pyzmq | 22.3.0 |

Figure 16.3 shows the screen after the inference process is completed. The series model output is displayed in the right half of the screen, and the slice model output corresponding to the image shown in the left half can be changed using the slider at the bottom. The window width and center of the CT image can be adjusted using the “w” button at the top-right. The CT value can be seen at that point by clicking on the image displayed in the left half. Drag and drop the target directory on the screen after the inference process is completed to perform an inference on a new case.

This application allows users to see the results of the inference process on the right half of the screen while viewing the CT images on the left half.


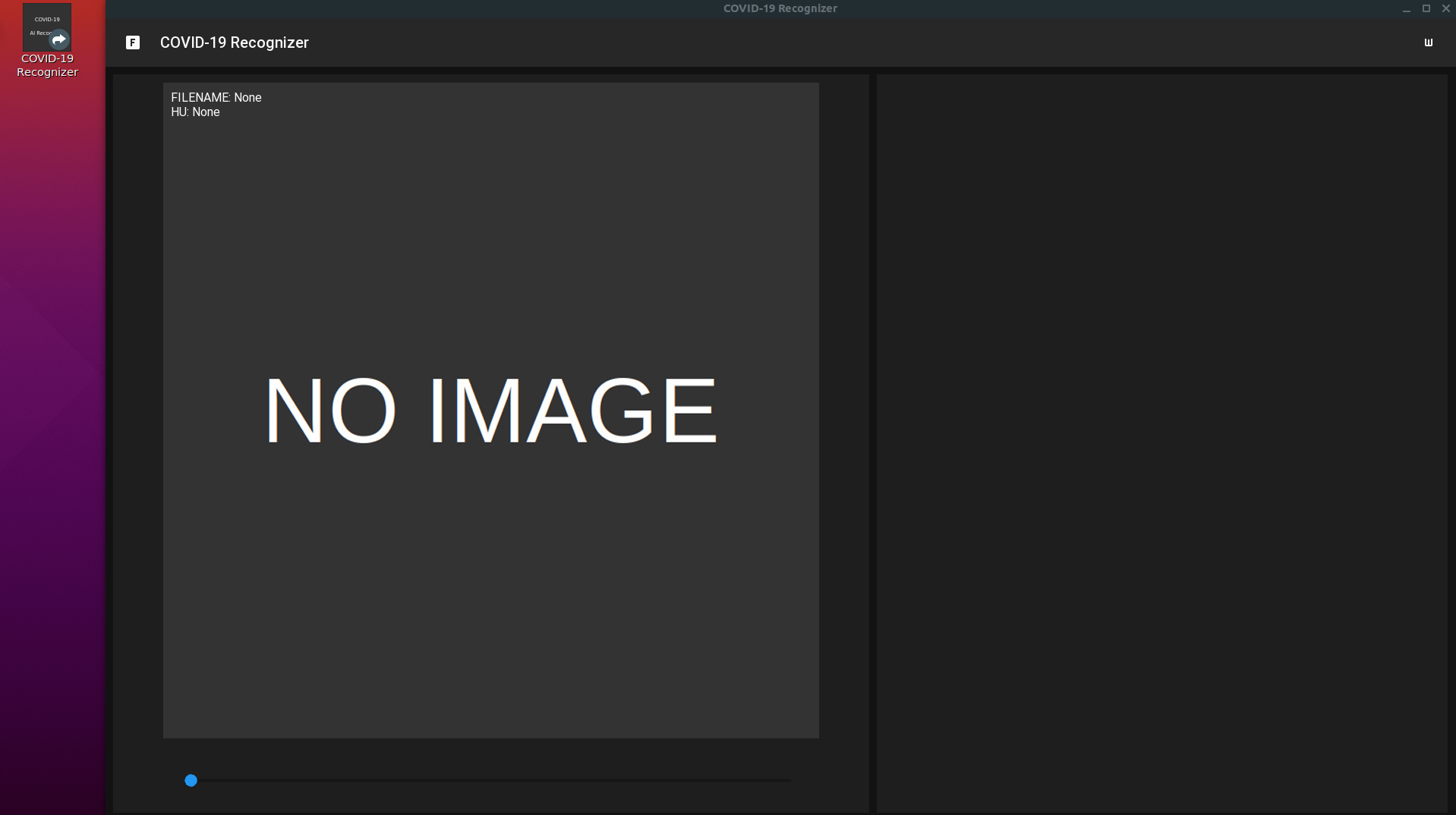


**Figure 16.1 Screenshot of our application at startup.**


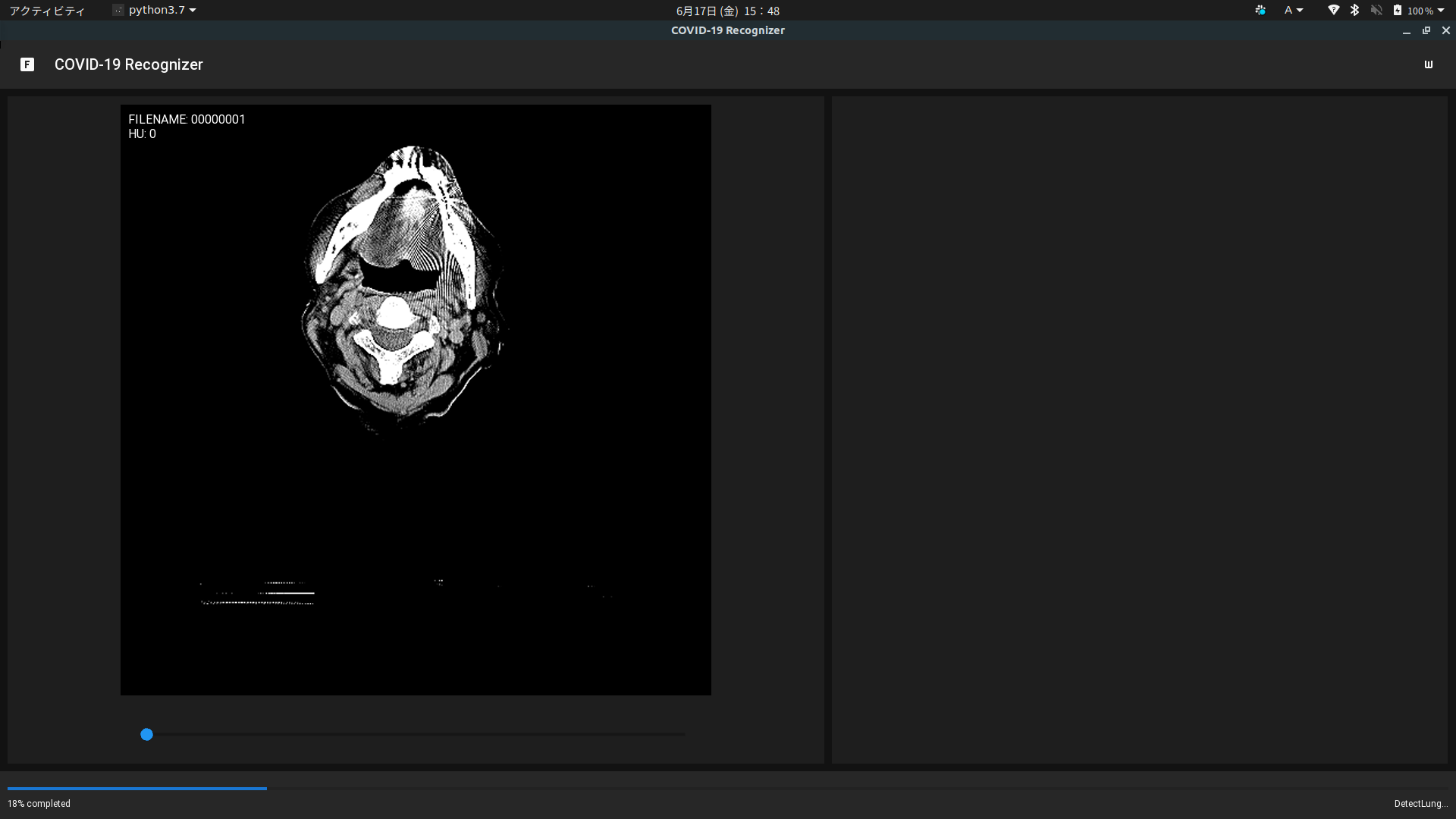


**Figure 16.2 Screenshot of our application after data entry:** The CT images are displayed on the left side of the screen, and the inference process is executed simultaneously.


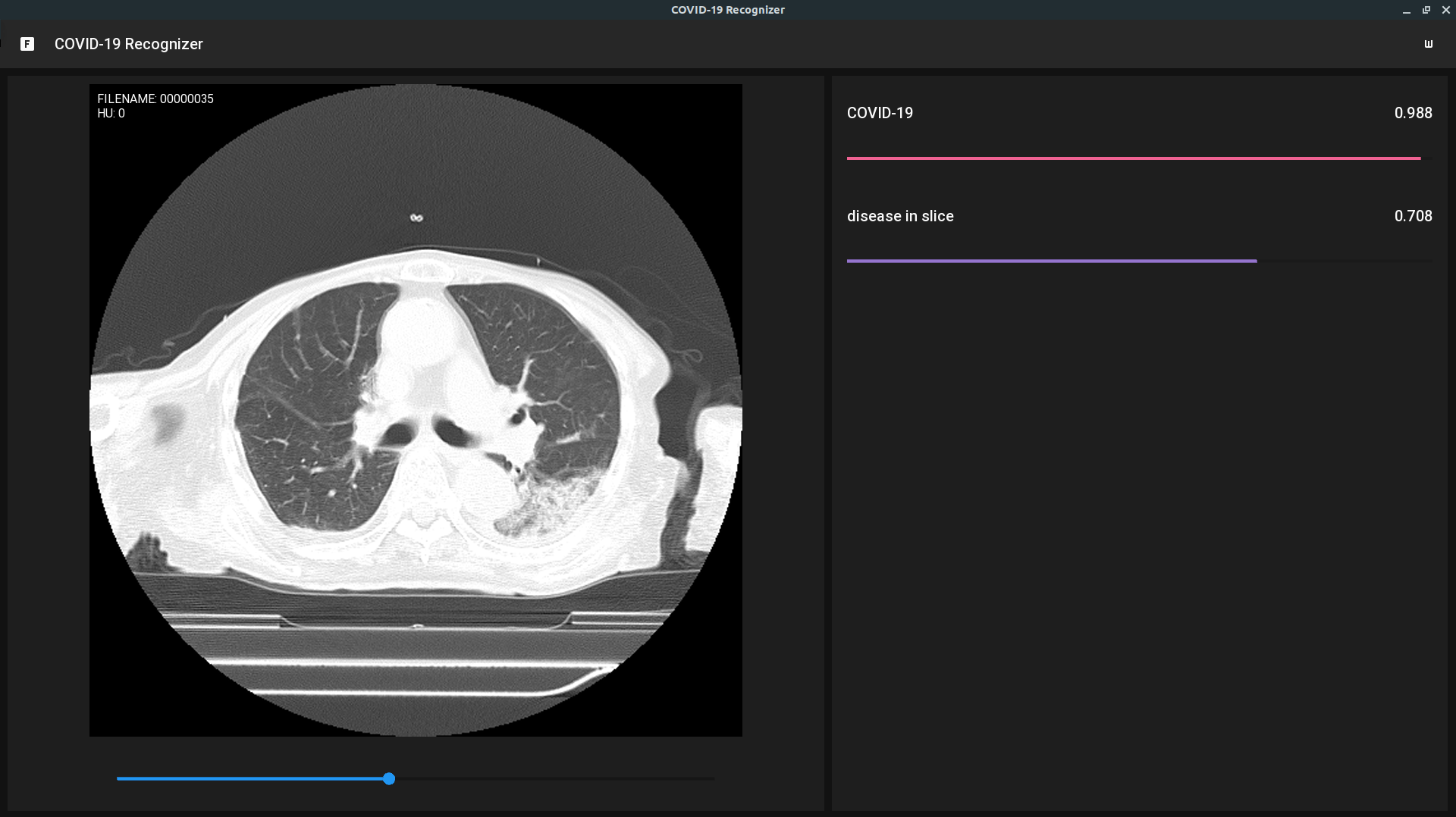


**Figure 16.3 Screenshot of our application after analysis is completed:** The outputs of the series and slice models are shown on the right-hand side of the screen.

## References

1. Hanley J. A. & McNeil B. J. The meaning and use of the area under a receiver operating characteristic (ROC) curve. Radiology 143, 29–36 (1982).

2. Mongan J., Moy L. & Kahn C. E. Jr. Checklist for Artificial Intelligence in Medical Imaging (CLAIM): A Guide for Authors and Reviewers. Radiol Artif Intell 2, e200029 (2020).

3. Lambin P. et al. Radiomics: the bridge between medical imaging and personalized medicine. Nat. Rev. Clin. Oncol. 14, 749–762 (2017).

4. Guo Q. et al. An Empirical Study Towards Characterizing Deep Learning Development and Deployment Across Different Frameworks and Platforms. 2019 34th IEEE/ACM International Conference on Automated Software Engineering (ASE) 810–822. (2019).

5. Sze V., Chen Y.-H., Yang T.-J. & Emer J. Efficient Processing of Deep Neural Networks. (Springer Nature, 2022).

6. Bianco S., Cadene R., Celona L., & Napoletano P. Benchmark Analysis of Representative Deep Neural Network Architectures. IEEE Access 6, 64270–64277 (2018).

7. Canziani A. & Paszke A. Culurciello E. An Analysis of Deep Neural Network Models for Practical Applications. Preprint at http://arxiv.org/abs/1605.07678 (2016).

8. Kang D., Mathur A., Veeramacheneni T., et al. Jointly Optimizing Preprocessing and Inference for DNN-based Visual Analytics. Preprint at http://arxiv.org/abs/2007.13005 (2020).

9. Larobina M., Murino L. Medical image file formats. J. Digit. Imaging 27, 200–206 (2014).

10. Willemink M. J. et al. Preparing Medical Imaging Data for Machine Learning. Radiology 295, 4–15 (2020).

11. Prokop M. et al. CO-RADS: A Categorical CT Assessment Scheme for Patients Suspected of Having COVID-19-Definition and Evaluation. Radiology 296, E97–E104 (2020).

12. Hofmanninger J. et al. Automatic lung segmentation in routine imaging is primarily a data diversity problem, not a methodology problem. Eur Radiol Exp 4, 50 (2020).

13. Qian N. On the momentum term in gradient descent learning algorithms. Neural Netw. 12, 145–151 (1999).

14. Kingma D. P. & Ba J. Adam: A Method for Stochastic Optimization. Preprint at http://arxiv.org/abs/1412.6980 (2014).

15. Loshchilov I. & Hutter F. Decoupled Weight Decay Regularization. Preprint at http://arxiv.org/abs/1711.05101 (2017).

16. Zhong Z., Zheng L., Kang G., Li S. & Yang Y. Random Erasing Data Augmentation. AAAI 34, 13001–13008 (2020).

17. Davies M. & Fleiss J. L. Measuring Agreement for Multinomial Data. Biometrics 38, 1047–1051 (1982).

18. McHugh M. L. Interrater reliability: the kappa statistic. Biochem. Med. 22, 276–282 (2012).

19. Landis J. R. & Koch G. G. The measurement of observer agreement for categorical data. Biometrics 33, 159–174 (1977).

20. Efron B. Bootstrap Methods: Another Look at the Jackknife. Ann. Stat. 7, 1–26 (1979).
